# SUMOylation controls Hu antigen R posttranscriptional activity in liver cancer

**DOI:** 10.1016/j.celrep.2024.113924

**Published:** 2024-03-18

**Authors:** Sofia Lachiondo-Ortega, Claudia M. Rejano-Gordillo, Jorge Simon, Fernando Lopitz-Otsoa, Teresa C. Delgado, Krystyna Mazan-Mamczarz, Naroa Goikoetxea-Usandizaga, L. Estefanía Zapata-Pavas, Ana Garcıía-del Río, Pietro Guerra, Patricia Peña-Sanfélix, Natalia Hermán-Sánchez, Ruba Al-Abdulla, Carmen Fernandez-Rodríguez, Mikel Azkargorta, Alejandro Velázquez-Cruz, Joris Guyon, César Martín, Juan Diego Zalamea, Leire Egia-Mendikute, Arantza Sanz-Parra, Marina Serrano-Maciá, Irene González-Recio, Monika Gonzalez-Lopez, Luis Alfonso Martínez-Cruz, Patrizia Pontisso, Ana M. Aransay, Rosa Barrio, James D. Sutherland, Nicola G.A. Abrescia, Félix Elortza, Amaia Lujambio, Jesus M. Banales, Raúl M. Luque, Manuel D. Gahete, Asıś Palazón, Matias A. Avila, Jose J. G. Marin, Supriyo De, Thomas Daubon, Antonio Díaz-Quintana, Irene Díaz-Moreno, Myriam Gorospe, Manuel S. Rodríguez, María Luz Martínez-Chantar

**Affiliations:** 1 Liver Disease Lab, Center for Cooperative Research in Biosciences (CIC bioGUNE), Basque Research and Technology Alliance (BRTA), 48160 Derio, Bizkaia, Spain; 2 Department of Biochemistry and Molecular Biology, Faculty of Sciences, University of Extremadura, University Institute of Biosanitary Research of Extremadura (INUBE), 06071 Badajoz, Spain; 3 Biofisika Institute, Consejo Superior de Investigaciones Científicas (CSIC), Departamento Bioquímica y Biología Molecular, Facultad de Ciencia y Tecnología, Universidad del País Vasco (UPV/EHU), Leioa, Spain; 4 Centro de Investigación Biomédica en Red de Enfermedades Hepáticas y Digestivas (CIBERehd), Carlos III National Health Institute, Madrid, Spain; 5 Laboratory of Genetics and Genomics, National Institute on Aging (NIA), Intramural Research Program (IRP), National Institutes of Health (NIH), Baltimore, MD, USA; 6 Cancer Immunology and Immunotherapy Lab, Center for Cooperative Research in Biosciences (CIC bioGUNE), Basque Research and Technology Alliance (BRTA), 48160 Derio, Bizkaia, Spain; 7 Unit of Internal Medicine and Hepatology (UIMH), Department of Medicine (DIMED), University of Padova, 35128 Padua, Italy; 8 Maimónides Institute of Biomedical Research of Córdoba (IMIBIC), Department of Cell Biology, Physiology and Immunology of University of Córdoba, Reina Sofia University Hospital, CIBER Pathophysiology of Obesity and Nutrition (CIBERobn), 14004 Córdoba, Spain; 9 Instituto de Investigación, Desarrollo e Innovación en Biotecnologıá Sanitaria de Elche (IDiBE), Universidad Miguel Hernández, Elche, Spain; 10 Institute of Medical Biochemistry and Molecular Biology, University Medicine of Greifswald, 17475 Greifswald, Germany; 11 Proteomics Platform, Center for Cooperative Research in Biosciences (CIC bioGUNE), Basque Research and Technology Alliance (BRTA), Carlos III Networked Proteomics Platform (ProteoRed-ISCIII), 48160 Derio, Bizkaia, Spain; 12 Instituto de Investigaciones Químicas (IIQ), Centro de Investigaciones Científicas Isla de la Cartuja (cicCartuja), Universidad de Sevilla, Consejo Superior de Investigaciones Científicas (CSIC), Sevilla, Spain; 13 University of Bordeaux, INSERM, BPH, U1219, 33000 Bordeaux, France; 14 CHU de Bordeaux, Service de Pharmacologie Médicale, 33000 Bordeaux, France; 15 Structure and Cell Biology of Viruses Lab, Center for Cooperative Research in Biosciences (CIC bioGUNE), Basque Research and Technology Alliance (BRTA), 48160 Derio, Bizkaia, Spain; 16 Genome Analysis Platform, Center for Cooperative Research in Biosciences (CIC bioGUNE), Basque Research and Technology Alliance (BRTA), 48160 Derio, Bizkaia, Spain; 17 Ubiquitin-likes and Development Lab, Center for Cooperative Research in Biosciences (CIC bioGUNE), Basque Research and Technology Alliance (BRTA), 48160 Derio, Bizkaia, Spain; 18 Ikerbasque, Basque Foundation for Science, Bilbao, Spain; 19 Department of Oncological Sciences, Icahn School of Medicine at Mount Sinai, New York, NY, USA; 20 Liver Cancer Program, Division of Liver Diseases, Department of Medicine, Tisch Cancer Institute, Icahn School of Medicine at Mount Sinai, New York, NY, USA; 21 The Precision Immunology Institute, Icahn School of Medicine at Mount Sinai, New York, NY, USA; 22 Graduate School of Biomedical Sciences at Icahn School of Medicine at Mount Sinai, New York, NY, USA; 23 Department of Liver and Gastrointestinal Diseases, Biodonostia Health Research Institute, Donostia University Hospital, San Sebastian, Spain; 24 Department of Biochemistry and Genetics, School of Sciences, University of Navarra, Pamplona, Spain; 25 Hepatology Program, Centro de Investigación Médica Aplicada (CIMA), University of Navarra, Pamplona, Spain; 26 Instituto de Investigaciones Sanitarias de Navarra (IdiSNA), Pamplona, Spain; 27 Experimental Hepatology and Drug Targeting (HEVEPHARM), Instituto de Investigació n Biomé dica de Salamanca (IBSAL), University of Salamanca, Salamanca, Spain; 28 University of Bordeaux, CNRS, IBGC, UMR 5095, Bordeaux, France; 29 Laboratoire de Chimie de Coordination (LCC), UPR 8241, CNRS; IPBS-University of Toulouse III-Paul Sabatier, Toulouse, France; 30 These authors contributed equally; 31 Lead contact

## Abstract

The posttranslational modification of proteins critically influences many biological processes and is a key mechanism that regulates the function of the RNA-binding protein Hu antigen R (HuR), a hub in liver cancer. Here, we show that HuR is SUMOylated in the tumor sections of patients with hepatocellular carcinoma in contrast to the surrounding tissue, as well as in human cell line and mouse models of the disease. SUMOylation of HuR promotes major cancer hallmarks, namely proliferation and invasion, whereas the absence of HuR SUMOylation results in a senescent phenotype with dysfunctional mitochondria and endoplasmic reticulum. Mechanistically, SUMOylation induces a structural rearrangement of the RNA recognition motifs that modulates HuR binding affinity to its target RNAs, further modifying the transcriptomic profile toward hepatic tumor progression. Overall, SUMOylation constitutes a mechanism of HuR regulation that could be potentially exploited as a therapeutic strategy for liver cancer.

## In brief

Lachiondo-Ortega et al. report that the RNA-binding protein HuR is posttranslationally modified by SUMOylation in the tumor tissue of patients with hepatocellular carcinoma. This mechanism of HuR regulation could be potentially exploited as a therapeutic strategy against liver cancer, thus highlighting the relevance of posttranslational modifications as disease targets.

## Graphical abstract

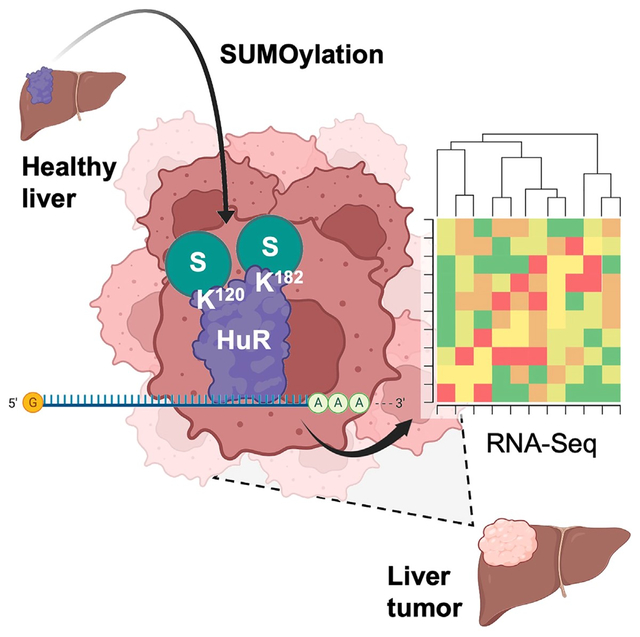

## INTRODUCTION

The molecular mechanisms underlying the malignant transformation of a healthy liver into hepatocellular carcinoma (HCC), the most common type of primary liver cancer, are numerous and highly heterogeneous.^1^ Therefore, it is currently believed that more than one signaling route would need to be drugged in order to stop the progression of the disease. At the moment, there are approved front- and second-line systemic treatments for advanced HCC available that are improving patient survival, and the number of agents found to be effective in phase 3 trials continues to grow.^2^ However, the empirical development of new drugs for HCC has not yielded the beneficial outcomes seen in other malignancies. To move the field forward meaningfully, we need outside-the-box approaches that adopt novel combination strategies and pursue new targets in HCC as we await the results of ongoing phase 3 clinical trials. In this context, the posttranslational modification (PTM) of proteins, which controls the specificity, timing, duration, and amplitude of virtually all physiological processes in the cell, is gaining momentum as a robust and multidimensional therapeutic strategy in cancer.^3^

SUMOylation is a ubiquitin-like (Ubl) PTM, conserved across eukaryotes, that consists in the covalent addition of one or multiple SUMO (small ubiquitin-like modifier) subunits or polymers to lysine residues of target proteins in a hierarchically organized process, thus contributing to the structural and functional diversity of the proteome. The SUMOylation cascade is catalyzed by a dimeric SUMO-activating enzyme E1 (SAE1/UBA2), a unique E2 ubiquitin-conjugating enzyme 9 (UBC9), and a handful of E3 ligases, including members of the protein inhibitor of activated signal transducer and activator of transcription (PIAS) family. This modification can be reversed by the action of deSUMOylating enzymes, among which the sentrin-specific protease (SENP) family of SUMO-specific isopeptidases stand out and are also required for the maturation of precursor SUMO proteins.^4^ In mammals, up to five SUMO family members exist, each encoded by a separate gene. SUMO1–3 are expressed in most tissues,^5^ whereas SUMO4^6^ and 5^7^ are less abundant and restricted to specific tissues. Notably, the amino acid sequences of mature SUMO2 and SUMO3 are nearly identical but only share ~50% sequence identity with SUMO1.^8^ Moreover, SUMO2 and 3 tend to form polymeric chains,^9,10^ representing 90% of SUMO polymers.^11^ Mixed SUMO chains have been also reported and usually include SUMO1 at the distal end.^10^ SUMO1 contains an inverted SUMO motif that enables chain formation but at lower efficiency.^12^ In addition to covalent SUMOylation, SUMO can bind proteins non-covalently via SUMO-interacting motifs (SIMs),^13^ which can be found in many SUMO substrates and SUMO E3 ligases. Importantly, ~90% of SUMO-binding proteins are also covalent SUMO substrates.^14^

SUMOylation has an important regulatory role for most nuclear processes, including transcription, RNA processing, DNA-damage response (DDR), nucleocytoplasmic transport, cell-cycle progression, proteostasis, and nuclear body assembly.^15^ SUMO can regulate the activity, function, fate, and subcellular localization of target proteins by changing substrate interactions with DNA, RNA, or other proteins. SUMOylation functions during development^16,17^ and controls different physiological processes in adult organisms.^18^ Hence, its absence or dysregulation has been associated with disease.^4,19^ There is growing evidence that proteins implicated in the SUMOylation cascade are abundant in multiple cancers.^3,20^ In particular, higher expression of the genes involved in SUMOylation had been earlier detected as a pattern shared among patients with an accelerated progression of HCC.^21^ To date, the upregulation of SUMO1,^22^ SUMO2,^23^ SAE1,^24^ UBA2,^25^ UBC9,^26–29^ PIAS1,^30^ PIAS2,^31^ PIAS4,^32,33^ SENP1,^34–36^ SENP5,^37^ SENP6,^38,39^ and SENP7^40^ in HCC and their value either as diagnostic or prognostic biomarkers have been described. Conversely, SENP2 expression is decreased in HCC and might play a role as a tumor suppressor.^41^

So far, the human SUMO proteome comprises more than 6,000 proteins.^11^ The identified SUMOylated proteins are associated with almost all cellular processes, including the main cancer hallmark functions.^3^ The effect of SUMO modification or removal depends on the context of the individual substrate. For example, in the background of HCC, methionine adenosyltransferase α2 (MATα2) is stabilized by SUMOylation and positively controls B cell lymphoma 2 (Bcl-2) expression, enhancing cell survival.^42^ SUMOylation of phosphoenolpyruvate carboxykinase 1 (PCK1) marks the protein for degradation via ubiquitination and helps human hepatoma cells grow by maintaining a glycolytic metabolism.^43^ Along this line, SUMOylation facilitates pyruvate kinase isoform M2 (PKM2) translocation to the plasma membrane of HCC cells and subsequent excretion via ectosomes, which accelerates macrophage differentiation by activating glycolysis and differentiation-associated transcription factors, thereby resulting in the release of cytokines/chemokines and promoting tumor progression.^44^ Liver kinase B1 (LKB1) SUMOylation blocks its nucleocytoplasmic shuttling and favors its oncogenic activity in hypoxic HCC tumors.^45^ Interestingly, polycomb chromobox 4 (Cbx4) SUMO E3 ligase activity controls hypoxia-inducible factor 1α (HIF-1α) SUMOylation to promote angiogenesis in HCC by increasing HIF-1 transactivation and hypoxia-induced vascular endothelial growth factor (VEGF) expression.^46^

Hu antigen R (HuR), also known as HuA and embryonic lethal abnormal vision-like 1 (ELAVL1), is a ubiquitous member of the ELAV/Hu family of RNA-binding proteins (RBPs). By binding through its RNA recognition motifs (RRMs) to U- and AU-rich elements (AREs) typically present in the 3′ untranslated region (UTR) of transcripts, HuR owns the posttranscriptional control of a large number of genes, enabling the protein to play pivotal roles that are dictated by the molecular functions of its target mRNAs.^47,48^ In turn, HuR tumorigenic effect is proposed to result from the function that it exerts on its target transcripts, which contribute to the main cancer traits (i.e., cell proliferation and survival, angiogenesis, evasion of immune recognition, invasion, and metastasis).^49–51^ In the past few years, the relevance of HuR in liver cancer has been extensively reviewed, as multiple signaling pathways implicated in HCC involve the RBPs.^52–55^ For instance, HuR plays a crucial role in hepatocyte proliferation, dedifferentiation, and malignant transformation by promoting the stabilization and expression of *cyclin A2* (*CCNA2*), *CCND1*, and *MAT2A* mRNAs, among others.^56,57^ Also, HuR decreases the translation of the death receptor *FAS* mRNA, shielding HCC cells from FAS-induced apoptosis and immune surveillance.^58,59^ Interestingly, PTMs account for the main mechanism of regulation for HuR function, allowing the protein to elicit quick changes in gene expression programs.^60,61^ Of note, it has been described that HuR protein abundance, subcellular localization, and RNA-binding affinity can be modulated by methylation,^62^ phosphorylation,^63^ proteolytic cleavage,^64^ ubiquitination,^65^ neddylation,^66^ PARylation,^67^ sulfhydration,^68^ and arginylation.^69^

Here, we describe that HuR is SUMOylated at a higher degree in the tumor sections collected from patients with HCC in contrast to the surrounding tissue as well as in human cell line and mouse models of the disease. SUMOylation of HuR promotes major cancer hallmarks, namely proliferation and invasion, whereas the absence of HuR SUMOylation triggers a senescent phenotype with damaged mitochondrial and endoplasmic reticulum (ER) structure and function. Regarding the mechanism of action, SUMOylation induces a structural rearrangement of the RRMs that modulates HuR binding affinity to its target RNAs, further modifying the transcriptomic profile toward hepatic tumor progression. On the one hand, understanding the effects of HuR SUMOylation in hepatocarcinogenesis will provide insights into the relatively unknown role of SUMOylation in cancer. On the other hand, this mechanism of HuR regulation may be potentially exploited as a combination therapeutic strategy for HCC, thus highlighting the importance of PTMs as disease targets.

## RESULTS

### HuR SUMOylation is increased in human HCC

**Figure 1.**
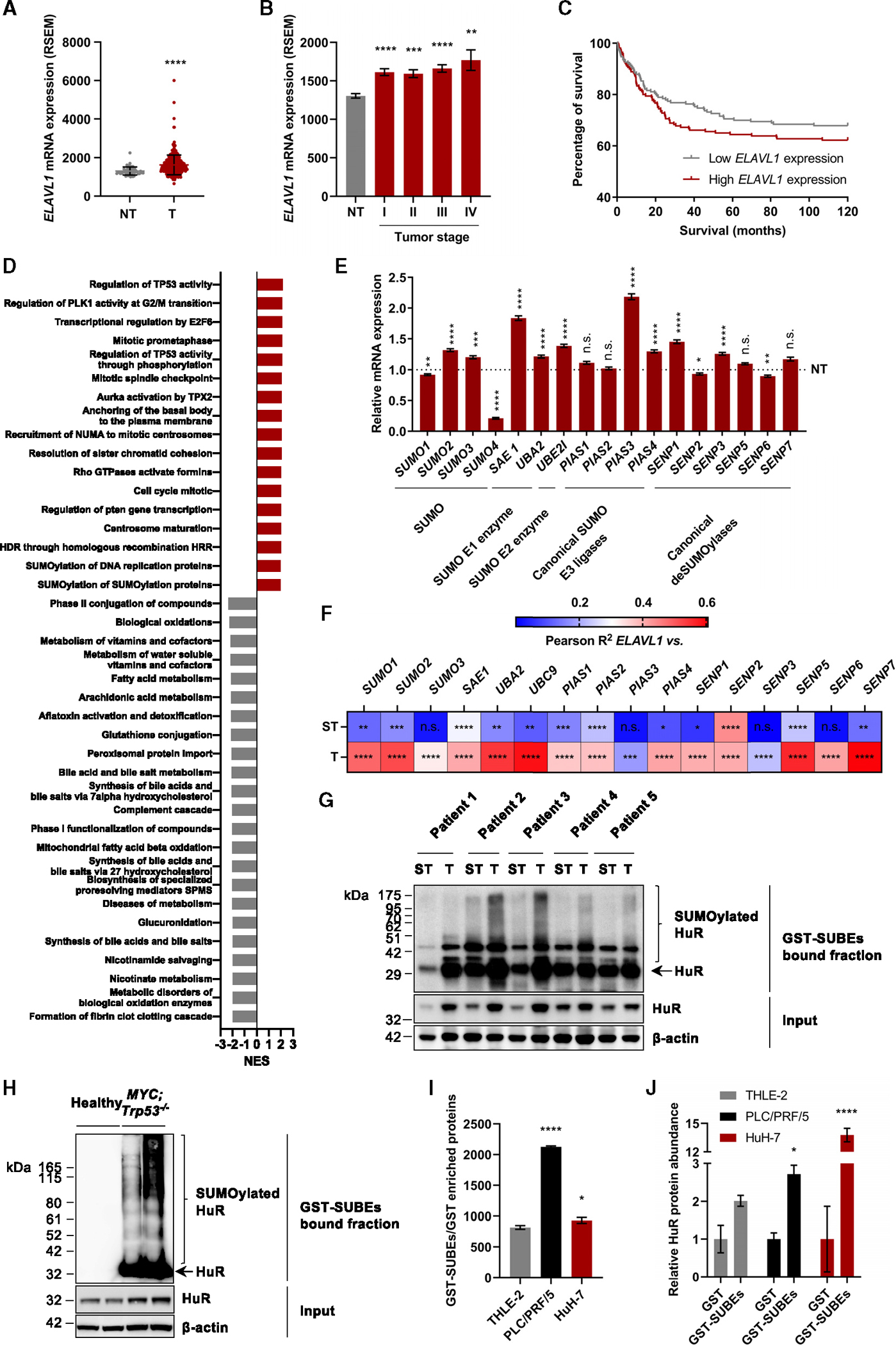
HuR SUMOylation is increased in human HCC (A and B) *ELAVL1* mRNA expression levels (A) in the T (n = 368) and NT (n = 50) tissue of patients with HCC, and (B) at the different stages (I–IV) of the disease. (C) Survival curve of liver cancer patients with high (n = 180) and low (n = 190) *ELAVL1* mRNA expression levels. (D) Enriched molecular processes after performing a GSEA according to *ELAVL1* mRNA expression in HCC patients. (E) mRNA expression levels of the main components of the SUMO pathway in the T (n = 370) of HCC patients relative to the NT tissue (n = 50). (F) Heatmap representing R^2^ values obtained from Pearson correlation studies on *ELAVL1* mRNA expression and the canonical SUMOylation pathway members in paired T and ST liver samples from a cohort of patients with HCC (n = 86). (G–J) Enrichment, identification, and quantification of the SUMO-interacting proteome from non-tumoral and tumoral (G) human (n = 5) and (H) mouse liver tissue (n = 10) and (I and J) human cell line protein extracts by means of GST-SUBEs pull-down technology in combination with (G and H) western blotting and (I and J) LC-MS/MS. Data in (A–E) were obtained from TCGA Research Network. Data in (A), (B), (E), (I), and (J) are presented as the mean ± SD of at least three biological replicates within one representative experiment. *p < 0.05, **p < 0.01, and ***p < 0.001, two-tailed t test vs. NT (A, B, and E), THLE-2 (I), or GST (J). If not indicated otherwise, the differences were not significant. In (H), western blots are representative of at least three biological replicates. See also Figure S1.

Considering the role of HuR in the hepatic malignant transformation, we aimed to evaluate *ELAVL1* mRNA levels in HCC tumors. According to data retrieved from The Cancer Genome Atlas (TCGA) mRNA expression repository,^70^ *ELAVL1* was found significantly upregulated in the tumor (T) tissue of patients with HCC when compared to non-tumor (NT) tissue (Figure 1A). Moreover, high *ELAVL1* mRNA expression applied to all liver cancer stages (Figure 1B) and was associated with poorer patient survival (Figure 1C).

Based on *ELAVL1* mRNA expression levels in patients with HCC, a gene set enrichment analysis (GSEA) was performed, and the molecular pathways showing more than a 2-fold normalized enrichment score (NES) were represented (Figure 1D). It can be seen that *ELAVL1* mRNA expression is mainly associated with cell cycle, TP53 activity and transcription regulation, and protein SUMOylation processes during HCC, while it seems to be inversely related to the oxidative metabolism, synthesis of bile acids and salts, metabolism of vitamins and cofactors, and compound detoxification. Focusing on SUMOylation, a significantly deregulated mRNA expression of the major components of the SUMO cycle was observed in the tumor sections of patients with HCC (Figure 1E). Along this line, Pearson correlation analyses based on an mRNA array obtained from a cohort of 86 patients with HCC revealed a stronger co-variation between *ELAVL1* and the different members of the SUMOylation pathway in the T in contrast to the paired surrounding tissue (ST) (Figures 1F and S1).

Given the evidence suggesting that HuR could be posttranslationally modified by SUMOylation during HCC, a protein pull-down technology based on glutathione S-transferase (GST)-tagged SUMO-binding entities (SUBEs) was used to capture the SUMO-modified proteome both from tissue and cultured cell extracts.^45,71,72^ These tools contain tandem-repeated SIMs from RING-finger 4 (RNF4) SUMO-targeted ubiquitin ligase (STUbL) that specifically recognize polySUMOylated substrates and enable their purification by acting as molecular traps,^71^ thus showing an improved capacity over the originally developed ones.^73^ On the one hand, SUBE-mediated protein pull-down from liver tissue samples of a cohort of patients with HCC (n = 5) in combination with western blotting analysis revealed a significant enrichment of HuR in the T in contrast with the associated ST (Figures 1G and S1A). These results were further validated in the liver tumor tissue of the *MYC*;*Trp53*^−*/*−^ genetically engineered mosaic mouse model of HCC^74^ when compared with healthy control tissue (Figure 1H).

On the other hand, the ratio of SUMO-interacting proteins resulting from the GST-SUBEs relative to the GST-mediated protein pull-down in combination with liquid chromatography-tandem mass spectrometry (LC-MS/MS) in human liver cell lines disclosed a greater number of SUMO-modified proteins in the PLC/PRF/5 and HuH-7 hepatoma cell lines than in the NT THLE-2 cell line (Figure 1I). Approximately 6% of the trapped proteins were shared among the three cell lines, and 12% were exclusively identified in the hepatoma cell lines but not in THLE-2. Furthermore, HuR was found particularly enriched after the GST-SUBEs in contrast to the GST-mediated protein pull-down in the human hepatoma cell lines, especially in the HuH-7 cell line (Figure 1J). Interestingly, mRNA and protein expression data suggest that, in addition to HuR, the SUMOylation machinery might be more induced in the HuH-7 cell line than in PLC/PRF/5 (Figures S1B and S1C), supporting the reason why higher HuR SUMOylation levels are found in the former cell line.

Altogether, we corroborate that *ELAVL1* expression and SUMO dynamics are decompensated in human liver cancer tumors, which led us to describe that HuR is SUMOylated in HCC mouse and human cell line models and, more importantly, in patients.

### Deciphering the SUMOylation machinery and SUMOylatable lysines in HuR

**Figure 2.**
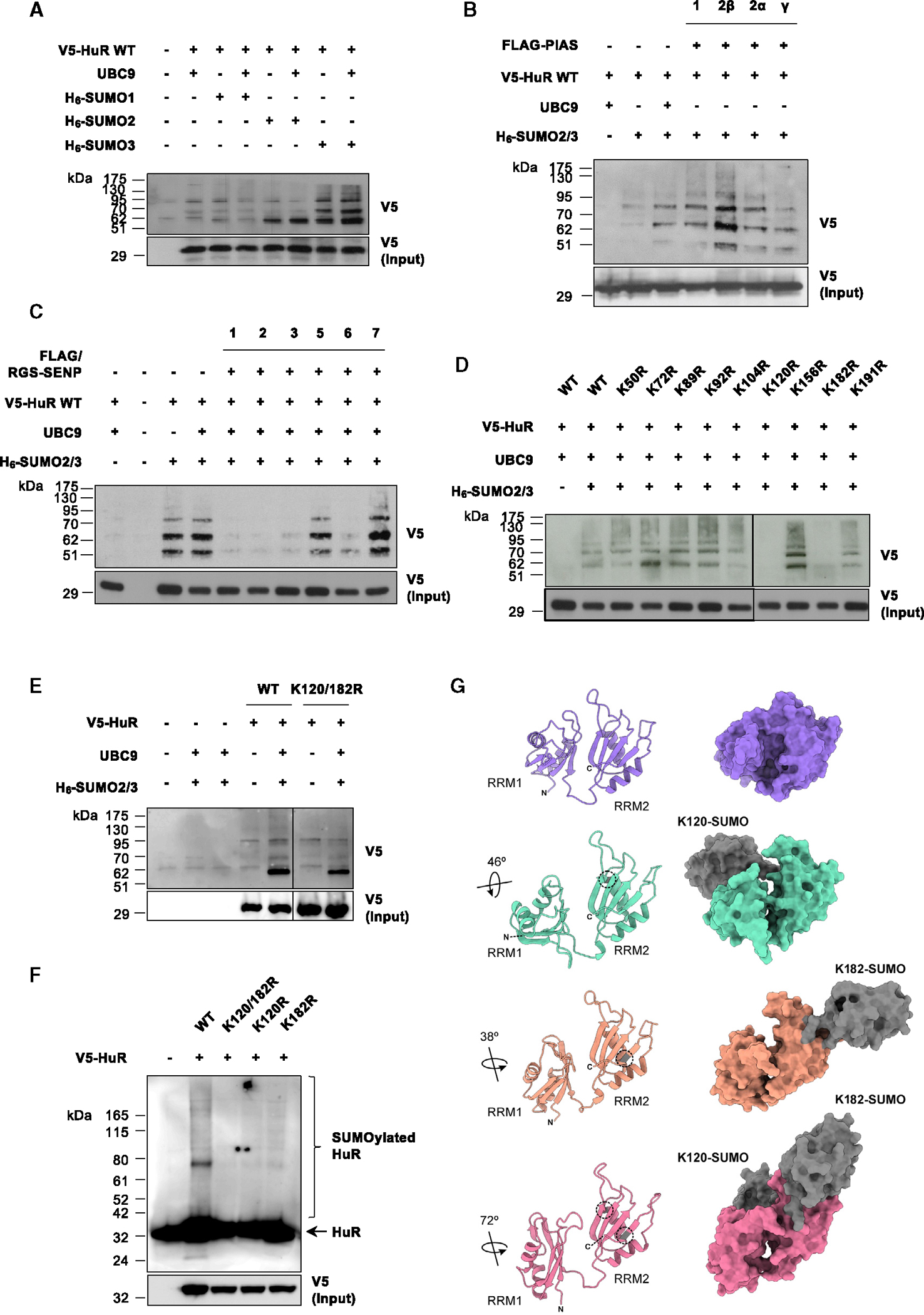
HuR is mainly modified by SUMO2/3 at lysines in position 120 and 182 inducing a structural rearrangement of the RRM 1 and 2 so as to modulate its intrinsic RNA-binding ability (A–D) Modified V5-HuR protein enrichment after transient transfection of plasmids expressing the different (A) SUMO, (B) PIAS, and (C) SENP isoforms as well as (D) all the lysine-to-arginine HuR mutants contained in the RRM1–2 domains, and subsequent nickel-histidine affinity purification, relative to total V5-HuR protein expression levels in the mouse liver progenitor MLP-29 cell line. (E) Modified V5-HuR protein enrichment after co-transfection of plasmids expressing UBC9, SUMO2/3, and WT HuR or the SUMOylation double mutant, and subsequent nickel-histidine affinity purification, relative to total V5-HuR protein expression levels in the human hepatoma HuH-7 cell line. (F) SUMOylated HuR enrichment after transient transfection of WT HuR and the different SUMOylation mutants and subsequent GST-SUBEs protein pull-down, relative to total V5-HuR protein expression levels in the HuH-7 cell line. (G) Ribbon and surface representations of the HuR RRM1–2 protein construct and the different single and doubly SUMOylated species (K120, K182, and K120/182). RRM2 domains were kept in a fixed position in all representations to show them in the same orientation. The rotation angles of RRM1 relative to RRM2 (using the WT conformation as a reference) are depicted on the left of SUMOylated HuR models, and SUMOylated sites are highlighted with dashed circles on the ribbon structures. Models correspond to the structures with the lowest root-mean-square deviation (RMSD) with respect to the last 10-ns average coordinates in each MD trajectory. In (A–F), western blots are representative of at least three biological replicates within one representative experiment. In (D) and (E), the entire blot image was digitally processed to eliminate irrelevant lanes. See also Figures S2–S6 and Table S3.

A molecular characterization of HuR SUMOylation was performed in the MLP-29 cell line by means of transient plasmid transfections to express different His_6_-tagged SUMO constructs, among others, followed by downstream nickel-histidine affinity purification and western blotting analysis of V5-tagged HuR. First, SUMOylated HuR levels were found particularly increased upon SUMO2/3 overexpression, which suggested that this could be the main HuR SUMO modifier (Figures 2A and S2A). Next, canonical SUMO E3 ligases and deSUMOylating enzyme plasmids were transfected together with wild-type (WT) HuR, UBC9, and SUMO2/3. As shown, PIAS 2β overexpression resulted in an increased V5-tagged HuR protein enrichment (Figures 2B and S2B), while SENP1, 2, and 3 overexpression caused a decrease in V5-HuR detection (Figures 2C and S2C) and were therefore designated as the main enzymes implicated in HuR SUMOylation and deSUMOylation processes, respectively. Likewise, small interfering RNA (siRNA)-mediated downregulation of *Pias2b* (Figure S3A) almost abolished the smear of V5-tagged HuR protein expression corresponding to its modified state (Figure S3B), whereas knockdown of *Senp1*, *2*, and *3* (Figure S3A) resulted in an increased pattern of modified V5-HuR protein expression (Figure S3C) in the MLP-29 cell line transiently expressing WT HuR, UBC9, and SUMO2/3, reinforcing the findings obtained in the overexpression studies. Along this line, HuR SUMOylation status assessed through nickel-histidine affinity purification also led to a significant enrichment of V5-tagged HuR protein levels in the MLP-29 cell line transiently co-transfected with plasmids inducing the expression of WT HuR, UBC9, and SUMO2/3, in addition to *Senp1* or *Senp2* siRNAs (Figure S3D), further validating the enzymatic machinery regulating HuR deSUMOylation.

Regarding the characterization of HuR SUMOylation in the HuH-7 human hepatoma cell line, V5-HuR was found to be particularly enriched after His_6_-tagged SUMO2 transient expression and downstream nickel-histidine affinity purification (Figure S4A). Since SUMO2 and SUMO3 share ~95% sequence identity, they are usually referred to as the SUMO2/3 subfamily.^8^ Hence, SUMO2/3 is likely to be the main SUMO form attaching to HuR not only in the MLP-29 but also in the HuH-7 cell line. Regarding the profiling of canonical SUMO E3 ligases and deconjugating enzymes in the HuH-7 cell line, HuR protein expression and its smear, which is intended to reflect its modified state, were evaluated upon transient transfection of PIAS and SENP plasmids. Thus, HuR smear was increased when PIAS 2β expression was induced (Figure S4B), as it occurred in the MLP-29 cell line (Figure 2B). Conversely, HuR smear might decrease after induction of SENP6 expression but also in the presence of SENP1, 2, and 3 (Figure S4C) and could resemble the results obtained in the case of the MLP-29 cell line (Figure 2C), despite the evident differences between the two cell lines.

According to several SUMOylation site prediction tools,^75–77^ HuR protein sequence does not contain any forward ψ-K-X-E/D or inverted E/D-X-K (ψ is a large hydrophobic residue and X is any amino acid) SUMO consensus motif.^12,78^ However, a considerable proportion of experimentally validated SUMOylation sites do not match any of these motifs.^79,80^ Consequently, in order to define those, HuR SUMOylation mutant plasmids were designed by modifying all lysine residues located in the RRM1 and 2 primary sequences into arginine by means of site-directed mutagenesis and were co-transfected with the UBC9 SUMO E2-conjugating enzyme and SUMO2/3 in the MLP-29 cell line. The lack of V5-HuR protein enrichment observed when mutating the lysine residues at positions 120 and 182 revealed that HuR SUMOylation could be occurring at one or both of those sites (Figures 2D and S4D), leading to the creation of the HuR K120/182R SUMOylation double-mutant construct. HuR SUMOylation sites were further confirmed in the human hepatoma HuH-7 cell line (Figures 2E and S4E). Also, considering that SUBEs preferentially retain substrates comprising polySUMO2/3 chains in non-denaturing conditions and without the need of additional transfections,^71^ we were able to corroborate the loss of SUMOylation of the K120/182R HuR mutant relative to WT HuR in the HuH-7 cell line by using this protein pull-down technology (Figure 2F). Additionally, HuR SUMOylation was studied in the context of hypoxia, a hallmark of many solid tumors showing an aggressive phenotype,^81^ in addition to a well-known inductor of protein SUMOylation.^82^ In this case, the HuH-7 human hepatoma cell line was transiently transfected with the constructs expressing V5-tagged WT or K120/182R HuR and incubated under hypoxic conditions (1% O_2_) for 24 h. Immunoprecipitation of the different HuR variants using anti-V5 antibody followed by western blotting analysis revealed that hypoxia is an enhancer of HuR SUMOylation, while the lower SUMO2/3 protein enrichment levels of the mutant variant corroborate that Lys120 and 182 constitute HuR SUMOylation residues in the HuH-7 cell line (Figure S5A).

Importantly, to verify that Lys120 and 182 are not ubiquitination sites, the HuH-7 cell line was transiently transfected with plasmid vectors inducing the expression of His_6_-tagged ubiquitin in addition to V5-tagged WT HuR and the different SUMOylation mutant variants. Protein extracts were submitted to nickel-histidine affinity purification and western blotting analysis of V5-tagged HuR, which revealed that the K120R, K182R, and K120/182R HuR mutants exhibited ubiquitination, discarding these positions as major ubiquitination sites while reinforcing their relevance as SUMOylation residues (Figure S5B). Along this line, a dose of 100 nM ML-792 SAE inhibitor for 4 h did not only lead to a significant decrease in global protein SUMOylation (Figure S5C) but also resulted in a reduction of HuR SUMOylation (Figure S5D), assessed by means of transient transfection of plasmids inducing the expression of HuR, UBC9, and SUMO2/3 in the HuH-7 cell line followed by downstream nickel-histidine affinity purification and western blotting analysis. Hence, these findings provide proof of concept that HuR is SUMOylated in this hepatoma cell line. Finally, to gain further insight into the interplay between SUMOylation and ubiquitination, protein extracts from the HuH-7 cell line transiently transfected with His_6_-tagged ubiquitin and WT and K120/182R HuR in addition to treatment with 100 nM ML-792 for 4 h were subjected to nickel-histidine affinity purification. Western blotting analysis of V5-tagged HuR revealed a significant reduction in WT HuR ubiquitination in the presence of the SAE inhibitor, whereas the ubiquitination levels of the K120/192R HuR variant remained mostly unaltered (Figure S5E). These results suggest that HuR ubiquitination might occur as a SUMOylation-dependent process in the HuH-7 human hepatoma cell line.

Overall, HuR SUMOylation consists in the covalent addition of SUMO2/3 subunits to lysine residues located at positions 120 and 182. This process is likely to be catalyzed by PIAS 2β SUMO E3 ligase and reversed by the action of SENP1, 2, and 3 deSUMOylating enzymes.

### SUMOylation induces a structural rearrangement of RRM1 and 2 in HuR, modulating its intrinsic RNA-binding ability

The RRMs are crucial for HuR to accomplish its RNA-binding function and, consequently, its oncogenic activity.^48^ Therefore, in addition to describing the molecular elements implicated in HuR SUMOylation and its reverse reaction, putative models of the structural and dynamic changes of the HuR RRM1–2 tandem including the SUMO molecules were assessed by molecular dynamics (MD) simulations. In the available crystal structure of the HuR RRM1–2 used as a model (PDB: 4ED5),^83^ which contains a short oligonucleotide derived from the *c-fos* mRNA, the two domains face each other with a 172° angle, leaving a basic cleft for RNA binding (Figure 2G). The RNA chain was removed from the starting structure in the first series of MDs described below. Likewise, along the computations in the absence of SUMO, the β sheets containing the corresponding RNA-binding motifs also face one to the other in a V-shape arrangement. In the average structure obtained from MD, this basic cleft opens with an angle of approximately 90°, which represents a small rigid-body motion of both HuR domains with the flexible linker acting as a hinge, as inferred from the trajectory analysis (Figure S6A). Furthermore, the reorientation of the two RRM domains lacking SUMO induces a small compaction of the construct.

However, SUMOylation at Lys120 produces a 46° turn of RRM1 relative to RRM2, almost parallel to the longitudinal axis of the HuR model, which becomes stretched. The respective β sheets no longer face each other but lie almost perpendicular (Figure 2G). In contrast, when a SUMO molecule is linked to Lys182, the angle between the two β sheets is 137°. Under these circumstances, the two domains separate and the linker extends. Besides slightly lengthening the HuR moiety (for *R*_G_ values see Figure S6A), RRM1 turns 38° with respect to RRM2 in a transverse plane, yielding a new orientation of the RRM1–2 β sheets that causes the closure of the cleft (Figure 2G), which conceals most of the residues involved in RNA recognition (Figure S6B). Moreover, when concurrent SUMOylation of Lys120 and Lys182 is simulated, the turn of RRM1 regarding RRM2 is more pronounced (72°) than that originated when only the Lys182 is modified, although the orientation of the β sheets remains similar (Figure 2G). Thus, a lower affinity of HuR toward RNA can result from either or both RRM misorientation and concealment of key target residues.

Hence, to investigate the possible impact of the two Lys-to-Arg mutations (K120R and K182R) and its combination on the ability of HuR RRM1–2 to bind RNA, *in silico* predictions validated with *in vitro* experiments were provided. Three new 80-ns MD trajectories were computed using the abovementioned X-ray diffraction (XRD) coordinates (PDB: 4ED5)^83^ but this time maintaining the RNA molecule (Figure S6C). No remarkable changes in the behavior of the HuR:RNA complexes were observed along the trajectories. According to the MD analyses, these SUMOylation-disrupting mutations exert hardly any influence on the conformation of the complexes once they are formed.

Regarding the *in vitro* validation of *in silico* data, isothermal titration calorimetry (ITC) assays were performed using HuR RRM1–2 WT and SUMOylation mutant constructs and an 11-mer T-rich single-stranded DNA (ssDNA) analog, derived from an ARE of the *c-fos* mRNA.^83^ Previously, all HuR RRM1–2 constructs used in the ITC assays, namely, WT, K120R, K182R, and K120/182R, were previously subjected to a thorough quality control of purity, folding, and homogeneity assessed by SDS-PAGE, circular dichroism (CD), and dynamic light scattering (DLS), respectively (Figures S6D and S6F). No significant differences were observed in any of the characteristics of the HuR RRM1–2 samples tested. As for the ITC results, none of the mutations, either alone or in combination, appreciably alter the dissociation constant (K_D_), stoichiometry, or thermodynamic parameters for the association of HuR RRM1–2 species with the ssDNA (Figure S6G and Table S3). The Lys120 residue is located in the helix α_1_ of RRM2, on the opposite side to the RNA-binding surface of this domain. Therefore, its contribution to the interaction of HuR with nucleic acids would presumably be nil. On the other hand, the Lys182 residue is found in the sheet β_4_, also in RRM2, and can establish a hydrogen bond with a keto group of uracil through the amino group of its side chain.^83^ According to our ITC data, single lysine-to-arginine substitution at Lys182 does not substantially modify the affinity of HuR for nucleic acids either, regardless of K120R mutation.

Additionally, Brownian dynamics (BD) simulations using the HuR RRM1–2 WT and K182R constructs in complex with a 10-mer RNA oligo showed that, at the contact level, calculated association rates (k_on_) and residence (dwelling) times for WT are similar to those estimated for the K182R mutant (Figure S6H). This fully agrees with the absence of any significant difference in K_D_ values as monitored by ITC and further indicates that binding and dissociation kinetics are also analogous.

Protein binding to DNA/RNA molecules relies on multiple interactions of various types, ranging from the individually weak van der Waals forces to the relatively strong π-π stacking of aromatic rings.^84^ Many RBPs such as HuR contain a series of key residues that define the spectrum of nucleic acids with which they can interact and modulate the affinity for their partners. However, the structural flexibility of mRNAs (or ssDNA in our *in vitro* model) allows them to accommodate the minor change in the interaction surface of HuR caused by the subtle K182R mutation. Arginine is also a hydrogen-bonding donor, chemically similar to lysine, so a slight rearrangement of the DNA/RNA targets would be enough to continue binding to HuR with the same affinity. Taken together, experimental evidence indicated that HuR RRM1–2 SUMOylation site mutants display the same nucleic acid binding affinity as the non-SUMOylated WT construct *in vitro*, supporting their use in cellular assays to evaluate the effects of HuR SUMOylation.

Structurally, the RRM1–2 tandem adopts a substantially less compact arrangement when HuR is SUMOylated in positions 120 and 182, suggesting that SUMOylation may critically alter HuR conformation so as to modulate its intrinsic RNA-binding ability during liver cancer.

### SUMOylation of HuR promotes the main cancer hallmarks in human hepatoma cells

To study the tumorigenic advantage of HuR SUMO modification, human hepatoma HuH-7 cells stably expressing WT HuR and the described K120R, K182R, and K120/182R SUMOylation mutant plasmids were generated by means of transfections and antibiotic selection (Figures S7A and S7B). Ectopic protein expression of WT and mutant HuR was slightly altered in the different HuH-7 cell variants (Figure S7A) even though global HuR protein levels, which also include endogenous HuR, were not dramatically affected (Figure S7B). Nevertheless, ectopic HuR protein expression remained comparable between the WT and K120/182R SUMOylation mutant (Figure S7A).

**Figure 3.**
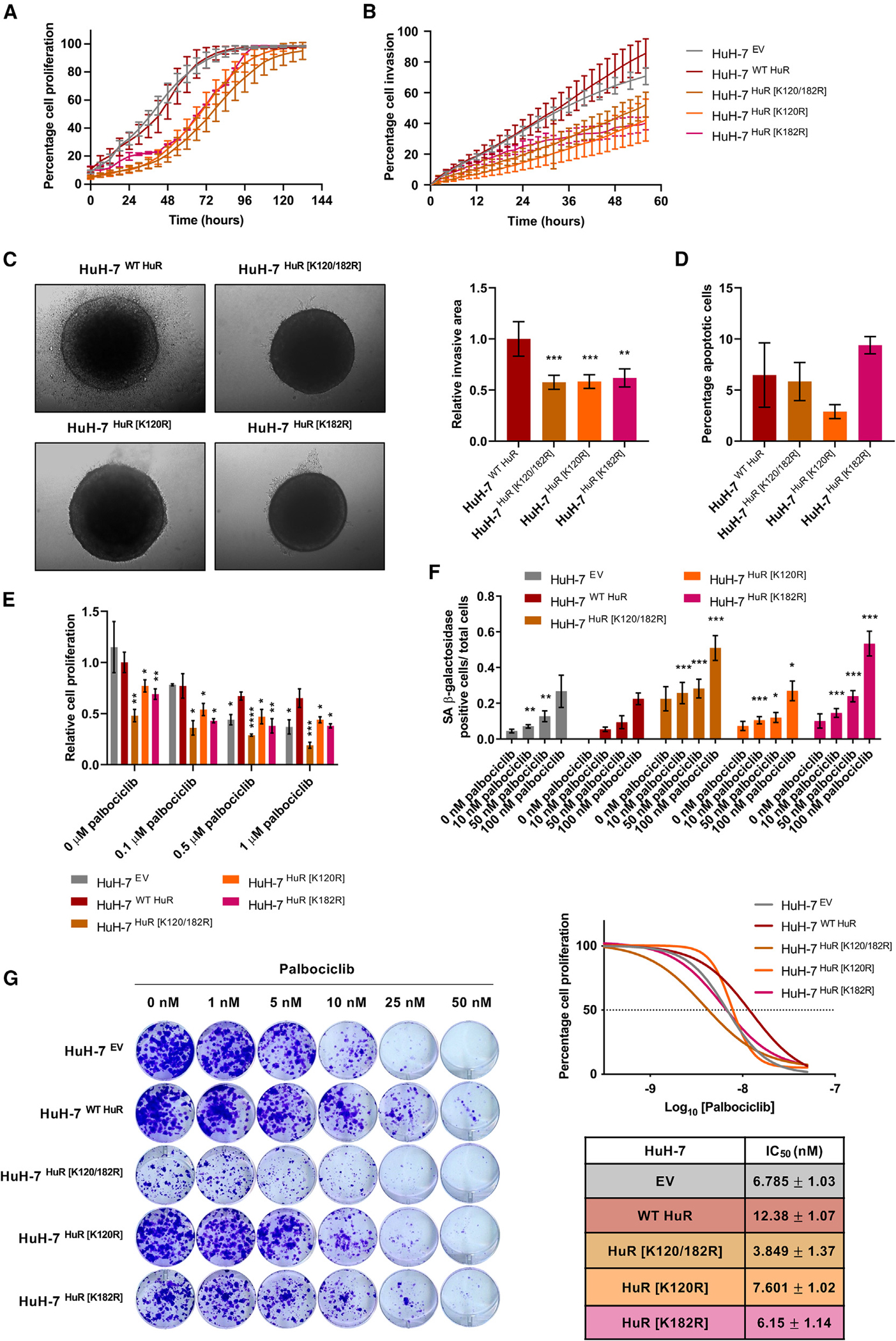
SUMOylation of HuR promotes the main cancer hallmarks and avoids palbociclib-induced senescence in human hepatoma cells (A and B) Cell (A) proliferation and (B) scratch-wound healing process of HuH-7 cell lines stably expressing WT HuR and the different SUMOylation mutants analyzed in the IncuCyte system. (C) Representative pictures of 3D spheroids from HuH-7 cell lines stably expressing WT HuR and the SUMOylation mutant species embedded on a collagen type I matrix for 48 h, and quantification of the relative invasive area. (D) Percentage of apoptosis detected in the WT HuR and the indicated SUMOylation mutant HuH-7 cell variants by flow-cytometry analysis after FITC-annexin V and viability stainings, relative to STS-treated HuH-7 cells. (E) Quantification of cell proliferation in the HuH-7 cell lines stably expressing WT HuR and the different SUMOylation mutants after an acute 3-day treatment with a range of palbociclib concentrations analyzed by crystal violet staining. (F) Quantification of relative senescence in the HuH-7 cell lines stably expressing the WT and the K120/182R, K120R, K182R HuR mutant species after chronic 2-week treatment with a range of palbociclib concentrations analyzed by β-galactosidase staining. (G) Crystal violet staining of colonies from HuH-7 cell lines stably expressing WT HuR and the SUMOylation mutants treated with the indicated doses of palbociclib for 10 days, and generation of dose-response curves for calculation of IC_50_ values. In (A–F), data are presented as the mean ± SD of at least three biological replicates within one representative experiment. *p < 0.05, **p < 0.01, and ***p < 0.001, two-tailed t test vs. HuH-7^WT HuR^. If not indicated otherwise, the differences were not significant. In (C) and (G), images are representative of at least three biological replicates within one representative experiment. See also Figures S7–S9.

Live-cell analysis of proliferation in an IncuCyte system showed that cells expressing WT HuR grew faster than those where HuR SUMOylation was hampered (Figures 3A and S8A). Interestingly, the differences between WT HuR and the K120/182R SUMO double mutant expressing cells were particularly evident. The cell invasion potential was assessed in a wound-healing assay also by using the IncuCyte system, which showed that SUMOylated HuR promotes a more invasive phenotype than in the absence of SUMOylation (Figures 3B and S8B). These data were further corroborated in a 3D invasion assay based on HuH-7 cell spheroid culture on a type I collagen matrix (Figures 3C and 3D). To discard that any apoptotic event was occurring, analysis of annexin V-fluorescein isothiocyanate (FITC) by flow cytometry resulted in an insignificant staining for any HuH-7 cell variant when compared to staurosporine (STS)-treated cells, which were used as a positive control (Figure 3D).

Collectively, HuR SUMOylation stimulates human HCC cell proliferation and invasion, whereas the lack of HuR SUMOylation results in suppressed cell growth, in the absence of apoptosis.

### HuR SUMOylation avoids palbociclib-induced senescence response in human hepatoma cells

Palbociclib (PD-0332991, Ibrance; Pfizer) is a potent and selective inhibitor of cyclin-dependent kinase 4 (CDK4) and highly homologous CDK6 approved by the US Food and Drug Administration (FDA) for the treatment of breast cancer.^85^ It is predicted to exclusively have a good outcome in tumors where retinoblastoma (RB1) function remains intact, as it occurs in roughly 70% of patients with HCC.^86,87^ There is an ongoing phase 2 clinical trial evaluating palbociclib for patients with advanced HCC who are non-responders to sorafenib, which led us to study its effect in combination with the inhibition of HuR SUMOylation.

A 3-day treatment with a range of palbociclib concentrations resulted in attenuated HuH-7 cell-line proliferation, assessed by crystal violet staining (Figures 3E and S9A). A dose of 1 μM palbociclib was enough to inhibit cell growth by nearly 50%. Interestingly, the differences in cell proliferation observed among the HuR SUMOylation mutants in basal conditions were intensified after palbociclib treatment. A caspase-3 activity assay performed in parallel with an STS-positive control confirmed that palbociclib does not act by inducing apoptosis in the HuH-7 cells and neither were significant changes between the different HuR SUMOylation mutants detected (Figure S9B). Conversely, palbociclib is known to activate cell senescence, as observed by senescence-associated β-galactosidase (SA β-gal) activity staining after a prolonged 2-week exposure to palbociclib in the low-nanomolar range of concentrations (Figures 3F and S9C). The proportion of senescent cells correlated with palbociclib concentration, but more importantly the percentage of SA β-gal-positive cells, was significantly greater in the case of the K120/182R HuR mutant than in the rest of the HuH-7 cell variants at every drug concentration. In other words, palbociclib induces human hepatoma cell growth arrest, especially under conditions where HuR is not SUMOylated.

Likewise, a palbociclib dose-response curve generated after a 10-day colony-formation assay and crystal violet staining revealed different half-maximal inhibitory concentration (IC_50_) values for each of the cell variants (Figure 3G). Higher IC_50_ values were obtained for HuH-7 cells expressing WT HuR than for those expressing HuR SUMOylation mutants. In particular, the K120/182R HuR variant showed the lowest IC_50_ value (3.85 ± 1.37 nM), suggesting that SUMOylation of HuR confers cells resistance to palbociclib-induced senescence, while the absence of SUMOylation increases their sensitivity to the drug. Considering the potency of the drug, 5 nM palbociclib was the dose used for chronic treatments since, at this concentration, the HuR K120/182R mutant showed a senescence-like phenotype while the WT HuR expressing HuH-7 cells remained unaffected.

CDK4 and CDK6 play a key role in mammalian cell proliferation by working in complex with CCND1 to phosphorylate and inhibit RB1 tumor-suppressor activity during early G_1_ phase. Regarding palbociclib’s cytostatic mechanism of action, a decrease in RB1 phosphorylation at Ser780 and accumulation of CCND1 were confirmed as a consequence of CDK4/6 inhibition and stabilization of an inactive CCND1-CDK4 complex, respectively, in addition to an attenuation of CCNA2 protein expression levels (Figure S9D), as previously described.^86,88^ More importantly, however, the suppression of HuR SUMOylation at Lys120 and Lys182 resulted in a pattern of expression of the G_1_-to-S phase-transition proteins similar to that obtained in HuH-7 cells expressing WT HuR after exposure to 5 nM palbociclib for 2 weeks. Again, the latter finding supports the notion that the HuR K120/182R mutant shows a more senescent phenotype than the WT HuR expressing HuH-7 cells and is more susceptible to palbociclib-induced G_1_ arrest. Apart from cell-cycle withdrawal, the senescent phenotype encompasses other hallmarks, including macromolecular damage.^89^ Hence, the phosphorylation of histone H2AX at Ser139 in response to DNA damage was significantly increased in the absence of HuR SUMOylation and enhanced after chronic incubation with palbociclib.

In brief, the ablation of HuR SUMOylation in the human HCC HuH-7 cell line has an additive effect specifically over palbociclib-mediated senescence induction.

### SUMOylated HuR evades palbociclib-mediated senescence by increasing HuR and global SUMOylation levels in human hepatoma cells

**Figure 4.**
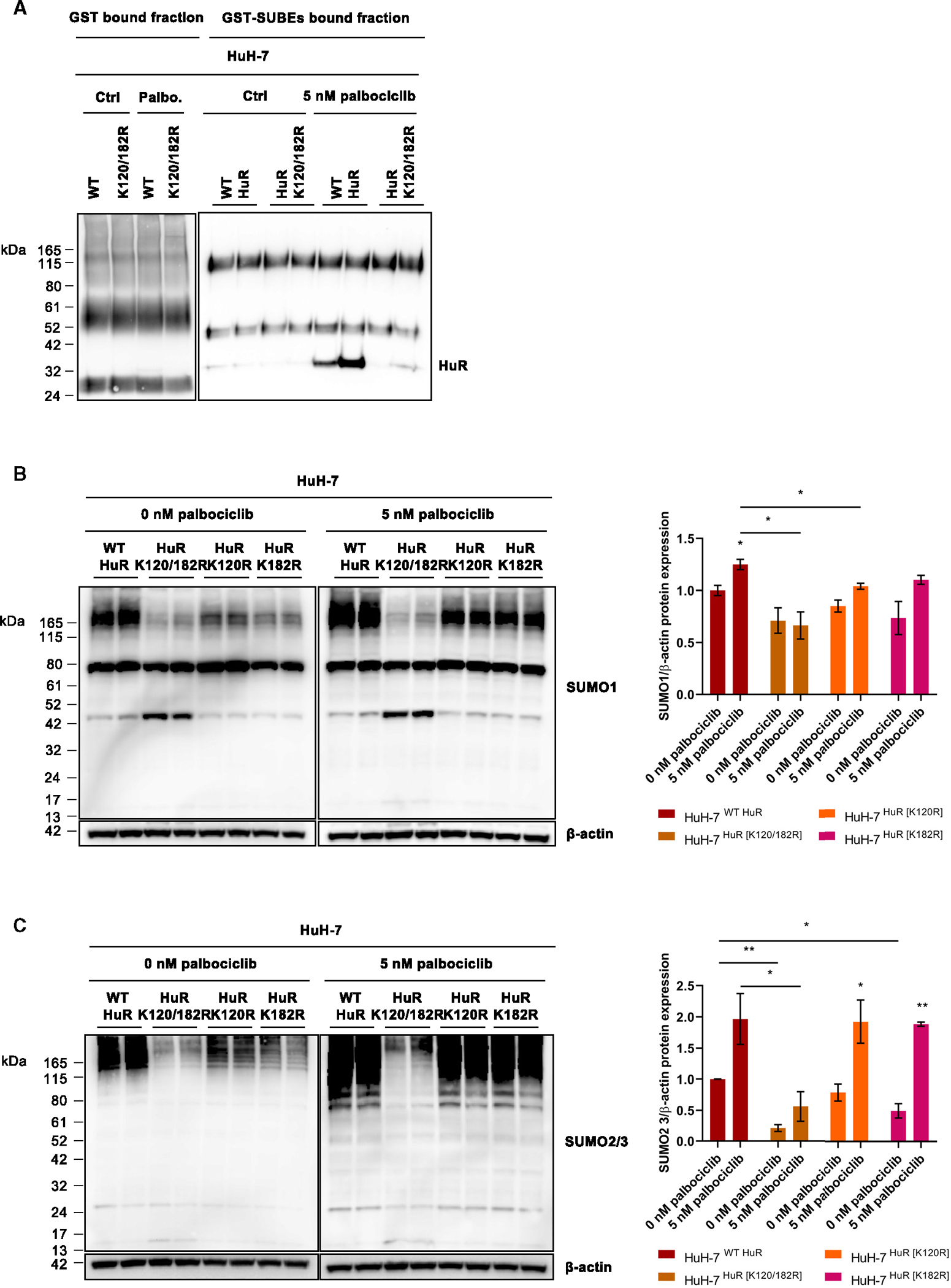
SUMOylated HuR evades palbociclib-mediated senescence by increasing HuR and global SUMOylation levels in human hepatoma cells (A) Enrichment of SUMOylated HuR in the HuH-7^WT HuR^ and HuH-7^HuR [K120/182R]^ cells treated with 5 nM palbociclib for 2 weeks by means of protein pull-down with GST control and SUBEs in combination with western blotting analysis. (B and C) (B) SUMO1 and (C) SUMO2/3 protein expression levels and quantification in the HuH-7cell lines stably expressing WT HuR and the K120/182R, K120R, K182R HuR SUMOylation mutant species treated with 5 nM palbociclib for 2 weeks. In (A–C), western blots are representative of at least three biological replicates within one representative experiment. In (B) and (C), data are presented as the mean ± SD of at least three biological replicates within one representative experiment. *p < 0.05 and **p < 0.01, two-tailed t test vs. 0 nM palbociclib or HuH-7^WT HuR^. If not indicated otherwise, the differences were not significant.

To further investigate the mechanism by which SUMOylated HuR protects tumoral cells from entering senescence, SUMOylation levels were evaluated in the HuH-7 cell line after a 5-nM palbociclib exposure for 2 weeks. On the one hand, results obtained by SUBE-mediated protein pull-down confirmed the absence of SUMOylation in the K120/182R HuR mutant, in addition to the enrichment of SUMOylated HuR after palbociclib treatment in the WT HuR expressing HuH-7 cell-line variant (Figure 4A). On the other hand, a significant reduction in global SUMO1 and 2/3 protein expression was found in the HuR SUMOylation mutants in comparison with the WT HuR expressing HuH-7 cells (Figures 4B and 4C). Interestingly, chronic treatment with 5 nM palbociclib resulted in generally increased SUMO1- and 2/3-conjugated protein levels except for the cells expressing the K120/182R HuR mutant, where no SUMOylated HuR is expected and global SUMOylation levels remained downregulated despite senescence induction.

In short, palbociclib increases global levels of SUMOylated proteins, including HuR itself, in all the different HuH-7 cell variants except for the K120/182R HuR mutant. Thus, these data introduce SUMOylated HuR as a modulator of the senescence response, possibly by governing the entire protein SUMOylation process.

### The absence of HuR SUMOylation generates a senescent phenotype with compromised mitochondria and ER in human hepatoma cells

Mitochondria and senescence biology are mechanistically linked. Mitochondria can behave both as downstream effectors or upstream initiators of senescence. It is known that senescent cells exhibit changes in mitochondrial function, dynamics, and morphology.^90^ Therefore, the structure and function of this organelle were examined in the HuH-7 cell line stably expressing WT HuR and the K120/182R SUMOylation mutant variant.

**Figure 5.**
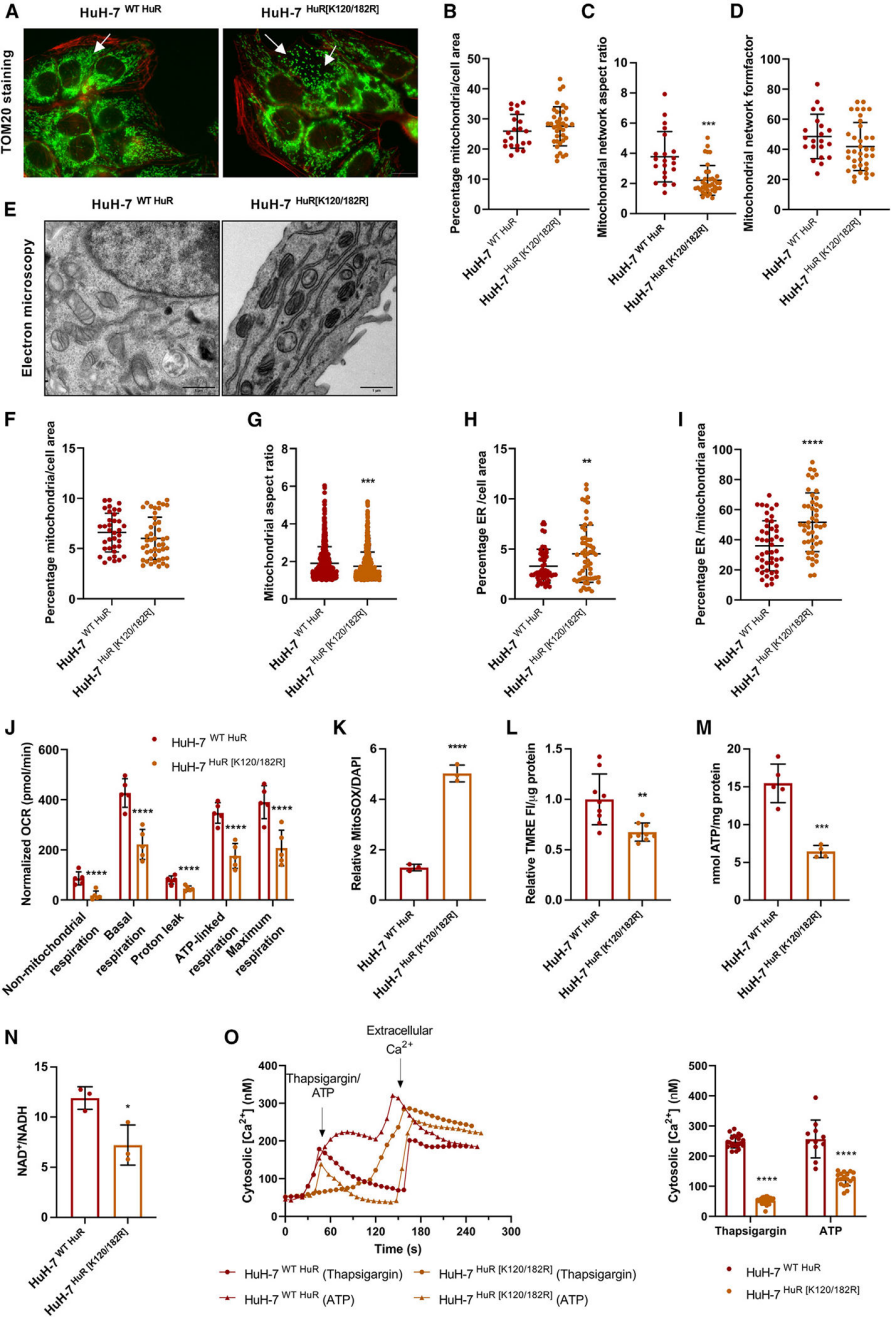
Absence of HuR SUMOylation generates a senescent phenotype with compromised mitochondria and ER in human hepatoma cells (A–D) (A) Representative images of TOM20 immunofluorescent detection and estimation of the (B) mitochondrial content and mitochondrial network (C) AR and (D) F in HuH-7^WT HuR^ and HuH-7^HuR [K120/182R]^ cells. (E–I) (E) Representative TEM images of the mitochondrial and ER ultrastructure and determination of the (F) mitochondrial area relative to cell area, (G) mitochondrial AR, (H) ER area relative to cell area, and (I) ER area relative to mitochondrial area in HuH-7^WT HuR^ and HuH-7^HuR [K120/182R]^ cells. (J) Seahorse-based quantification of mitochondrial respiration parameters in the HuH-7^WT HuR^ and HuH-7^HuR [K120/182R]^ cells. (K–N) Quantification of (K) MitoSOX red mitochondrial superoxide indicator, (L) TMRE staining for the assessment of mitochondrial membrane potential, (M) total ATP content, and (N) NAD^+^/NADH levels in the HuH-7^WT HuR^ and HuH-7^HuR [K120/182R]^ cells. (O) Representative curves and quantification of cytosolic Ca^2+^ levels with Fura-2AM fluorescent probe labeling in the HuH-7^WT HuR^ and HuH-7^HuR [K120/182R]^ cells after stimulating Ca^2+^ release from the ER with thapsigargin and ATP, and addition of extracellular Ca^2+^. In (A) and (E), images are representative of at least three biological replicates within one representative experiment. Scale bars represent 5 μm in (A) and 1 μm in (E). In (B–D) and (F–O), data are presented as the mean ± SD of at least three biological replicates within one representative experiment. *p < 0.05, **p < 0.01, ***p < 0.001, and ****p < 0.0001, two-tailed t test. If not indicated otherwise, the differences were not significant. See also Figure S10.

First, mitochondrial import receptor subunit TOM20 homolog immunofluorescent detection (Figure 5A) revealed that the mitochondrial network covered around 25% of the cell with the z projection in the HuH-7 cells expressing WT and K120/182R HuR (Figure 5B). Although there are no significant differences in the mitochondrial network relative to the total cell area (Figure 5B), MitoTracker green FM fluorescence intensity measured by flow cytometry revealed that a 3-day incubation with 1 μM palbociclib senescence inductor seems to increase the mitochondrial mass to a greater extent in the K120/182R HuR mutant HuH-7 cells (Figure S10A). Moreover, TOM20 immunostaining enabled the quantification of other mitochondrial network shape descriptors. On the one hand, the aspect ratio (AR), which is the relationship between the major and minor axis of the ellipse equivalent to the mitochondrial network,^91^ was greater than 1 in both cell variants, suggesting an elongated rather than round mitochondrial network (Figure 5C). Besides this, the AR was 2-fold lower for the K120/182R HuR SUMOylation mutant, which indicated a rounder mitochondrial network than in the case of WT HuR HuH-7 cell lines. On the other hand, the form factor (F), which not only reports changes in length but also in the degree of branching,^91^ was greater than 1, thus implying a more branched mitochondrial network (Figure 5D). Importantly, TOM20 staining showed a more fragmented mitochondrial network in the HuR SUMOylation double mutant than in the WT HuR HuH-7 cells (Figure 5A), which might have an impact on mitochondrial function.

In parallel, evaluation of the mitochondrial ultrastructure by transmission electron microscopy (TEM) of ultrathin plastic sections (Figures 5E and S10B) reported no significant changes in the cellular mitochondrial area (Figure 5F) but again suggested a rounder organelle morphology in the case of the HuH-7 cells expressing the K120/182R HuR SUMOylation mutant (Figure 5G). Although often viewed as autonomous structures, there is growing appreciation that an important communication exists between mitochondria and the ER.^90^ Thus, differences in the number of ER sheets were addressed, resulting in a greater ER network in the absence of HuR SUMOylation (Figure 5H). Notably, the ER-to-mitochondrial-area ratio was augmented in the HuH-7 cells expressing the HuR SUMOylation mutant compared to the WT version (Figure 5I), making more evident the enlargement of the ER.

Additionally, while mitochondria are reported to be more abundant in senescent cells, they appear to be less functional, showing decreased respiration, ATP production, membrane potential, and increased reactive oxygen species (ROS) production.^89^ Seahorse-based functional studies revealed that HuH-7 cells expressing WT HuR had higher mitochondrial respiration levels than the K120/182R HuR mutant HuH-7 cells (Figures 5J and S10C). These results were corroborated by incubating cells with 1 μM palbociclib for 3 days, which led to a generally decreased mitochondrial respiration especially in the cells expressing K120/182R HuR (Figure S10D).

Respiration studies were accompanied by an increase in mitochondrial superoxide production in the case of the HuR SUMOylation double mutant expressing HuH-7 cells as per fluorescence detection of MitoSOX red staining (Figures 5K and S10E). Measurement of mitochondrial membrane potential with tetramethylrhodamine ethyl ester (TMRE) (Figure 5L) showed a decreased accumulation of the cationic dye in the HuH-7 cells expressing the K120/182R HuR mutant, suggesting more depolarized or inactive mitochondria. Along this line, intracellular ATP concentration was higher in the WT HuR expressing HuH-7 cells than in those where HuR is not SUMOylated (Figure 5M). Upon senescence induction, WT HuR expressing cells became more defective in ATP synthesis, while the K120/182R HuR mutant remained unaffected by palbociclib (Figure S10F). In addition to a decreased ATP production, the NAD^+^/NADH ratio was lower in the HuR SUMOylation double mutant expressing HuH-7 cells (Figure 5N), which is consistent with suppressed energy generation, typical among cells that undergo mitochondrial dysfunction-associated senescence.

Excessive ROS production by mitochondria can target other cellular structures, including the ER. Therefore, cytosolic FURA-Ca^2+^ levels were measured as a readout of ER functionality, since this organelle can closely interact with mitochondria and facilitate the uptake of the cation into the mitochondrial matrix, thus sustaining multiple aspects of its function.^90^ Here, the lack of HuR SUMOylation hampered Ca^2+^ release from the ER triggered by thapsigargin or ATP, indicating a more damaged organelle than in the case of the HuH-7 cells expressing WT HuR (Figure 5O). Furthermore, palbociclib seemed to aggravate ER stress in a way that Ca^2+^ release into the cytosol became hardly detectable (Figure S10G).

On the whole, the lack of HuR SUMOylation results in mitochondrial and ER dysfunction-associated senescence, in a similar way to palbociclib-induced senescence. In the absence of HuR SUMOylation, HuH-7 cells respond by adopting a more spherical morphology and increasing the ER mass. However, the resulting mitochondria are not equally functional and cannot accomplish respiration and ATP production as in the WT HuR expressing HuH-7 cells. Mitochondria in the HuH-7 cells expressing K120/182R HuR are dispersed rather than forming networks, produce more ROS, and show a lower membrane potential. Furthermore, ER function seems to be compromised in the HuH-7 cells lacking HuR SUMOylation.

### SUMOylation modulates HuR RNA-binding affinity to confer a tumoral phenotype in human hepatoma cells

A final approach to unravel the molecular mechanisms by which HuR SUMOylation controls HCC development and progression was to identify all the differential RNA targets with which HuR interacts in the SUMOylated and non-SUMOylated state. To this end, V5-tagged HuR immunoprecipitation followed by the isolation and sequencing of the bound RNAs (RIP-seq) was performed in the HuH-7 cell line stably expressing WT and the K120/182R mutant HuR. RNA-seq of the input fraction was considered for normalization, as it constitutes a good estimation of total RNA content.

First, a quality control analysis of the samples was performed. A Pearson correlation on raw counts as well as a principal component analysis (PCA) on transformed counts indicated that replicate samples within groups were homogeneous and clearly differentiated from each set of conditions. Next, a comparison of comparisons analysis was performed on RNA-seq data, which consists of the difference between the RNAs bound to K120/182R and WT HuR relative to the difference between the input RNA in K120/182R and WT HuR expressing HuH-7 cell lines.

**Figure 6.**
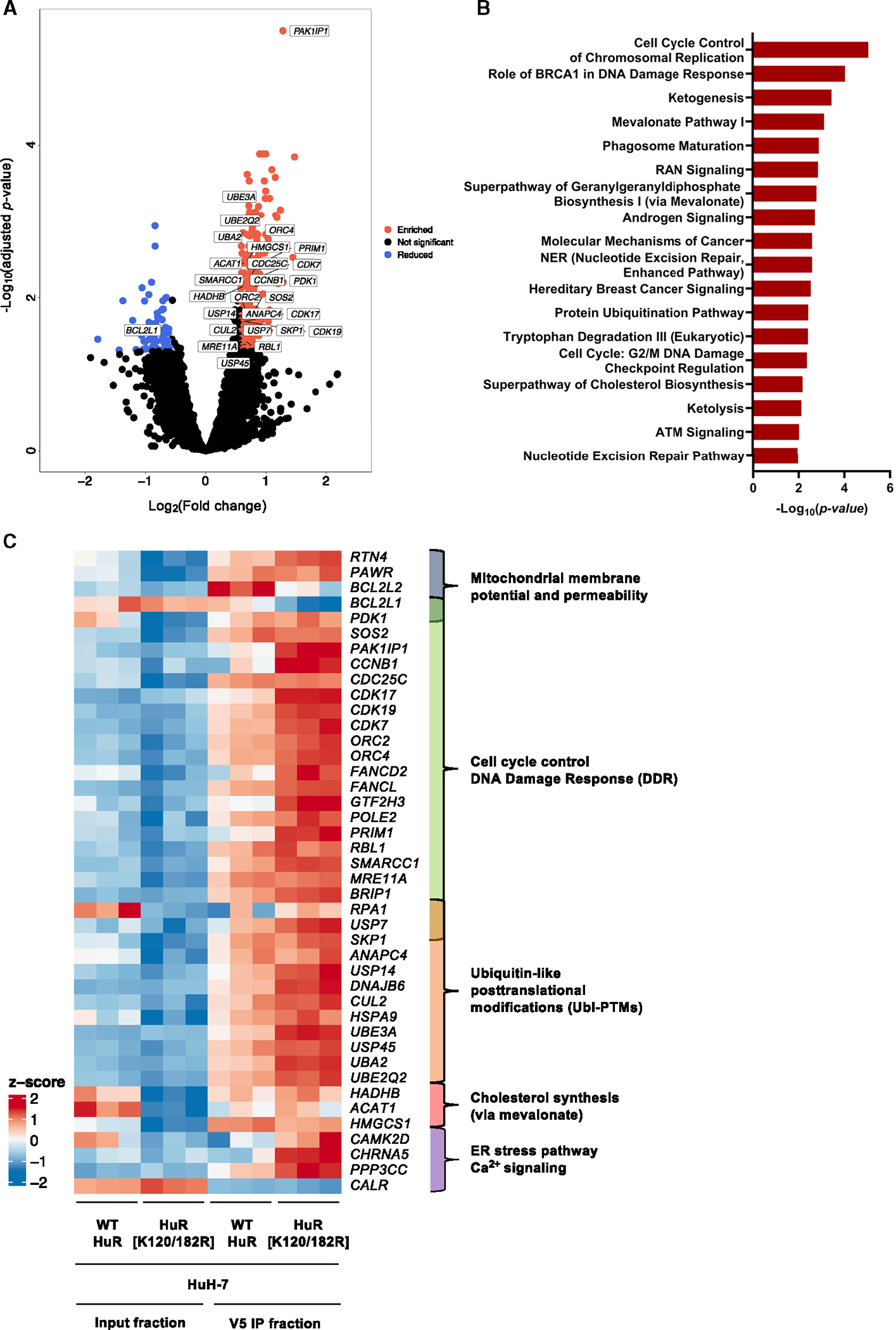
SUMOylation modulates HuR RNA-binding affinity to confer a tumoral phenotype in human hepatoma cells (A) Volcano plot representing all of the identified RNAs bound to V5-tagged HuR after RIP-seq in the HuH-7^WT HuR^ and HuH-7^HuR [K120/182R]^ cells. Blue and red indicate the transcripts showing more than a 1.5-fold change enrichment and p_adj_ < 0.05 in the WT and K120/182R HuR expressing HuH-7 cells, respectively, after performing a background correction with the corresponding input fraction. Black represents RNAs showing no statistically significant enrichment. (B) List of the top most statistically significant canonical pathways identified by IPA software and associated with the enrichment of transcripts most significantly bound to V5-tagged WT and K120/182R HuR in the HuH-7 cell line. (C) Heatmap representing the significantly enriched RNAs related with mitochondrial membrane potential and permeability, cell-cycle control DDR, Ubl-PTMs, cholesterol synthesis via mevalonate, ER stress pathway, and Ca^2+^ signaling in the V5-bound and input fractions of HuH-7^WT HuR^ and HuH-7^HuR [K120/182R]^ cells after performing RIP-seq.

A volcano plot shows the changes in RNAs binding to the K120/182R HuR SUMOylation mutant when compared to WT HuR in the HuH-7 cell line (Figure 6A). The transcripts showing more than a 1.5-fold change enrichment and adjusted p value (p_adj_) < 0.05 were considered to have a significantly changed interaction with the SUMOylation mutant in contrast to WT HuR. Thus, the use of this criterion rendered a list of 346 RNAs showing a reduced or enhanced binding to non-SUMOylated HuR in comparison to the WT variant, which are represented as blue and red dots, respectively in Figure 6A. A first observation was that the K120/182R HuR SUMOylation mutant is capable of strongly interacting with a greater number of RNAs than the WT version (287 and 59 molecules, respectively).

The RNAs showing a significantly differential enrichment after the comparison of comparisons analysis were plotted in a heatmap, which contains the abundance of each transcript expressed as normalized counts both in the V5-bound and input fractions of the HuH-7 cells stably expressing WT and K120/182R mutant HuR. This set of RNAs was further subjected to pathway analysis by using Ingenuity Pathway Analysis (IPA) software, and the top most significantly represented pathways were plotted (Figure 6B). Mostly, the retrieved pathways can be classified into three main groups, i.e., cell-cycle control and DDR, cholesterol biosynthesis via mevalonate, and Ubl-PTMs (Figure 6C). In addition, groups of transcripts related to mitochondrial membrane potential and permeability, ER stress response, and Ca^2+^ signaling were identified.

In essence, SUMOylation compromises HuR intrinsic RNA-binding ability resulting in changes in the transcriptomic profile. Non-SUMOylated HuR shows an enhanced interaction with RNAs related to cell-cycle control and DDR, mitochondrial and ER functionality, and Ubl-PTMs, which would explain the observed senescent phenotype and regulation of the SUMOylation process in the HuH-7 cell lines, as SUMOylated HuR would continue driving HCC progression.

### Xenograft tumors from human hepatoma cells lacking HuR SUMOylation sites show delayed growth and expression of senescence protein markers in mice

**Figure 7.**
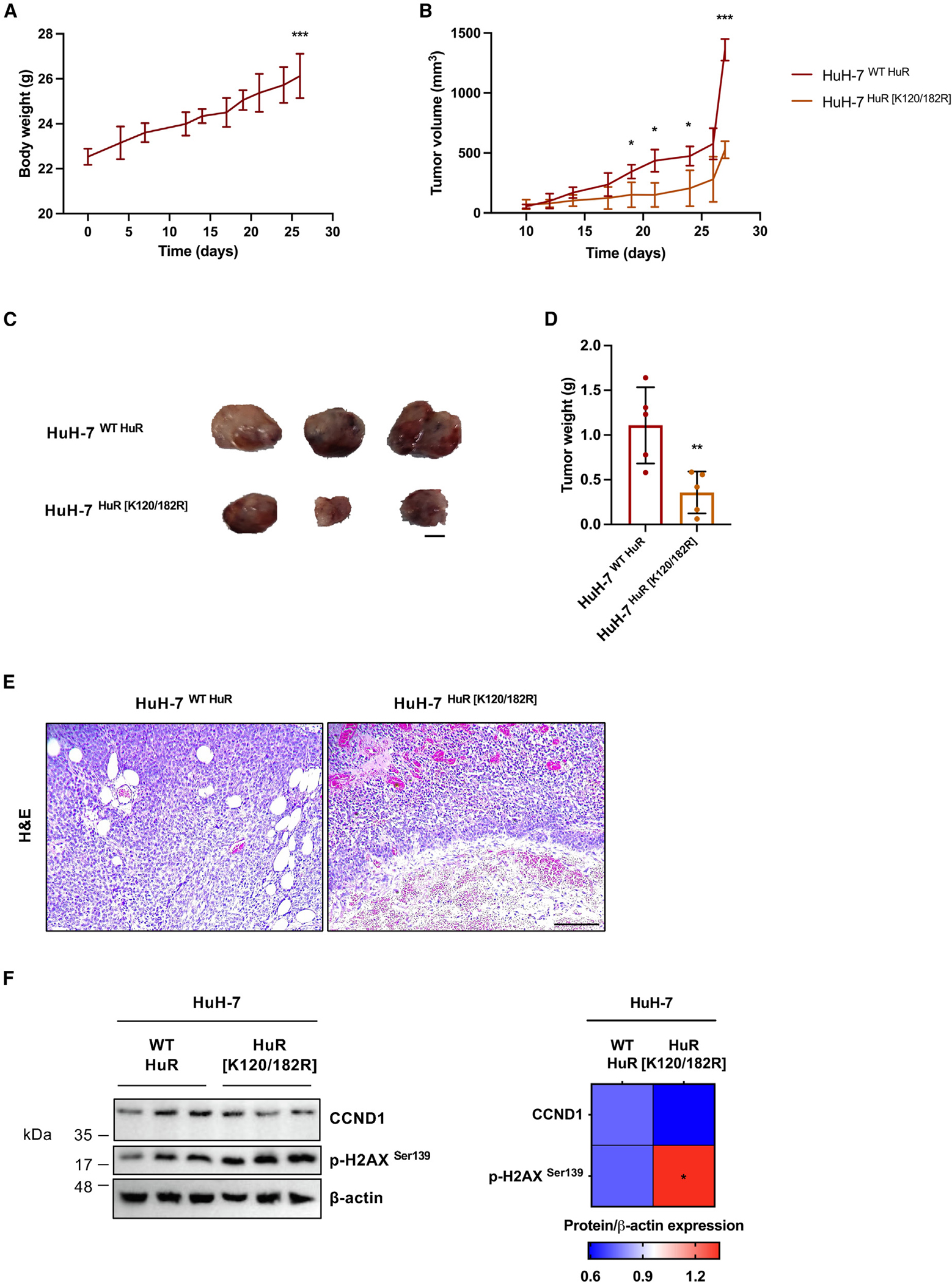
Xenograft tumors from human hepatoma cells lacking HuR SUMOylation sites show delayed growth and expression of senescence protein markers in mice (A and B) (A) Body weight and (B) tumor volume evolution over time after the subcutaneous injection of HuH-7^WT HuR^ and HuH-7^HuR [K120/182R]^ cells in each flank of NSG mice (n = 6). (C–F) (C) Macroscopic appearance, (D) weight, (E) H&E staining, and (F) CCND1 and p-H2AX^Ser139^ protein expression levels and quantification relative to β-actin in the human xenograft tumors derived from HuH-7^WT HuR^ and HuH-7^HuR [K120/182R]^ cells 4 weeks after implantation in NSG mice (n = 6). In (C), (E), and (F), images are representative of at least three biological replicates within one representative experiment. Scale bars represent 1 cm in (C) and 200 μm in (E). In (A), (B), (D), and (F), data are presented as the mean ± SD of at least three biological replicates within one representative experiment. *p < 0.05, **p < 0.01, and ***p < 0.001, two-tailed t test. If not indicated otherwise, the differences were not significant.

The significance of blocking HuR SUMOylation was eventually verified *in vivo* by generating xenograft tumors via the subcutaneous injection of the HuH-7 human hepatoma cell line stably expressing the WT and K120/182R HuR variants in each flank of NOD scid gamma (NSG) mice (n = 6). Tumors appeared 1 week following implantation and were allowed to grow for 4 weeks, significantly increasing mouse body weight (Figure 7A). More importantly, however, monitoring tumor volume over time confirmed that tumors derived from HuH-7 cells expressing the K120/182R HuR SUMOylation mutant showed a slower growth rate compared to those derived from cells harboring WT HuR (Figure 7B). Moreover, xenograft tumor size and weight were significantly decreased in the absence of HuR SUMOylation 4 weeks after HuH-7 cell implantation (Figures 7C and 7D). Regarding the microscopic characterization, hematoxylin and eosin (H&E) staining revealed a reduced vascularization in the HuH-7 xenograft tumors expressing K120/182R HuR, possibly leading to the appearance of necrotic regions (Figure 7E). Interestingly, inhibition of HuR SUMOylation in the HuH-7 cell line resulted in tumors preserving the senescent phenotype, as per decreased CCND1 and increased p-H2AX^Ser139^ protein expression levels (Figure 7F).

In short, the absence of HuR SUMOylation sites has a proven significant inhibitory effect on HuH-7 xenograft tumor growth in mice in addition to the expression of senescence protein indicators.

## DISCUSSION

It is well established that the expression of the RBP HuR is upregulated in many tumor types and is considered a hub in cancer because of the function that it exerts on its target RNAs, which contribute to the main hallmarks of cancer.^50^ Several works had already reported increased HuR protein expression in the context of liver cancer.^57,58^ Accordingly, in this study we detected high *ELAVL1* mRNA expression levels in a cohort of patients with HCC, independently of the tumor stage, which were associated with a lower individual survival.

Interestingly, along with these data, we observed that *ELAVL1* mRNA expression was positively related to the Ubl-PTM SUMOylation in HCC tissue. As introduced, PTMs critically influence HuR function,^60,62–69^ in addition to being currently considered attractive therapeutic targets in cancer.^3^ Together with our earlier discovery that neddylation stabilized HuR leading to its increased abundance in HCC,^66^ here we demonstrate that HuR SUMOylation may also contribute to liver tumor progression by affecting its intrinsic RNA-binding affinity, further modifying the transcriptomic profile.

HuR had been formerly identified as a SUMOylation target through large-scale quantitative proteomics performed in labeled HeLa cells stably expressing His_6_-SUMO2 in an attempt to explain the crosstalk between the SUMO cycle and the ubiquitin-proteasome system (UPS).^92^ As protein-level SUMO proteomics evolved into site-specific approaches, a handful of SUMO-modified residues were disclosed for HuR in the HEK293, HeLa, and U2OS cell lines, especially under stressful conditions (e.g., heat shock and proteasome inhibition), by means of procedures based on exogenously expressed, epitope-tagged mutated SUMO variants, such as His_6_-SUMO2^T90K93,94^ or lysine-deficient His_10_-SUMO2^Q87R^,^11^ as well as an endogenous and native method relying on the commercially available SUMO2/3 8A2 antibody.^95^ However, none of these works managed to identify endogenous HuR SUMOylation in the tissue of different species or studied HuR SUMOylation individually. Thus, here not only have we described that HuR is SUMOylated in human hepatoma cell lines, the *MYC*;*Trp53*^−*/*−^ genetically engineered mosaic mouse model of liver cancer and clinical HCC samples, but we have also established its pathophysiological relevance by elucidating the underlying mechanism relating HuR SUMOylation to tumor progression.

We have comprehensively characterized HuR SUMOylation in the MLP-29 and HuH-7 cell lines as a process consisting in the covalent addition of one or multiple SUMO2/3 subunits into Lys120 and 182, both located in the RRM2, which forms a cleft with the RRM1 for RNA binding. We have additionally identified PIAS 2β and SENP1, 2, and 3 as the principal SUMO E3 ligase and deSUMOylating enzymes for HuR, respectively. Our data regarding HuR SUMOylation sites do not entirely match those experimentally proposed.^11,93–95^ Most HuR SUMO acceptor lysines revealed by site-specific MS-based proteomics were identified in response to stress, and different sites were preferably modified depending on the cell line and insult. This variability could also be attributed to technical differences between studies. Moreover, it is undeniable that SUMOylation co-exists with the rest of the PTMs that regulate HuR (i.e., methylation, phosphorylation, proteolytic cleavage, ubiquitination, neddylation, PARylation, sulfhydration, and arginylation). A study revealed that nearly one-quarter of the SUMOylation sites identified by MS/MS overlap with ubiquitination in the human proteome.^79^ Ubiquitination at Lys182 was previously reported to facilitate HuR degradation in response to heat shock in the HeLa cell line.^65^ Conversely, even though HuR exhibited ubiquitination in the HuH-7 human hepatoma cell line, neither Lys182 nor Lys120 was identified as a major ubiquitination site. Nevertheless, the fact that SUMO and ubiquitin can modify the same acceptor lysine does not necessarily entail competition^96^ or successive modifications.^97^ Ubiquitin, and especially SUMO, are only conjugated to a small subset of a given protein, making it possible for both modifiers to be present on the same lysine at the same time but in different subpopulations of the target proteins.^98^ Also, collaborative crosstalk between SUMO and ubiquitin has been described in the context of proteasomal degradation^99,100^ and DDR.^101^ The enzymes involved in this type of communication are the STUbLs and the counteracting proteases. Accordingly, we anticipated that HuR ubiquitination might depend on SUMOylation in the HuH-7 human hepatoma cell line under certain circumstances, even though a mechanistic explanation on this interplay is not provided. Apart from mixed SUMO-ubiquitin chain formation, which may additionally harbor phosphorylation and acetylation,^94^ SUMO can be conjugated to NEDD8, further increasing signaling complexity, but this is considered a rare event.^11^ In liver cancer, E3 ligase murine double minute 2 (Mdm2) mediates neddylation at Lys283, 313, and 326 of RRM3, leading to increased HuR nuclear localization and reduced proteasomal degradation.^66^ Another site-specific mapping of the human SUMOylome revealed that crosstalk between SUMO and other PTMs (i.e., ubiquitination, methylation, and acetylation) may also occur by proximal modification of the same protein.^11^ Notably, the identified SUMO-methyl-co-modified proteins were enriched for RNA-binding properties. HuR methylation at Arg217 by co-activator-associated arginine methyltransferase 1 (CARM1) has been extensively reported to positively regulate the transcription of its target RNAs,^62,102,103^ and loss of HuR methylation has been observed in HCC causing increased *MAT2A* mRNA and protein expression and subsequent lower *S*-adenosylmethionine (SAMe) levels.^57^ Besides this, 9% of the identified human SUMOylome was reported to occur proximal to phosphorylation, and numerous SUMOylation sites were found to be fully dependent on prior phosphorylation events,^11^ a result in agreement with the earlier described phosphorylation-dependent SUMOylation motif (PDSM).^104^ Interestingly, it is well established that the checkpoint kinase 2 (Chk2)^63,105^ and p38 mitogen-activated protein kinase (MAPK)^106,107^ can modulate HuR RNA binding through the phosphorylation of Thr118, which is located close to SUMO acceptor Lys120. Overall, we highlight the potential for exploring the endogenous interplay between SUMOylation and the different PTMs that control HuR in a unified context.

Apart from describing HuR SUMOylation at the molecular level, we were interested in further studying its effect on liver cancer progression. For this purpose, the HuH-7 HCC cell-line model stably expressing the WT and SUMOylation mutant HuR variants were subjected to a phenotypic characterization. On the one hand, HuH-7 cells bearing WT HuR showed higher proliferative and invasive potential than those expressing the SUMOylation mutant. Thus, the absence of HuR SUMOylation resulted in a senescent phenotype consisting of lower proliferation and invasion ratios as well as elevated β-galactosidase activity and expression of senescence protein markers, which were exacerbated after palbociclib-mediated CDK4/6 inhibition. Importantly, HuH-7 xenograft tumors expressing the K120/182R HuR SUMOylation mutant preserved the attenuated growth rate and senescent phenotype. On the other hand, mitochondrial structure and function were further examined, as they are known to be affected during cellular senescence.^90^ The interception of HuR SUMOylation in the HuH-7 cell line firstly revealed a rounder and more dispersed mitochondrial network. The resulting mitochondria lacked functionality and could not attain respiration as efficiently as cells expressing WT HuR. Also, ATP and NAD^+^/NADH levels were reduced, being consistent with a suppressed energy production in the absence of HuR SUMOylation. Furthermore, these defective mitochondria showed decreased membrane potential with high ROS generation, possibly derived from a perturbed electron transport chain (ETC). In addition to mitochondria, there was an increase in the ER mass, and its function was badly affected by the inhibition of HuR SUMOylation in human HCC cells, all conforming with the senescent phenotype.^89,90^

HuR being an RBP and considering that SUMOylation sites are located near or in the basic cleft involved in RNA recognition and binding, we investigated whether SUMOylation could modify HuR intrinsic RNA-binding affinity. Not surprisingly, MD simulations predicted a considerably less compact structural arrangement of the RRM1 and 2 and the concealment of the residues involved in RNA recognition upon SUMOylation. These conformational changes could modulate the binding affinity of HuR for its target mRNAs and eventually regulate the transcriptomic profile, as was later corroborated by RIP-seq studies. Accordingly, the K120/182R HuR SUMOylation mutant showed an enhanced interaction with a larger number of transcripts than WT HuR. Importantly, the displayed *in silico* and *in vitro* data also confirmed that the observed differential RNA binding for each of the HuR SUMOylated species is not caused by the lysine-to-arginine mutations but is entirely driven by SUMOylation. The mRNAs displaying a differentially enriched interaction with the K120/182R HuR SUMOylation mutant were mainly involved in cell-cycle control and DDR, cholesterol biosynthesis via mevalonate, and Ubl-PTMs, which matched with the phenotype observed along this study, as discussed later.

Under physiological conditions HuR is located in the nucleus, where it participates in mRNA splicing and nuclear export. However, upon specific stimuli, HuR translocates to the cytoplasm and elicits its best-understood effects on mRNA stabilization and modulation of translation. Importantly, although cytoplasmic HuR normally promotes translation, it can also repress it.^47,108^ Therefore, HuR can affect the translation of the enriched mRNAs identified after the RIP-seq analysis in both directions.

The loss of cell-cycle control and DNA damage are renowned hallmarks of senescence and have also been detected in the absence of HuR SUMOylation through the enrichment of a considerable subset of transcripts involved in cell-cycle regulation and DDR that significantly interact with non-SUMOylated HuR in the HuH-7 cell line, being consistent with the senescent phenotype manifested throughout this study. Moreover, the observed senescence-associated organelle damage was evidenced in the RIP-seq analysis by the enrichment of mRNAs involved in mitochondrial membrane potential and permeability, ER stress response, and Ca^2+^ signaling, which showed an increased interaction with the K120/182R HuR SUMOylation mutant. Interestingly, the mevalonate pathway for cholesterol biosynthesis was another significantly represented route in the RIP-seq analysis, which is known to play an important role in the progression of many types of cancer, including HCC, and may now be connected with HuR SUMOylation.^109–112^

Finally, changes in the binding of HuR upon SUMOylation to mRNAs related with Ubl-PTMs are of particular relevance to this project, as they help reinforce the unequivocal relationship between HuR and SUMOylation. In this study, not only have we demonstrated that HuR is SUMOylated but we also leave open the possibility that HuR SUMOylation could be governing global SUMOylation processes through changes in RNA binding, eventually contributing to control the fate of liver tumors. On the one hand, we observed that HuR SUMOylation increased the levels of SUMO-conjugated proteins, possibly promoting protein SUMOylation and senescence escape. Complementarily, the oxidizing cellular environment observed in the absence of HuR SUMOylation could explain the reduced SUMO conjugation due to disulfide bridge formation between UBA2 and UBC9 catalytic cysteines.^113^ On the other hand, the differential enrichment analysis of the mRNAs bound to non-SUMOylated HuR revealed a significant number of transcripts involved in the SUMOylation pathway whose translation could be affected. Notably, the *UBA2* transcript encoding one subunit of the SUMO-activating enzyme heterodimer was detected. Interestingly, *UBA2* mRNA expression was significantly induced and strongly correlated with *ELAVL1* in the tumor, in contrast to the paired ST of a cohort of patients with HCC. Overall, a model emerges from our results whereby HuR SUMOylation may be controlling the translation of mRNAs involved in the SUMO pathway eventually modulating the number of SUMO-conjugated proteins, including HuR itself, which could be playing a role in HCC progression.

Taking our evidence together, our results unveil conceptual and functional avenues in HCC, with potential clinical implications, by demonstrating that HuR is a SUMOylation substrate in clinical HCC as well as in *in vivo* and cellular models of the disease. Specifically, HuR SUMOylation is likely to occur as the covalent addition of SUMO2/3 subunits into Lys120 and 182 catalyzed by PIAS 2β and reversed by the action of SENP1, 2, and 3 in the MLP-29 and HuH-7 cell lines. Importantly, we have established the pathological implication of HuR SUMOylation in liver cancer. Thus, SUMOylated HuR contributes to tumor cell proliferation and invasion, while its absence results in a senescent phenotype with damaged mitochondrial and ER structure and function. Regarding the mechanism of action, SUMOylation alters HuR intrinsic RNA-binding affinity, resulting in the modulation of the transcriptomic profile driving HCC progression. In conclusion, SUMOylation constitutes a mechanism of HuR regulation that could be potentially exploited as a therapeutic strategy for the clinical management of liver cancer, thus highlighting the value of PTMs as disease targets. Even though potent promising SAE inhibitors have been developed in the last decade,^114–118^ the abolition of global SUMOylation may not be entirely encouraged because of the cellular processes that this Ubl-PTM regulates independent of cancer development. Also, given the key function of SUMOylation in cell-cycle progression, exploring combination strategies with cell-cycle inhibitors has been recommended.^20^ Hence, a combination therapy for HCC based on HuR SUMOylation inhibition and palbociclib administration may emerge as a result of this study and could be particularly beneficial to patients, since single administration of CDK4/6 inhibitors is often suboptimal for the treatment of these malignancies.^87^ Furthermore, understanding the effects of HuR SUMOylation in hepatocarcinogenesis will provide insights into the relatively unknown role of SUMOylation in cancer.

### Limitations of the study

We acknowledge that the tools to study PTMs are not yet fully developed, thereby limiting the interpretation of experimental data. For example, the use of nickel-His_6_-SUMO2/3 affinity purification to elucidate the canonical deSUMOylating enzyme for HuR rendered slightly vague results depending on whether this technique was preceded by up- or downregulation of protein expression. The lack of HuR protein enrichment was more evident when overexpressing SENP1, 2 and 3, while the presence of SUMOylated HuR was less convincing when knocking down *Senp1* and *2* expression in the MLP-29 cell line. Along this line, the analysis of the interplay between the different PTMs is also restricted. Here, we report that HuR is modified both by SUMOylation and ubiquitination in liver cancer cells even though they involve different sites. Moreover, we propose that HuR ubiquitination might occur as a SUMOylation-dependent process, but we do not further provide a mechanistic explanation for this crosstalk, encouraging others to do so. Nevertheless, we are hopeful that ongoing research will help to propel the field of PTMs.

## STAR★METHODS

Detailed methods are provided in the online version of this paper and include the following:

### RESOURCE AVAILABILITY

#### Lead contact

Further information and requests for resources and reagents should be directed to and will be fulfilled by the lead contact, María Luz Martínez-Chantar (mlmartinez@cicbiogune.es).

#### Materials availability

Plasmids and stably transfected cell lines generated in this study are available upon request to María Luz Martínez-Chantar (mlmartinez@cicbiogune.es).

#### Data and code availability

- RIP-Seq data have been deposited at Gene Expression Omnibus (GEO) and are publicly available as of the date of publication. The accession number is GSE197798 and is also listed in the key resources table.
- This paper does not report original code.
- Any additional information required to reanalyze the data reported in this paper is available from the lead contact upon request.

### EXPERIMENTAL MODEL AND STUDY PARTICIPANT DETAILS

#### Animals

Liver tissue samples of the *MYC*;*Trp53*^−*/*−^ genetically engineered mosaic mouse model of HCC were kindly provided by Dr. Amaia Lujambio.^74^ The oncogenic xenograft murine procedure included in project P-CBG-CBBA-0722 was approved by the CIC bioGUNE Institutional Animal Care and Use Committee and the competent authority from Diputación de Bizkaia. Animal experimentation was conducted in accordance with the National Institutes of Health (NIH) guide for care and use of Laboratory animals and the guidelines of the European Research Council for animal care and use. Young 7-weeks old female NOD scid gamma (NSG) mice (614, Charles River Laboratories) were housed in an Association for Assessment and Accreditation of Laboratory Animal Care (AAALAC)-ac-credited animal facility at CIC bioGUNE under controlled temperature (21 ± 1°C) and humidity (45 ± 10%) conditions, with 12-h light/dark cycles and *ad libitum* access to Teklad global 14% protein rodent maintenance diet (2014C, Envigo) and water. For the procedure, a total of 5 million HuH-7 cells stably expressing WT HuR and the K120/182R SUMOylation mutant were subcutaneously injected in each flank of mice (n = 6). Tumor size was monitored with a digital caliper every 3 days from day 10 after implantation and mice were euthanized before tumors exceeded 1,500 mm^3^. Tumor volume (V) was calculated by using the modified version of the elipsoidal formula: *V = ½* (*Length* 3 *Width*^*2*^). Tumor masses were collected for western blotting and histological analyses.

#### Human participants

A cohort of 172 formalin-fixed paraffin-embedded (FFPE) samples consisting of paired HCC tumor and surrounding non-tumor tissue were obtained from the Córdoba Node of the Andalusian Public Health System Biobank, evaluated by liver histology and the diagnosis was confirmed by two independent, experienced pathologists. Clinical data from patients was collected from electronic medical reports (Table S1). The study protocol was approved by the Reina Sofia University Hospital Ethics Committee, according to institutional and Good Clinical Practice guidelines (Protocol number PI17/02287) and in compliment with the declaration of Helsinki. Informed consent was obtained from all patients or their relatives. Also, a cohort of 5 patients including paired HCC tumor and surrounding tissue liver biopsies obtained during tumor resection was provided by the Basque Biobank upon informed consent and with evaluation and approval from the corresponding ethics committee.

#### Cell lines

The THLE-2 (CRL-2706, ATCC), PLC/PRF/5 (CRL-8024, ATCC), HuH-7 (JCRB0403, JCRB Cell Bank) and MLP-29 (provided by Dr. Enzo Medico)^119^ cell lines were grown in the medium stated in the manufacturer’s instructions. All cell lines were maintained in culture for a maximum of 20 passages in a humidified incubator at 37°C and 5% CO_2_, unless otherwise stated.

### METHOD DETAILS

#### Hematoxylin and eosin (H&E) staining

5 μm-thick paraffin-embedded sections of the formalin-fixed xenograft tumor samples were deparaffinized with Histo-Clear (HS-200, National Diagnostics) during 20 min and rehydrated through graded ethanol solutions (100–70%) to distilled water. Sections were incubated with Harris hematoxylin (05–06004, Bio-Optica) for 15 min, rinsed in running tap water, and differentiated with 0.5% HCl. Specimens were rinsed with distilled water and subsequently stained with aqueous Eosin Y solution (HT110232, Sigma-Aldrich) for 15 min. Finally, samples were dehydrated through graded ethanol solutions (70–100%), cleared with Histo-Clear and mounted with DPX mounting medium (06522, Sigma-Aldrich). Images were acquired with an ×10 magnification objective in a DM750 upright microscope (Leica) equipped with a ICC50W camera (Leica).

#### Plasmid generation

The full length cDNA of WT mouse HuR was purchased from the German Resource Center for Genome Research (RZPD). The V5-HuR WT plasmid was constructed by PCR using a 5′ oligonucleotide containing the V5 tag sequence and being subcloned into a pcDNA3.3-TOPO vector (K830001, Invitrogen).^66^ SUMOylation sites on HuR were predicted experimentally by mutation of lysine residues into arginine. The SUMOylation mutant HuR plasmid constructs were created using the QuickChange site-directed mutagenesis kit (200518, Stratagene), according to the manufacturer’s instructions, with two complementary oligonucleotides and with pcDNA3.3-TOPO-V5-HuR WT plasmid as template. Products were sequenced by STAB vida. Plasmid DNA was purified after bacterial transformation and amplification, using NucleoBond Xtra Midi Plus kit (740412, Macherey-Nagel), by following the manufacturer’s instructions.

#### Plasmid DNA transfection

Cells were transfected with the different plasmids summarized in KRT,^120–130^ using Lipofectamine 2000 transfection reagent (11668019, Invitrogen) and Opti-MEM I reduced serum medium (31985070, Gibco), as stated in the manufacturer’s instructions. Cells were allowed to grow for additional 48 h until optimal protein expression. In order to create stable cell lines, pcDNA3.3-TOPO-V5-mHuR plasmid constructs were digested with *PvuI* restriction enzyme (ER0621, Thermo Scientific) prior to transfection, by following the manufacturer’s indications. Linearization avoids unspecific cleavage and increases the chances that the vector integrates into the host cell genome without disrupting the gene of interest or other elements required for expression in mammalian cells. The transfected cells were selected and maintained in culture medium containing 1.5 mg/mL Geneticin (G418 sulfate) selective antibiotic (11811031, Gibco). Cell clones were obtained by a serial dilution process in 96-well plates and colonies were then subcultured into larger dishes. Transfection efficiency of each clone was confirmed by western blotting.

#### siRNA transfection

Cells were transfected with Silencer Select negative control no.1 siRNA (4390843, Invitrogen) or the different Silencer Select siRNAs (4390771, Thermo Fisher Scientific) summarized in the **KRT**, using DharmaFECT transfection reagent (T-2001–03, Horizon Discovery) and Opti-MEM I reduced serum medium (31985070, Gibco), by following the manufacturer’s instructions. Cells were allowed to grow for additional 48 h until optimal mRNA expression knockdown, which was validated by qPCR.

#### Hypoxia

Cells were incubated in a Bugbox M anaerobic workstation (Baker) at 1% O_2_ during 24 h.

#### ML-792 treatment

ML-792 (HY-108702, MedChemExpress) was administered *in vitro* at 100 nM in cell culture medium during 4 h. Under no circumstances was DMSO final volume greater than 0.1%.

#### Palbociclib treatment

Palbociclib isethionate salt (P-7766, LC Laboratories) was dissolved in DMSO and stored at −80°C. The drug was administered *in vitro* both as an acute and a chronic treatment. On the one hand, the acute treatment involved the administration of higher concentrations ranging from 0 to 1 μM palbociclib for 3 days. On the other hand, the chronic treatment involved the administration of lower concentrations ranging from 0 to 100 nM palbociclib for 2 weeks. During this period of time, medium and treatment were renewed every 3 days, as cells were subcultured. Under no circumstances was DMSO final volume greater than 0.1%.

#### Bioinformatic analysis

The results published are based upon data generated by the The Cancer Genome Atlas (TCGA) Research Network (https://www.cancer.gov/tcga). Expression levels of the indicated genes in non-tumor and tumor tissue of HCC patients, as well as in the different tumor stages were expressed as RNA-Seq by Expectation Maximization (RSEM). Survival information of patients with HCC was obtained from the clinical information dataset. Patients were divided into two groups based on low and high expression levels of *ELAVL1* when mRNA levels were below or above the median, respectively. Data was plotted as Kaplan-Meier curves and the Mantel-Cox test was performed for statistical comparison between the two groups. The Gene Set Enrichment Analysis (GSEA) software (https://www.gsea-msigdb.org/gsea/) was used to perform an enrichment pathway analysis based on *ELAVL1* expression levels in HCC patients. By establishing the median as a cut-off value, the enriched pathways when *ELAVL1* mRNA levels were low and high were designated with a normalized enrichment score (NES). In this study, only the pathways showing a NES greater than a 2-fold change, p value <0.0001 and FDR <0.01 were considered.

#### Total RNA isolation

Total RNA from cell lines and FFPE tissues was extracted with TRIzol reagent (15596026, Invitrogen) and the Maxwell 16 LEV RNA FFPE Purification Kit (AS1260, Promega), respectively, as per manufacturer’s instructions. RNA concentration was determined in the NanoDrop 1000 spectrophotometer (Thermo Fisher Scientific).

#### Reverse transcription (RT)

1–2 μg of RNA were treated with Amplification Grade DNase I (18068015, Invitrogen) by following the manufacturer’s guidelines. cDNA was synthesized with M-MLV reverse transcriptase (28025013, Invitrogen) in the presence of Random Primers (48190011, Invitrogen), dNTPs (10297018, Invitrogen), and RNaseOUT recombinant ribonuclease inhibitor (10777019, Invitrogen), by using a Veriti Dx thermal cycler (Applied Biosystems). RT conditions involved 10 min at 25°C, 3h at 37°C and 15 min at 70°C. The resulting cDNA was diluted 10-fold in nuclease-free water (W4502, Sigma-Aldrich).

#### Quantitative PCR (qPCR)

Gene primer sequences were designed with Primer-BLAST tool (https://www.ncbi.nlm.nih.gov/tools/primer-blast/) and synthesized by Sigma-Aldrich (Table S2). For conventional qPCR, 1.5 mL of cDNA were mixed with specific primers and SYBR Select master mix (4472908, Invitrogen) constituting a final volume of 6.5 mL, in MicroAmp Optical 384-Well Reaction Plates (4309849, Applied Biosystems). Each reaction was performed in triplicate using the ViiA 7 Real-Time PCR system (Applied Biosystems). qPCR conditions involved an initial denaturation step (90 s at 95°C), followed by 40 cycles of annealing (15 s at 95°C and 1 min at 59°C), and a final extension phase (15 s at 95°C, 1 min at 60°C and 15 s at 95°C). Ct values were extrapolated from the melt curve and gene expression levels were normalized with *RPLP0* or *Gapdh* housekeeping expression by implementing the 2^ΔΔCt^ formula.

#### qPCR dynamic array based on microfluidic technology

A microfluidic-based qPCR dynamic array was used for the RNA expression analysis in samples derived from the Andalusian Public Health System Biobank cohort of patients.^133–135^ Specific primers for human transcripts were designed with Primer-BLAST tool (https://www.ncbi.nlm.nih.gov/tools/primer-blast/) and synthesized by Sigma-Aldrich (Table S2). Preamplification, exonuclease treatment and qPCR dynamic array based on microfluidic technology were implemented using the Biomark System (Fluidigm) by following the manufacturer’s instructions.^136^ mRNA copy number of the transcripts analyzed were adjusted by normalization factor, calculated with the expression levels of *ACTB*, *GAPDH* and *HPRT* using geNorm 3.3 software.^137^ Pearson correlation analyses were computed on mRNA expression levels and reported the values of the correlation coefficient (r) and two-tailed p values. The coefficient of determination (R^2^) was calculated from the Pearson correlation coefficient, and the best-fit line was plotted for each correlation.

#### Ribonucleoprotein immunoprecipitation (RIP)

In order to identify the RNAs interacting with SUMOylated and non-SUMOylated HuR, the WT and K120/182R variants were immunoprecipitated with anti-V5 coated Protein G Sepharose resin and the RNA content bound to HuR was isolated and sequenced.^57,66^ Cells were seeded in 10 cm tissue-culture treated dishes and allowed to grow until they reached 80–100% confluency. For each reaction tube, 50 μL of Protein G Sepharose 4 Fast Flow (GE17–0618-01, Cytiva) beads slurry were washed twice with 1 mL of cold NT2 buffer (50 mM Tris pH 7.4, 150 mM NaCl, 1 mM MgCl_2_, 0.05% NP40) by centrifugation at 5,000 × g, 5 min, 4°C. After discarding the supernatant, beads were incubated with 10 μg of V5 Tag monoclonal antibody (R960–25, Invitrogen) in 150 μL NT2 buffer overnight under rotation at 4°C. For sample precleaning tubes, 25 μL of Protein G Sepharose beads slurry were prepared and incubated with 7.5 μg of Purified Mouse IgG1, κ Isotype Control antibody (557273, BD Pharmingen), as explained. Cells were lysed in polysome lysis buffer (PLB) (100 mM KCl, 5 mM MgCl_2_, 10 mM HEPES pH 7.0, 0.5% NP40) supplemented with 1 mM DTT, 100 U/ml RNaseOUT Recombinant Ribonuclease Inhibitor (10777019, Invitrogen) and cOmplete Mini EDTA-free Protease Inhibitor Cocktail (11836170001, Roche). Lysates were centrifuged twice at 14,000 × *g*, 30 min, 4°C. Approximately 1/10 part of the clarified lysates was reserved for RNA isolation of the input fraction, while the majority of the sample was incubated with the anti-IgG1 precoated beads during 30 min under rotation at 4°C. The supernatant was recovered by centrifugation (10,000 × *g*, 5 min, 4°C) and the presence of protein was confirmed with the Micro BCA Protein Assay Kit. Precleaned samples were next incubated with the anti-V5 precoated beads during 1 h under rotation at 4°C. The beads containing the bound ribonucleoprotein complexes were washed five times with NT2 buffer by centrifugation (5,000 × *g*, 5 min, 4°C). For RNA isolation of the immunoprecipitated material, beads were incubated with Ambion RNase-free DNase I (AM2222, Invitrogen) in 100 μL NT2 buffer for 15 min at 37°C, washed with NT2 buffer (5,000 × *g*, 5 min, 4°C) and further incubated with 0.1% SDS and recombinant PCR Grade Proteinase K (03115828001, Roche) in 100 mL NT2 buffer during 15 min at 55°C while shaking. Protein digestion was stopped with 200 μL NT2 buffer and the supernatant containing the RNA was collected by centrifugation (5,000 × *g*, 5 min, 4°C). For each reaction tube, RNA was extracted with UltraPure Phenol:Chloroform:Isoamyl Alcohol (15593031, Invitrogen) and precipitated overnight in the presence of cold 100% ethanol, 3 M sodium acetate pH 5.5 and 5 μL GlycoBlue Coprecipitant (AM9516, Invitrogen). Samples were centrifuged at 14,000 rpm, 30 min, 4°C. The RNA pellet was washed with cold 70% ethanol and centrifuged at 14,000 rpm, 10 min, 4°C, air dried for 5 min and gently resuspended in 15 μL of H_2_O (W4502–1L, Sigma).

#### Sequencing library preparation

The quantity and quality of the RNAs were evaluated using Agilent RNA 6000 Pico Chips (5067–1513, Agilent Technologies). Sequencing libraries were prepared using the TruSeq Stranded Total RNA Human/Mouse/Rat kit (RS-122–2201, Illumina Inc.), following the manufacturer’s guidelines. Starting from 16 to 77 ng or 250 ng of total RNA in the case of the RIP or input fraction samples, respectively, which had been previously estimated by Bioanalyzer, rRNA was depleted and remaining RNA was purified with 193 μL of RNAClean XP beads, fragmented 6 min and primed for cDNA synthesis. cDNA first strand was synthesized with SuperScript-II Reverse Transcriptase (18064–014, Thermo Fisher Scientific) for 10 min at 25°C, 15 min at 42°C, 15 min at 70°C and pause at 4°C. cDNA second strand was synthesized with Illumina reagents at 16°C for 1 h. Then, A-tailing and adaptor ligation were performed. Finally, enrichment of libraries was achieved by PCR (30 s at 98°C; 15 cycles of 10 s at 98°C, 30 s at 60°C, 30 s at 72°C; 5 min at 72°C and pause at 4°C). Afterward, libraries were visualized on an Agilent 2100 Bioanalyzer using Agilent High Sensitivity DNA kit (5067–4626, Agilent Technologies) and quantified using Qubit dsDNA HS DNA Kit (Q32854, Thermo Fisher Scientific). Sequencing data were acquired in a NovaSeq6000 system (Illumina Inc.).

#### RIP-seq data analysis

BCL files were de-multiplexed and converted to FASTQ files using bcl2fastq program version 2.20.0.422. FASTQ files were trimmed for adapter sequences using Cutadapt version 1.18 and aligned to human genome hg19 Ensembl version 82 using STAR software version 2.4.0j. featureCounts version 1.6.4 software was used to generate gene counts. Differential expression analysis of the gene counts was carried out with the Bioconductor package DESeq2 version 1.30.0^138^ in R version 4.0.3. For identification of differentially enriched mRNAs in K120/182R HuR V5-IP versus WT HuR V5-IP samples, a design with interaction terms was used to take into account background expression of the total RNA from the input fraction for each transcript in groups. Transcripts were accepted to be differentially enriched with statistical significance Benjamini-Hochberg adjusted p value <0.05 and absolute log_2_ fold change >0.58 (equivalent to 1.5-fold change). The Ingenuity Pathway Analysis software (IPA, Qiagen Inc.) was used to define biological functions of differentially bound mRNAs. For heatmap visualization the ComplexHeatmap version 2.8.0 and ggplot2 version 3.3.5 packages were used.

#### Total protein extraction

Total protein from frozen liver tissue or cell lines was extracted in RIPA lysis buffer (1.6 mM Na_2_HPO_4_, 8.4 mM NaH_2_PO_4_, 0.1 M NaCl, 0.1% SDS, 0.1% Triton X-100) supplemented with 10 mM sodium deoxycholate, 1 mM sodium orthovanadate, 50 mM NaF, protease (P8340, Sigma-Aldrich) and phosphatase inhibitor cocktails (P2850, Sigma-Aldrich), in addition to 10 mM N-ethylmaleimide (NEM) and 10 mM iodoacetamide (IAA) cysteine protease inhibitors, which prevent non-specific deSUMOylation. For tissue homogenization, two cycles of 5,000 rpm for 30s in the Precellys 24 homogenizer (Bertin Instruments) were performed. Lysates were clarified by centrifugation (12,500 rpm, 20 min, 4°C) and total protein concentration from the supernatant was estimated by the Micro BCA Protein Assay Kit (23235, Thermo Fisher Scientific) using a BSA standard curve, in a SpectraMax M2/M2e microplate reader (Molecular Devices). For western blotting analysis, 10–25 μg of total protein were combined with 5x Laemmli sample loading buffer (250 mM Tris pH 6.8, 10% SDS, 50% glycerol, 500 mM β-mercaptoethanol, bromophenol blue).

#### Western blotting

Protein samples were boiled at 95°C for 5 min and separated by SDS-PAGE in 10–15% acrylamide gels using a Mini-PROTEAN tetra cell electrophoresis system (Bio-Rad). Proteins were transferred from gels into 0.2 μm-pore size nitrocellulose membranes (10600001, GE Healthcare) using a Trans-Blot Cell electroblotting system (Bio-Rad). The presence of total protein was detected by Ponceau S solution (P7170, Sigma-Aldrich) staining. Non-specific binding was blocked by incubation of the membranes with 0.1% Tween 20-TBS solution containing 5% skimmed milk powder or BSA (A3912, Sigma-Aldrich) for 1 h at room temperature (RT), prior to addition of the primary antibody (KRT). After washing the unbound primary antibody with 0.1% Tween 20 -TBS three times, membranes were incubated with the corresponding HRP-linked secondary antibody (KRT) for 1 h at RT. Membranes were washed three times to remove the excess of secondary antibody and the Clarity Western ECL substrate (170–5061, Bio-Rad) was subsequently added. The chemiluminescent signal from immunoreactive proteins was detected in the ImageQuant LAS 4000 imaging system (GE Healthcare). Protein bands were quantified by densitometric analysis using the open-source image processing program Fiji software (https://imagej.net/software/fiji/) and normalized to β-actin housekeeping protein expression.

#### Protein pull-down with SUMO binding entities (SUBEs)

The SUMO-interacting proteins from frozen liver tissue or cell line extracts were captured under native conditions with GST-tagged SUBEs bound to a glutathione-agarose resin.^71^ For each reaction tube, 100 μL of the glutathione-agarose (G4510, Sigma-Aldrich) slurry was incubated with 100 μg of either the GST-tagged SUBEs or the GST control in SUBEs buffer (50 mM Tris pH 8.5, 150 mM NaCl, 5 mM EDTA, 1% Igepal) supplemented with 1 mM DTT, constituting a final volume of 500 μL. Pre-binding of GST to the glutathione-agarose beads was allowed to occur overnight at 4°C under rotation. Total protein from liver tissue or cell lines was extracted in SUBEs buffer supplemented with cOmplete mini EDTA-free protease inhibitor cocktail tablets (11836170001, Roche) and 50 μM PR-619 ubiquitin and ubiquitin-like proteases inhibitor (662141, Calbiochem). Protein concentration of the input fraction was determined by the Micro BCA Protein Assay Kit (23235, Thermo Fisher Scientific), and 1–2 mg of protein were added to the pre-incubated mixture of GST or GST-SUBEs and GSH-agarose beads. Proteins were allowed to bind to the GST or SUBEs while rotating for 1 h at 4°C. The bound proteins were recovered by centrifugation (1,000 × *g*, 5 min, 4°C). The supernatant corresponding to the unbound protein or flow-through fraction was discarded. The beads and associated proteins were washed with 30 column volumes of SUBEs buffer for 5 min at 4°C and centrifuged (1,000 × *g*, 5 min, 4°C). The supernatant was carefully discarded and 1 column volume of 5x Laemmli buffer was added in order to elute the GST or SUBEs-bound proteins from the glutathione-agarose beads. The mixture was incubated for 10 min under rotation, boiled at 95°C for 2 min and centrifuged (1,000 × *g*, 5 min). The supernatant corresponding to the GST or SUBEs-bound proteins or elution fraction was subjected to western blotting and mass spectrometry analysis.

#### Mass spectrometry (MS)-based proteomics

Samples were processed using the filter-aided sample preparation (FASP) method.^139^ Peptides were further desalted using ZipTip stage-tip C_18_ microcolumns (Millipore) and resuspended in 0.1% formic acid prior to MS analysis. Samples were loaded onto a timsTOF Pro with parallel accumulation-serial fragmentation (PASEF) mass spectrometer (Bruker Daltonics)^140^ coupled online to a nanoElute liquid chromatograph (Bruker Daltonics) and analyzed in triplicate. Protein identification and abundance calculation were carried out using PEAKS software (Bioinformatics Solutions), and data were further loaded onto Perseus software platform (https://www.maxquant.org/perseus/)^141^ for statistical analysis. Proteins identified with at least two different peptides were considered in the final analysis. A permutation-based FDR-corrected t test was applied for the comparison of the abundances, and proteins with a *q value* < 0.05 and a SUBE/GST ratio greater than 2 were considered as enriched.

#### Nickel-histidine affinity purification

H_6_-tagged SUMO or ubiquitin protein conjugates were purified in a nickel-agarose resin under denaturing conditions.^142^ Low density nickel-agarose beads (6BCL-QLNi-25, ABT) were prepared by washing with 5–10 column volumes of PBS supplemented with 0.1% BSA under rotation for 10 min to remove the storage solution. Beads were centrifuged (1,500 rpm, 5 min) and resuspended in PBS so that a 50% slurry was obtained. Cells were collected in PBS and total protein extraction was performed. Protein concentration of the input fraction was determined by the Bradford assay (5000006, Bio-Rad), and 300–400 μg of protein were added to 3 mL of buffer I (6 M guanidinium-HCl, 0.1 M Na_2_HPO_4_/NaH_2_PO_4_, 0.01 M Tris-HCl pH 8.0, supplemented with 10 mM β-mercaptoethanol, 10 mM imidazole and 0.1% Triton X-100) and 80 μL of the nickel-agarose bead slurry. Binding of the histidine-tagged proteins to the resin was allowed to occur under rotation for 3 h at RT. The beads were collected by centrifugation (1,000 rpm, 5 min), and the supernatant corresponding to the unbound protein fraction was discarded. The proteins bound to the resin were transferred to a new set of tubes and washed with 750 μL of buffer I once, buffer II (8 M urea, 0.1 M Na_2_HPO_4_/NaH_2_PO_4_, 0.01 M Tris-HCl pH 8.0, supplemented with 10 mM β-mercaptoethanol and 0.1% Triton X-100) twice and buffer III (8 M urea, 0.1 M Na_2_HPO_4_/NaH_2_PO_4_, 0.01 M Tris-HCl pH 6.3, supplemented with 10 mM β-mercaptoethanol and 0.1% Triton X-100) three times. After a centrifugation step (2,500 rpm, 5 min), the supernatant was carefully discarded and 50 μL of 3x Laemmli sample buffer containing 200 mM imidazole were added to elute the histidine-tagged proteins from the resin by rotation for 30 min. Samples were finally centrifuged (13,000 rpm, 5 min) and the supernatant corresponding to the bound protein fraction was subjected to western blotting analysis.

#### Protein immunoprecipitation (IP)

V5-tagged HuR was immunoprecipitated from total protein extracts with anti-V5 antibody covalently crosslinked to a Protein G Sepharose resin to prevent the elution of the antibody with the target protein.^45,66^ On the one hand, Protein A/G PLUS-Agarose (2003, Santa Cruz Biotechnology) was prepared by washing 5 times with 10 column volumes of PBS supplemented with 0.1% sodium azide and centrifugation (5,000 rpm, 5 min, 4°C) to remove the storage solution. Beads were resuspended in 0.1% sodium azide-PBS to maintain the initial slurry volume. For each reaction tube, 100 μL of the Protein A/G PLUS-Agarose slurry were incubated with 2 μg of V5 Tag monoclonal antibody (R960–25, Invitrogen) or Purified Mouse IgG1, κ Isotype Control antibody (557273, BD Pharmingen) in a final volume of 1 mL of 0.1% sodium azide-PBS overnight under rotation at 4°C to enable the binding of the antibody to the resin. Next, beads were centrifuged (2,500 rpm, 5 min, 4°C), washed twice with 1 mL of sodium borate buffer (200 mM boric acid, 3 M NaCl, pH 9.0) and incubated with 1 mL of 50 mM dimethyl pimelimidate dihydrochloride (DMP) (80490, Sigma-Aldrich) dissolved in sodium borate buffer during 30 min under rotation at RT. Beads were centrifuged (2,500 rpm, 5 min, 4°C), washed twice with 1 mL of sodium borate buffer and two more times with 1 mL of 200 mM ethanolamine pH 8.0, before incubation during 2 h under rotation at RT and protected from the light to quench unreacted DMP. Beads were centrifuged (2,500 rpm, 5 min, 4°C), washed twice with 1 mL of PBS, two more times with 1 mL of 200 mM glycine pH 2.5 and two additional times with 1 mL PBS to remove residual non-crosslinked antibody. On the other hand, cells were collected in 50 mM Tris pH 8.5, 150 mM NaCl, 5 mM EDTA, 1% Igepal lysis buffer supplemented with 1 mM phenylmethylsulfonyl fluoride (PMSF), and total protein extraction was performed. Protein concentration of the input fraction was determined by the Micro BCA Protein Assay Kit (23235, Thermo Fisher Scientific), and 500 μg of protein were incubated with the antibody crosslinked to the resin in a final volume of 500 μL of lysis buffer during 2 h under rotation at 4°C. Beads containing the immunoprecipitated proteins were centrifuged (5,000 rpm, 5 min, 4°C) and washed three times with 500 μL of lysis buffer to remove the unbound material. Samples were incubated with 35 μL of 5x Laemmli sample loading buffer 5 min under rotation at RT and boiled at 95°C for 5 min to induce protein denaturation and dissociation from the antibody crosslinked to the resin. Samples were eventually centrifuged (13,000 rpm, 12 min, RT) and the supernatant corresponding to the immunoprecipitated protein or elution fraction was submitted to western blotting analysis.

#### Molecular dynamics (MD) computations

Molecular models were based on the X-ray diffraction (XRD) model of the two N-terminal RRM domains of human HuR at 2 Å resolution (PDB: 4ED5).^83^ SUMO-2 coordinates were taken from the XRD model at 1.6 Å resolution of human SUMO-2, (PDB: 4NPN), which shows 100% identity with the murine form. MD trajectories were computed with the AMBER 16 package,^132^ using the 14SB force field.^143^ Isopeptidic bond parameters were obtained by RHF/6–31G* computations using GAMESS-US.^144^ Hessian matrix in Cartesian coordinates were analyzed with the method developed by Jorge M Seminario,^145^ using the CartHess2FC module of AMBER. Simulations run under periodic boundary conditions in orthorhombic boxes. Initially, the minimum distance between protein and cell faces was 10 Å. Particle mesh Ewald (PME) electrostatics were set with the Ewald summation cut-off at 9 Å. Sodium counter-ions neutralized the charges of the system. The structures were solvated with SPC water molecules. Protein side-chains were energy-minimized (100 steepest descent and 1400 conjugate gradient steps) down to an RMS energy gradient of 0.01 kJ mol^−1^ Å^−1^. Afterward, solvent was subjected to 1000 steps of steepest descent minimization followed by 500 ps NPT-MD computations using isotropic molecule position scaling and a pressure relaxation time of 2 ps at 298K. Temperature was regulated with Berendsen’s heat bath algorithm,^146^ with a coupling time constant equal to 0.5 ps. The density of the system reached a plateau after ca. 150 ps simulation. Then, for each protein, the whole system was energy minimized and submitted to NVT-MD at 298 K, using 2.0 fs integration time steps. Snapshots were saved every 100 ps. The SHAKE algorithm was used to constrain bonds involving hydrogen atoms.^147^ Coordinate files were processed using CPPTRAJ.^148^ Further processing was made in Origin 16 (Originlab) and graphic displays were built in UCSF Chimera (http://www.rbvi.ucsf.edu/chimera). Analyses of domain orientations were performed with ARO script.^149^ The rotation angles of RRM1 relative to RRM2 were determined by the DynDom program.^150,151^

#### Recombinant HuR protein expression and purification

The pGEX-4T2 bacterial expression plasmid coding for HuR RNA-Recognition Motifs (RRMs) 1 and 2 was used as template for site-directed mutagenesis (KRT).^131^ The HuR RRM1–2 tandem constructs (residues 1–189) contained an N-terminal His_6_-tag connected by a short 10 amino acid linker. The HuR RRM1–2 K120R, HuR RRM1–2 K182R and HuR RRM1–2 K120/182R SUMOylation mutants were produced by PCR by using the primers listed in the KRT. Next, *E*. *coli* BL21(DE3) electrocompetent cells were transformed with 100 ng plasmid DNA and cultivated in LB medium supplemented with 100 μg/mL ampicillin. Pre-cultures and cultures were grown at 37°C with a continuous stirring of 150 rpm. Protein overexpression was induced by the addition of 1 mM IPTG once cultures reached an OD_600_ of 0.6. After overnight incubation under continuous stirring at 30°C, cells were collected by centrifugation. Purification by immobilized metal affinity chromatography was performed with a Ni-NTA resin (GE Healthcare), according to the manufacturer’s instructions. The purity of the samples was evaluated by SDS-PAGE.

#### Circular dichroism (CD)

CD spectra were recorded in the far-ultraviolet (UV) range (190–250 nm) at 20°C on a J-815 CD spectropolarimeter (JASCO) equipped with a Peltier temperature control system. 10 μM of each HuR construct was solved into 10 mM sodium phosphate buffer (pH 7.0) and placed into a 1-mm quartz cuvette. The final spectra were an average of 20 scans.

#### Dynamic light scattering (DLS)

DLS experiments were performed in a Zetasizer Nano ZS system (Malvern Instruments). Measurements were carried out at 25°C, using disposable plastic cuvettes with 1 mg/mL of each HuR construct in sodium phosphate buffer (pH 7.0) with 50 mM NaCl and 1 mM TCEP. Intensity auto-correlation functions were analyzed with the Zetasizer software (Malvern Instruments). Volume-weighted Particle Size Distributions (PSDs) were calculated under the assumption of homogeneous particle shape.

#### Isothermal titration calorimetry (ITC)

ITC measurements were carried out at 25°C in a Nano ITC Low Volume calorimeter (TA Instruments). Previously, proteins were dialyzed against 10 mM sodium phosphate buffer (pH 7.0) with 50 mM NaCl and 1 mM TCEP. All solutions were degassed before the titrations were performed. HuR RRM1–2 species at 150–180 μM were injected into the cell containing 10–15 μM of a 11-mer T-rich single-stranded DNA (ssDNA) analog derived from an AU-rich motif of the *c-fos* mRNA (5′-ATTTTTATTTT-3′), purchased from STAB vida. The stirring speed was 300 rpm to ensure cell homogeneity. The reference cell was filled with distilled water. The data corresponding to the heat per injection normalized per mole of injectant versus molar ratio were analyzed with Origin 2018b (OriginLab Corporation) employing a single ligand binding site model. The change of Gibbs free energy and entropy were calculated using the following equations: −*TΔS = ΔG* − D*H* and *ΔG=* −*RT ln*(*K*) at T = 298 K.

#### Brownian dynamics (BD) computations

BD were carried out and analyzed using the SDA-flex 7.1 software package.^152^ The force-field grids used in BD included all electrostatics and desolvation grids.^153,154^ Charges were obtained from PQR files extracted from each MD trajectory. Diffusion constants were computed using the ARO script,^149^ in the Visual Molecular Dynamics (VMD) tcl-tk console.^155^ Electrostatic grids were generated for every conformer with APBS 3.0.^156^ All simulations were carried out at 100 mM ionic strength. In total, 5 structures of HuR RRM1–2 and other 5 of the deca-ribonucleotide were used as input. For k_on_ computations, each of the 5 RRM1–2 molecules were treated as targets in separate computations and set in the coordinate origin, whereas the 5 conformations of the RNA molecule were used as input for Monte-Carlo conformation exchange during the simulation of their diffusion. Conformational exchange was allowed every 2.5 ns. A total of 75,000 diffusion trajectories (5 × 15,000) were then computed for each WT and mutant species. As the k_off_ computations module did not allow conformation exchange, each combination of conformers was treated in a different set of independent trajectories. A total of 62,500 (25 × 2,500) trajectories were computed for each construct. Origin 2018b (OriginLab Corporation) was used for statistical analysis and data representation.

#### Live-cell proliferation and migration imaging

Cells were seeded in tissue-culture treated 96-well plates. For the migration experiment, plates were scratched with a 96-pin WoundMaker (Essen Biosciences) when cells reached 95–100% confluence. Photomicrographs were taken every 2 h using an IncuCyte live-cell analysis system (Essen Biosciences) and confluence of the culture or wound recovery were measured using IncuCyte software (Essen Biosciences) after 132 or 60 h in culture, respectively.

#### Cell invasion assay on a collagen I matrix

The invasive potential was assessed by evaluating cell spheroid growth on a collagen gel.^157^ Cell spheroid formation was achieved by seeding 5,000 cells/well in culture medium with 0.4% methyl cellulose (M0512, Sigma-Aldrich) in non-treated round-bottom 96-well plates (351177, Falcon). After a 3-day incubation, spheroids were individually collected and gently washed with PBS. Each spheroid was embedded in 100 μL of a 1 mg/mL collagen I (354236, Corning) and 7.2 mM NaOH in PBS solution and carefully deposited on tissue-culture treated flat bottom 96-well plates. After incubation at 37°C for 30 min, cell culture medium was added up to a final volume of 200 μL. Pictures of the spheroids were taken after 48 h with an ×10 magnification objective of an Axio Observer Z1 inverted microscope (Zeiss). The invasion ratio was established as the total spheroid surface divided by the spheroid core, which were determined with Fiji software (https://imagej.net/software/fiji/).

#### Annexin V staining

Cells were seeded in tissue-culture treated 6-well plates and allowed to grow until they reached 80% confluency. Apoptotic cells were identified by flow cytometry using the Annexin V FITC Apoptosis detection kit (ANXVKF, Immunostep) in combination with LIVE/DEAD fixable blue dead cell stain kit (L23105, Invitrogen), by following the manufacturer’s instructions. Culture medium was collected, cells were washed with PBS twice, detached by trypsinization and transferred to 15 mL tubes. Each cell pellet was washed with 5 mL of PBS and collected by centrifugation (600 × *g*, 5 min). Each pellet was resuspended in 100 μL of the LIVE/DEAD cell dye in PBS, and incubated 30 min at 4°C in the dark. The staining solution was diluted by adding 500 μL of 5% FBS-PBS per tube and cells were collected by centrifugation (600 × *g*, 5 min). Each cell pellet was resuspended in 100 μL of Annexin V-FITC diluted in Annexin V Binding Buffer solution and incubated 15 min at RT protected from the light. The staining solution was diluted by adding 200 μL of Annexin V Binding Buffer per tube. Annexin V-FITC fluorescence (λ_ex_ = 495 nm, λ_em_ = 519 nm) was acquired in a FACSymphony flow cytometer (BD Biosciences). A set of unstained cells were used as a blank and a group of cells that had been previously treated with 1 μM staurosporin (STS) (S1421, Selleckchem) for 4 h was used as a positive control. The results were analyzed with FlowJo v10 software (BD Biosciences) and the percentage of apoptotic cells was calculated.

#### Crystal violet staining

Cells were seeded in tissue-culture treated 12-well plates. For proliferation studies, time points were collected on day 0 and 3. For colony formation assays, cells were collected after 2 weeks while culture medium was replaced every 3 days. Cell viability was estimated by crystal violet, which is a basic protein dye that binds to ribose-type molecules such as DNA. Cells were washed with PBS twice and fixed in ice-cold 4% paraformaldehyde solution in PBS (sc-281692, Santa Cruz Biotechnology) for 10 min at RT. Cells were washed with PBS twice and incubated with a 0.1% crystal violet (C6158, Sigma-Aldrich) solution in 20% methanol for 40 min at RT with gentle shaking. The staining was discarded and the plates were rinsed with distilled water and air-dried overnight. Crystals were resuspended in 10% acetic acid for 30–60 min at RT with gentle shaking. An approximate volume of 100 μL was transferred into 96-well clear flat bottom plates and absorbance at 595 nm was measured in a SpectraMax M2/M2e microplate reader (Molecular Devices). For higher consistency, replicates of the same well were performed when possible, and 10% acetic acid was used as blank. The absorbance data were used to calculate the percentage of proliferation relative to the initial timepoint and palbociclib IC_50_ values were calculated from the best-fit values of four-parameter dose-response curves with a 95% confidence interval.

#### Caspase-3 activity assay

Cells were seeded in tissue-culture treated 6-well plates and allowed to grow until they reached 80% confluency. Apoptosis was determined by measuring the fluorescence resulting after caspase-3 mediated cleavage of a fluorogenic substrate. In order to recover possible dead cells in suspension, the medium was transferred to a set of tubes and the cell pellet was collected by centrifugation (2,000 rpm, 5 min). Cells were washed with PBS twice and any detached cell was collected by centrifugation (2,000 rpm, 5 min). Cells were lysed in 50 μL of caspase-3 reaction buffer (250 mM PIPES pH 7.4, 100 mM EDTA, 2.5% CHAPS, 125 mM DTT) and combined with the dead cell pellets. Total protein was extracted and protein concentration was determined by the Bradford assay. 40 μg of total protein were added to a mix containing 25 μM Ac-DEVD-AFC caspase-3 fluorogenic substrate (ALX-260–032, Enzo Life Sciences) in reaction buffer, constituting a final volume of 500 μL. Each sample was measured in duplicate by adding 200 μL of the reaction mixture to each well of a 96-well black flat bottom assay plate (3915, Corning). A blank without protein sample was included, and a cell lysate that had been previously treated with 1 μM STS for 4 h was used as a positive control. The reaction plate was incubated at 37°C with gentle shacking for 4 h and fluorescence (λ_ex_ = 390 nm, λ_em_ = 510 nm) was measured every hour in a SpectraMax M2/M2e microplate reader (Molecular Devices). Caspase-3 activity was determined by calculating the increase in fluorescence from 0 to 4 h after background correction, and normalized with total protein.

#### Senescence-associated β-galactosidase (SA β-gal) activity detection

Cells were seeded over 12-mm coverslips (631–1577P, VWR) previously coated with a 0.01% poly-L-lysine solution (P4707, Sigma-Aldrich), in tissue-culture treated 24-well plates and allowed to grow until they reached 50–60% confluency. SA β-gal was assayed with the senescence detection kit (QIA117, Calbiochem), according to the manufacturer’s instructions. Culture medium was removed, cells were washed with PBS once, and incubated with 250 μL of the fixative solution per well, for 10–15 min at RT. The fixative solution was removed, rinsed with PBS twice and cells were incubated with 250 μL of the staining solution mix (12.5 μL of 10 mg/mL X-gal substrate in DMF, 2.5 μL of staining supplement and 235 μL of staining solution) per well, overnight at 37°C. PBS was added to the empty wells in the plate to avoid the evaporation of the staining solution mix. Cells were observed under the microscope for development of blue color, and the reaction was stopped by removal of the staining solution mix and rinsed with PBS three times. Coverslips were mounted in mounting medium (S3023, Agilent) and slides were observed with a Leica DM750 upright brightfield microscope equipped with a Leica ICC50W digital color camera. A minimum of five areas per coverslip were considered so that more than 200 cells per coverslip were manually counted using an ×10 magnification objective. The number of SA β-gal positive cells was normalized by the total number of cells.

#### Tom20 immunofluorescent staining

Mitochondrial network was assessed by Tom20 immunolabeling.^158^ Cells were seeded over 12-mm coverslips (631–1577P, VWR) in tissue-culture treated 24-well plates and allowed to grow until they reached 80% confluency. Cells were permeabilized with 0.1% Triton X-100 in PBS for 15 min and washed with PBS. Blocking was performed in 1% BSA and 2% FBS-PBS during 30 min. Next, cells were incubated with Tom20 antibody (sc-11415, Santa Cruz Biotechnology) diluted 1:200 in blocking solution for 1 h, followed by washes with PBS and incubation with Donkey anti-Rabbit IgG (H + L) Highly Cross-Adsorbed Secondary Antibody, Alexa Fluor 488 (A-21206, Invitrogen) diluted 1:500 in blocking buffer during 30 min. DNA and actin F cytoskeleton were stained with DAPI (D1306, Invitrogen) and Rhodamine-Phalloidin (R415, Invitrogen), respectively. Finally, coverslips were rinsed three times with PBS and mounted using ProLong Gold Antifade Mountant (P10144, Molecular Probes). Images were acquired in a Nikon Ti Eclipse confocal microscope (Nikon Instruments). Hardware and image acquisition were controlled by NIS-Elements imaging software (Nikon Instruments). The pipeline analysis of mitochondrial morphology and mass was adapted in Fiji software from Koopman et al.^91^

#### Transmission electron microscopy (TEM) of epon-embedded ultrathin sections

Cells were seeded in 10 cm tissue-culture treated dishes and allowed to grow until they reached 80–100% confluency. Cells were fixed with equal parts of a 4% glutaraldehyde solution (49625, Sigma-Aldrich) in 0.24 M PBS pH 7.2 and cell culture medium during 2 h at RT. Cells were gently collected with a scraper, transferred to 15 mL tubes and centrifuged (1,000 × *g*, 5 min). The fixative solution was discarded and the pellet was resuspended in 1 mL of 0.12 M PBS pH 7.2 and transferred to a new tube. A compact pellet was generated by centrifugation at 5,000 × *g* 5 min and embedded in epoxy resins. After polymerization, 150 nm thick sections were obtained using an ultramicrotome (Leica Microsystems) and a diamond knife (Diatome), placed on 100 mesh hexagonal Cu/Pd EM grids, stained with uranyl acetate and counterstained with lead citrate to reveal and enhance contrast of cellular membranes. For morphological analysis, ultrathin epon-embedded cell sections were studied by TEM. Images were collected using a JEOL JEM-1230 transmission electron microscope operating at 100 kV and equipped with an Ultrascan 4000S P 4 K × 4 K CCD camera (GATAN). Images were acquired at different magnifications ranging from 1,000× to 20,000X. Analysis and segmentation of mitochondria and ER were performed using images with 2,500X and 5,000× magnification. A total of 56 micrographs per condition were analyzed. Images were first subjected to contrast-limited adaptive histogram equalization (CLAHE) to ensure homogeneity in black and white balance through all micrographs using the Microscopy Image Browser (MIB) standalone version 2.7 software.^159^ Then segmentation masks were manually applied on the cell sites where mitochondria and ER were identified in addition to using local thresholding. Total cell area, total mitochondria area, total ER area, mitochondria major and minor axis, and number of mitochondria per visualized cell were estimated within the region of interest.

#### MitoTracker Green staining

Cells were seeded in tissue-culture treated 6-well plates and allowed to grow until they reached 80% confluency. Mitochondrial mass was assessed by fluorescent labeling with MitoTracker Green FM probe (M7514, Invitrogen) in combination with LIVE/DEAD fixable blue dead cell stain kit (L23105, Invitrogen), by following the manufacturer’s recommendations. Culture medium was removed, cells were washed with PBS twice, detached by trypsinization and transferred to 15 mL tubes. Each cell pellet was washed with 5 mL of PBS and collected by centrifugation (600 × *g*, 5 min). Next, each pellet was resuspended in 100 μL of the LIVE/DEAD cell dye in PBS and incubated for 10 min in the dark at RT. The staining solution was diluted by adding 500 μL of PBS per tube and cells were collected by centrifugation (600 × *g*, 5 min). Each pellet was resuspended in 100 μL of 100 nM MitoTracker Green FM probe in PBS and incubated 45 min at RT protected from the light. The staining solution was diluted by adding 500 μL of PBS per tube and cells were collected by centrifugation (600 × *g*, 5 min). Each cell pellet was resuspended in 200 μL of ice-cold 4% paraformaldehyde solution in PBS (sc-281692, Santa Cruz Biotechnology) and fixed during 15 min in the dark at 4°C. Fixation was stopped by the addition of 1 mL of PBS supplemented with 5% FBS per tube, and cells were collected by centrifugation (600 × *g*, 5 min, 4°C). Each cell pellet was finally resuspended in 200 μL of 5% FBS-PBS. MitoTracker Green FM probe fluorescence (λ_ex_ = 490 nm, λ_em_ = 516 nm) was assayed by flow cytometry in a BD FACSymphony system (BD Biosciences). A set of unstained cells were used as a blank. The results were analyzed with FlowJo v10 software (BD Biosciences) and the geometric mean fluorescence intensity (gMFI) was calculated.

#### Mitochondrial energetic metabolism studies

A two-step seeding process was followed to ensure that cells were evenly distributed throughout the wells of Seahorse XF24 cell culture microplates (102070–001, Agilent). First, 100 μL of the cell suspension were seeded per well and the plates were allowed to rest in the hood for 1 h at RT. In a second step, each well was topped up with 150 μL of culture medium and cells were allowed to grow until they reached 80% confluency. Mitochondrial respiration was assessed by performing a Cell Mito Stress Test in a Seahorse XF24 Analyzer (Agilent). The day before the assay, the XF24 sensor cartridges (102070–001, Agilent) were hydrated with Seahorse XF calibrant solution (102070–001, Agilent) overnight at 37°C in a 0% CO_2_ incubator. On the day of the assay, culture medium was replaced with bicarbonate-free low-buffered assay medium and cells were incubated for 1 h at 37°C in a 0% CO_2_ atmosphere. After establishing a respiration baseline, 6 μM oligomycin, 3 μM carbonyl cyanide 4-trifluoromethoxy-phenylhydrazone (FCCP) and a combination of 0.5 μM antimycin A and 0.5 μM rotenone were sequentially injected through the reagent ports of the cartridges in order to measure changes in the oxygen consumption rate (OCR) related to ATP-linked, maximum and non-mitochondrial respiration, respectively. The OCR, expressed as pmol of O_2_/min, was normalized by the number of cells or protein content, which were estimated by crystal violet or Micro BCA Protein Assay Kit, respectively.

#### MitoSOX red staining

Cells were seeded over 12-mm coverslips (631–1577P, VWR) previously coated with a 0.01% poly-L-lysine solution (P4707, Sigma-Aldrich), in tissue-culture treated 24-well plates and allowed to grow until they reached 80% confluency. Mitochondrial reactive oxygen species (ROS) production was measured by MitoSOX Red mitochondrial superoxide indicator (M36008, Invitrogen), according to the manufacturer’s instructions. MitoSOX Red is a fluorogenic dye which is selectively targeted to mitochondria and exhibits red fluorescence when oxidized by superoxide anion. Cells were washed twice with warm DPBS, calcium, magnesium (14040133, Invitrogen) and incubated with 500 μL of1 μM MitoSOX Red reagent diluted in DPBS, calcium, magnesium for 10 min at 37°C protected from the light. An unstained well was used as blank. Cells were washed with DPBS, calcium, magnesium three times and fixed in ice-cold 4% paraformaldehyde solution in PBS (sc-281692, Santa Cruz Biotechnology) for 10 min at RT. The fixative solution was removed after two washes with DPBS, calcium, magnesium and coverslips were mounted in Fluoroshield with DAPI mounting medium (F6057, Sigma-Aldrich). MitoSOX Red fluorescence (λ_ex_ = 510 nm, λ_em_ = 580 nm) was observed in the Axio Imager D1 epifluorescent microscope (Zeiss). A minimum of five areas per coverslip were assessed using an ×40 objective. MitoSOX Red fluorescence intensity was quantified using Fiji software (https://imagej.net/software/fiji/) and normalized by the number of nuclei stained with DAPI.

#### Tetramethylrhodamine (TMRE) staining

Cells were seeded in tissue-culture treated 24-well plates and allowed to grow until they reached 80% confluency. Mitochondrial membrane potential was estimated by Tetramethylrhodamine, Ethyl Ester, Perchlorate (TMRE) (T669, Invitrogen), by following the manufacturer’s indications. TMRE is a fluorogenic cationic dye which is selectively targeted to active mitochondria. Cells were washed twice with warm DPBS, calcium, magnesium (14040133, Invitrogen) and incubated with 500 μL of 0.5 μM TMRE probe diluted in DPBS, calcium, magnesium for 30 min at 37°C protected from the light. An unstained well was used as blank. Cells were washed with DPBS, calcium, magnesium twice and maintained in 300 μL DPBS, calcium, magnesium. TMRE fluorescence (λ_ex_ = 548 nm, λ_em_ = 574 nm) was measured in a SpectraMax M2/M2e microplate reader (Molecular Devices). TMRE probe fluorescence was normalized with the total protein content of each well, which was determined by the Micro BCA Protein Assay Kit.

#### ATP levels quantification

Cells were seeded in tissue-culture treated 12-well plates and allowed to grow until they reached 80% confluency. Cellular ATP levels were determined with the ATPlite luminescence assay system (6016943, PerkinElmer), by following the manufacturer’s instructions. The assay was performed in OptiPlate-96 white opaque 96-well microplates (6005290, PerkinElmer) and luminescence was measured in a Veritas microplate luminometer (Turner BioSystems). ATP concentration was calculated by interpolation to an ATP standard curve and subsequent normalization with the total protein content of each sample, which was determined by the Bradford assay.

#### NAD^+^/NADH measurement

Cells were seeded in tissue-culture treated 6-well plates and allowed to grow until they reached 80% confluency. Cellular NAD^+^/NADH levels were determined with the NAD/NADH Colorimetric Assay Kit (ab65348, Abcam), according to the manufacturer’s instructions. The assay was performed in 96-well clear flat bottom plates and absorbance at 450 nm was measured in a SpectraMax M2/M2e microplate reader (Molecular Devices) after 2 h incubation at RT with NAD cycling enzyme mix. Total NAD and NADH concentrations were calculated by interpolation to a NADH standard curve. The levels of NAD^+^ were calculated by subtracting NADH from total NAD, and the NAD^+^/NADH were represented.

#### Cytosolic calcium (Ca^2+^) concentration determination

Cells were seeded over 12-mm coverslips (631–1577P, VWR) previously coated with a 0.01% poly-L-lysine solution (P4707, Sigma-Aldrich), in tissue-culture treated 24-well plates and allowed to grow until they reached 80% confluency. Cytosolic Ca^2+^ levels were determined by using Fura-2-AM (F1201, Invitrogen).^160,161^ Fura-2 is a cell-permeable ratiometric cytosolic Ca^2+^ indicator, whose excitation wavelength shifts from 380 nm to 340 nm as it binds to Ca^2+^, while its emission maximum is independent of Ca^2+^ concentration. Cells were washed twice with 0% FBS-culture medium and incubated with 1 μM Fura-2 dissolved in 0% FBS-culture medium for 30–45 min at 37°C. After loading, cells were incubated in 0% FBS-culture medium for 15 min at 37°C protected from the light. Coverslips were washed with 20 mM Tris-HCl pH 7.4, 2.4 mM CaCl_2_, 10 mM glucose solution and mounted on a thermostatized micro-perfusion chamber. Single-cell Fura-2 excitation intensity ratio (λ_ex_ = 340 and 380 nm, λ_em_ = 510 nm) was measured with an ×40 oil-immersion magnification objective in an Eclipse TE 300-based microspectrofluorometer (Nikon) coupled to a DeltaRAM illumination system (Photon Technologies International). After recording a baseline for 30 s, 10 μM thapsigargin (T9033, Sigma-Aldrich) or 100 nM ATP was added to the medium to trigger the release of Ca^2+^ from the endoplasmic reticulum (ER) for 2 min and finally Ca^2+^ was added to measure the entrance of extracellular Ca^2+^ for additional 2 min. The ratio of excitation intensities at 340 nm and 380 nm (R) was related to Ca^2+^ levels by the following equation: [*Ca*^*2*+^]*= K*_*d*_ *× Q ×* (*R R*_*min*_)*/*(*R*_*max*_ *R*). R_min_ and R_max_ refer to the fluorescence intensity ratios when the probe is free or completely saturated of Ca^2+^ respectively, Q represents the ratio of minimum to maximum fluorescence at 380 nm (F_min_/F_max_), and K_d_ is the Ca^2+^ dissociation constant of Fura-2. The values for F_min_, F_max_, R_min_, R_max_ and K_d_ were previously determined by means of a Fura-2 calibration curve in the presence of known Ca^2+^ concentrations.

### QUANTIFICATION AND STATISTICAL ANALYSIS

Statistical analysis was performed using Prism 8 software (GraphPad). Statistical details of experiments can be found in the figures and corresponding legends. Unless otherwise stated, experiments were reproduced at least three times. Data are represented as the mean ± standard deviation (SD) of at least three biological replicates within one representative experiment. A two-tailed t test was used to compare the differences between two groups. A p value <0.05 was considered statistically significant for all analyses and defined as *p < 0.05, **p < 0.01, ***p < 0.001 and ****p < 0.0001. If not indicated otherwise, the differences were not significant (n.s.).

### ADDITIONAL RESOURCES

#### Animations

Animations were created using BioRender software (https://biorender.com).

## Supplementary Material

1

## Figures and Tables

**KEY RESOURCES TABLE:** 

REAGENT or RESOURCE	SOURCE	IDENTIFIER

Antibodies		

HuR/ELAV1 (3A2)	Santa Cruz Biotechnology	Cat#sc-5261; RRID:AB_627770
SUMO1 76–86	Developmental Studies Hybridoma Bank (DSHB)	N/A
SUMO-2 8A2	Developmental Studies Hybridoma Bank (DSHB)	N/A
UBC9	Proteintech	Cat#14837-1-AP; RRID:AB_2272479
V5 tag	Invitrogen	Cat#R960-25; RRID: N/A
His	Cytiva	Cat#27-4710-01; RRID:AB_771435
FLAG M2	Sigma-Aldrich	Cat#F1804; RRID:AB_262044
RGSHis	Qiagen	Cat#34610; RRID: N/A
Cyclin D1 (92G2)	Cell Signaling Technology	Cat#2978; RRID:AB_2259616
Cyclin A2 (BF683)	Cell Signaling Technology	Cat#4656; RRID:AB_2071958
Phospho-Rb (Ser780) (D59B7)	Cell Signaling Technology	Cat#8180; RRID:AB_10950972
Rb (4H1)	Cell Signaling Technology	Cat#9309; RRID:AB_823629
gamma H2A.X (phospho S139) [EP854(2)Y]	Abcam	Cat#ab81299; RRID:AB_1640564
β-Actin	Sigma-Aldrich	Cat#A5441; RRID:AB_476744
Anti-mouse IgG, HRP-linked	Cell Signaling Technology	Cat#7076; RRID:AB_330924
Anti-rabbit IgG, HRP-linked	Cell Signaling Technology	Cat#7074; RRID:AB_2099233

Biological samples		

Human HCC tumor and surrounding non-tumor tissue	Córdoba Node of the Andalusian Public Health System Biobank	http://www.biobancosspa.com
Human HCC tumor and surrounding non-tumor tissue	Basque Biobank	https://www.biobancovasco.org
*MYC;Trp53^−/−^* mouse liver tissue	Amaia Lujambio^74^	N/A

Chemicals, peptides, and recombinant proteins		

Geneticin (G418 sulfate) selective antibiotic	Gibco	Cat#11811031
ML-792 SUMO-Activating Enzyme inhibitor	MedChemExpress	Cat#HY-108702
Staurosporin (STS)	Selleckchem	Cat#S1421
Palbociclib, isethionate salt, >99%	LC Laboratories	Cat#P-7766
Glutathione-agarose, lyophilized powder	Sigma-Aldrich	Cat#G4510
Low density nickel-agarose beads	ABT	Cat#6BCL-QLNi-25
Protein A/G PLUS-Agarose	Santa Cruz Biotechnology	Cat#2003
Protein G Sepharose 4 Fast Flow	Cytiva	Cat#GE17-0618-01
PR-619 DUB inhibitor V	Calbiochem	Cat#662141
Complete mini EDTA-free protease inhibitor cocktail tablets	Roche	Cat#11836170001

Critical commercial assays		

QuickChange site-directed mutagenesis kit	Stratagene	Cat#200518
Lipofectamine 2000 transfection reagent	Invitrogen	Cat#11668019
DharmaFECT 1 transfection reagent	Horizon Discovery	Cat#T-2001-03
Maxwell 16 LEV RNA FFPE purification kit	Promega	Cat#AS1260
SYBR Select master mix	Invitrogen	Cat#4472908
TruSeq Stranded Total RNA human/mouse/rrat kit	Illumina	Cat#RS-122-2201
Annexin V FITC Apoptosis detection kit	Immunostep	Cat#ANXVKF
LIVE/DEAD fixable blue dead cell stain kit	Invitrogen	Cat#L23105
Ac-DEVD-AFC caspase-3 fluorogenic substrate	Enzo Life Sciences	Cat#ALX-260-032
Senescence detection kit	Calbiochem	Cat#QIA117
MitoTracker Green FM dye	Invitrogen	Cat#M7514
MitoSOX Red mitochondrial superoxide indicator	Invitrogen	Cat#M36008
Tetramethylrhodamine, ethyl ester, perchlorate (TMRE)	Invitrogen	Cat#T669
ATPlite luminescence assay system	Perkin Elmer	Cat#6016943
NAD/NADH colorimetric assay kit	Abcam	Cat#ab65348
Fura-2, AM, cell permeant	Invitrogen	Cat#F1201

Deposited data		

RIP-Seq	This paper	GEO: GSE197798

Experimental models: Cell lines		

Human: THLE-2 cells	ATCC	CRL-2706
Human: PLC/PRF/5 cells	ATCC	CRL-8024
Human: HuH-7 cells	JCRB Cell Bank	JCRB0403
Human: HuH-7 ^WT HuR^ cells	This paper	N/A
Human: HuH-7 ^HuR [K120R]^ cells	This paper	N/A
Human: HuH-7 ^HuR [K182R]^ cells	This paper	N/A
Human: HuH-7 ^HuR [K120/182R]^ cells	This paper	N/A
Mouse: MLP-29 cells	Enzo Medico^119^	N/A

Experimental models: Organisms/strains		

Mouse: NOD SCID gamma	Charles River Laboratories	Strain code: 614
Oligonucleotides		
Primers for qPCR, See Table S2	This paper	N/A
siRNA targeting sequence: Mouse *Pias2b* Sense: 5’- CCUCCUAUGUUUUUGGAUAtt-3’ Antisense: 5’- UAUCCAAAAACAUAGGAGGac-3’	Thermo Fisher Scientific	Custom
siRNA targeting sequence: Mouse *Senp1* Sense: 5’- AGAAAGGUGGGUUAACAAAtt-3’ Antisense: 5’- UUUGUUAACCCACCUUUCUca-3’	Thermo Fisher Scientific	Cat#n383576
siRNA targeting sequence: Mouse *Senp2* Sense: 5’- GAUUAGGUACUACAUCUUUtt-3’ Antisense: 5’- AAAGAUGUAGUACCUAAUCtt-3’	Thermo Fisher Scientific	Cat#n251444
siRNA targeting sequence: Mouse *Senp3* Sense: 5’- CCGACCCUCUCAUAGAAAAtt-3’ Antisense: 5’- UUUUCUAUGAGAGGGUCGGag-3’	Thermo Fisher Scientific	Cat#s96092
Primer: HuR RRM1-2 K120R Forward: 5’- ACCATGACCCAGAGGGACGTAGAAGAC-3’	STAB vida	Custom
Primer: HuR RRM1-2 K120R Reverse: 5’ - GTCTTCTACGTCCCTCTGGGTCATGGT-3’	STAB vida	Custom
Primer: HuR RRM1-2 K182R Forward: 5’- CATCACAGTGAGGTTTGCAGCCAA-3’	STAB vida	Custom
Primer: HuR RRM1-2 K182R Reverse: 5’- GTTGGCTGCAAACCTCACTGTGATG-3’	STAB vida	Custom

Recombinant DNA		

Plasmid: pcDNA3-His_6_-Ub	Manuel S Rodríguez^120^	N/A
Plasmid: pcDNA3-His_6_-SUMO-1	Manuel S Rodríguez^121,122^	N/A
Plasmid: pcDNA3-His_6_-SUMO-2	Manuel S Rodríguez^121,122^	N/A
Plasmid: pcDNA3-His_6_-SUMO-3	Manuel S Rodríguez^121,122^	N/A
Plasmid: pGEX-2T-Ubc9	Manuel S Rodríguez^123^	N/A
Plasmid: pCMV-FLAG-hPIAS1	Manuel S Rodríguez^124^	N/A
Plasmid: pCMV-FLAG-hPIASxα	Arora et al^125^	Addgene #15209
Plasmid: pCMV-FLAG-hPIASxβ	Arora et al.^125^	Addgene #15210
Plasmid: pCMV-FLAG-hPIASγ	Liu etal.^126^	Addgene #15208
Plasmid: FLAG-hSENPI	Manuel S Rodríguez^124^	N/A
Plasmid: pFLAG-CMV-hSENP2	Kang et al.^127^	Addgene #18047
Plasmid: pcDNA3-RGS-hSENP3	Gong et al.^128^	Addgene #18048
Plasmid: pcDNA3-RGS-hSENP5	Gong et al.^128^	Addgene #18053
Plasmid: pFLAG-CMV-hSENP6	Dou etal.^129^	Addgene #18065
Plasmid: p3xFLAG-CMV-10-hSENP7	Bawa-Khalfe et al.^130^	Addgene #42886
Plasmid: pcDNA3.3-TOPO	Invitrogen	Cat# K830001
Plasmid: pcDNA3.3-TOPO-V5-mHuR WT	This paper	N/A
Plasmid: pcDNA3.3-TOPO-V5-mHuR [K50R]	This paper	N/A
Plasmid: pcDNA3.3-TOPO-V5-mHuR [K72R]	This paper	N/A
Plasmid: pcDNA3.3-TOPO-V5-mHuR[ K89R]	This paper	N/A
Plasmid: pcDNA3.3-TOPO-V5-mHuR[ K92R]	This paper	N/A
Plasmid: pcDNA3.3-TOPO-V5-mHuR [K104R]	This paper	N/A
Plasmid: pcDNA3.3-TOPO-V5-mHuR [K120R]	This paper	N/A
Plasmid: pcDNA3.3-TOPO-V5-mHuR [K156R]	This paper	N/A
Plasmid: pcDNA3.3-TOPO-V5-mHuR [K182R]	This paper	N/A
Plasmid: pcDNA3.3-TOPO-V5-mHuR [K191R]	This paper	N/A
Plasmid: pcDNA3.3-TOPO-V5-mHuR [K120/182R]	This paper	N/A
Plasmid: pGEX-4T2-His_6_-HuR RRM1-2 WT	Irene Diaz-Moreno^131^	N/A
Plasmid: pGEX-4T2-His_6_-HuR RRM1-2 [K120R]	This paper	N/A
Plasmid: pGEX-4T2-His_6_-HuR RRM1-2 [K182R]	This paper	N/A
Plasmid: pGEX-4T2-His_6_-HuR RRM1-2 [K120/182R]	This paper	N/A

Software and algorithms		

The Cancer Genome Atlas (TCGA)	National Cancer Institute	https://www.cancer.gov/tcga
The Gene Set Enrichment Analysis (GSEA)	UC San Diego and Broad Institute	https://www.gsea-msigdb.org/gsea/
Ingenuity Pathway Analysis (IPA)	Qiagen	https://digitalinsights.qiagen.com/
PEAKS Studio	Bioinformatics Solutions	https://www.bioinfor.com/peaks-studio/
Perseus	Max Planck Institute of Biochemistry	https://www.maxquant.org/perseus/
The Amber 16 Molecular Dynamics Package	Case et al.^132^	https://ambermd.org/GetAmber.php
Origin 2018b	OriginLab	https://www.originlab.com/2018b
Chimera	UC San Francisco	http://www.rbvi.ucsf.edu/chimera
FlowJo version 10	BD Biosciences	https://www.flowjo.com/solutions/flowjo/
Microscopy Image Browser (MIB) version 2.7	University of Helsinki	https://mib.helsinki.fi/
Agilent Seahorse Analytics	Agilent	https://seahorseanalytics.agilent.com/
ImageJ	National Institutes of Health	https://imagej.net/software/fiji/
Prism version 8	GraphPad	https://www.graphpad.com/
BioRender	Science Suite Inc.	https://www.biorender.com

## Highlights

- HuR is SUMOylated in preclinical and clinical hepatocellular carcinoma tumors
- SUMOylation affects HuR binding affinity to its target RNAs
- HuR SUMOylation promotes major cancer hallmarks, namely proliferation and invasion
- Lack of HuR SUMOylation results in a senescent phenotype
