# Supplementary material for: SUMOylation controls Hu antigen R posttranscriptional activity in liver cancer
Source: Cell Rep. Author manuscript; Available in PMC 2024 Apr 18. (PMC11025316; doi:10.1016/j.celrep.2024.113924)
Supplement: 1 [file NIHMS1980936-supplement-1.pdf]

Cell Reports, Volume *43*

Supplemental information

SUMOylation controls Hu antigen R posttranscriptional activity in liver cancer

Sofia Lachiondo-Ortega, Claudia M. Rejano-Gordillo, Jorge Simon, Fernando Lopitz-Otsoa, Teresa C. Delgado, Krystyna Mazan-Mamczarz, Naroa Goikoetxea-Usandizaga, L. Estefanía Zapata-Pavas, Ana García-del Río, Pietro Guerra, Patricia Peña-Sanfélix, Natalia Hermán-Sánchez, Ruba Al-Abdulla, Carmen Fernandez-Rodríguez, Mikel Azkargorta, Alejandro Velázquez-Cruz, Joris Guyon, César Martín, Juan Diego Zalamea, Leire Egia-Mendikute, Arantza Sanz-Parra, Marina Serrano-Maciá, Irene González-Recio, Monika Gonzalez-Lopez, Luis Alfonso Martínez-Cruz, Patrizia Pontisso, Ana M. Aransay, Rosa Barrio, James D. Sutherland, Nicola G.A. Abrescia, Félix Elortza, Amaia Lujambio, Jesus M. Banales, Raúl M. Luque, Manuel D. Gahete, Asís Palazón, Matias A. Avila, Jose J. G. Marin, Supriyo De, Thomas Daubon, Antonio Díaz-Quintana, Irene Díaz-Moreno, Myriam Gorospe, Manuel S. Rodríguez, and María Luz Martínez-Chantar

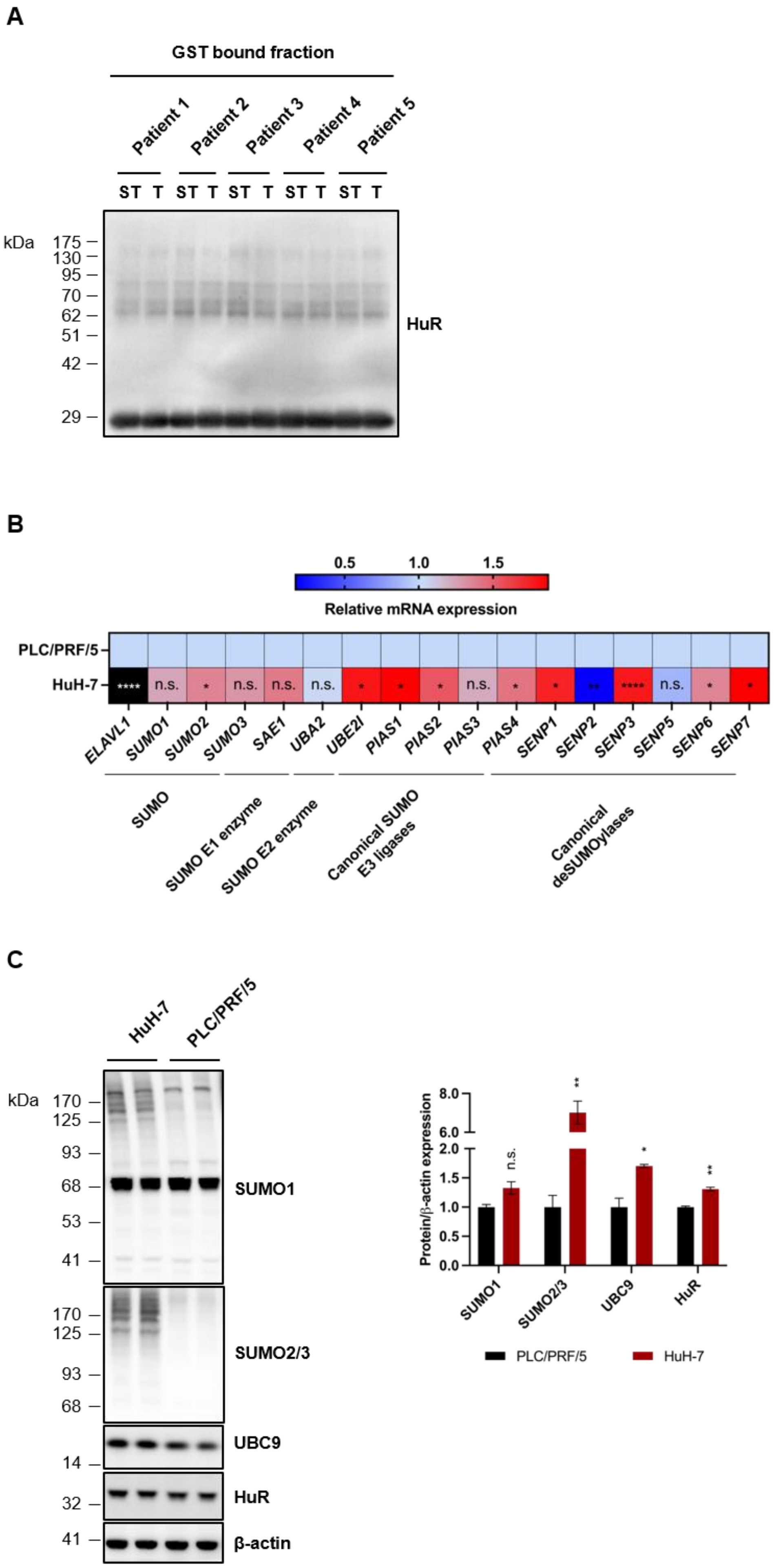
A
GST bound fraction
Patient 1
Patient 2
Patient 3
Patient 4
Patient 5
ST T ST T ST T ST T ST T
kDa
175
130
95
70
62
51
42
29
HuR
B
0.5
1.0
1.5
Relative mRNA expression
PLC/PRF/5
HuH-7
**** n.s. * n.s. n.s. n.s. * * * n.s. * * ** **** n.s. * *
ELAVL1
SUMO1
SUMO2
SUMO3
SAE1
UBA2
UBE2I
PIAS1
PIAS2
PIAS3
PIAS4
SENP1
SENP2
SENP3
SENP5
SENP6
SENP7
SUMO
SUMO E1 enzyme
SUMO E2 enzyme
Canonical SUMO E3 ligases
Canonical deSUMOylases
C
HuH-7
PLC/PRF/5
kDa
170
125
93
68
53
41
SUMO1
170
125
93
68
SUMO2/3
UBC9
14
HuR
32
β-actin
41
Protein/β-actin expression
8.0
6.0
2.0
1.5
1.0
0.5
0.0
n.s.
**
*
**
SUMO1
SUMO2/3
UBC9
HuR
PLC/PRF/5
HuH-7

Figure S1. (A) Detection of SUMOylated HuR in the tumor (T) and surrounding tissue (ST) of a cohort of HCC patients (n=5) after protein pulldown with GST control and western blotting analysis. ELAVL1/HuR and the main components of the SUMO pathway **(B)** mRNA and **(C)** protein expression levels and quantification of in the PLC/PRF/5 and HuH-7 human hepatoma cell lines.

(C) Data are represented as the mean ± SD of at least three biological replicates within one representative experiment. *$p < 0.05$, **$p < 0.01$, ***$p < 0.001$ and ****$p < 0.0001$, two-tailed t-test. If not indicated otherwise, the differences were n.s. Western blots are representative of at least three biological replicates.

Related to **Figure 1**.

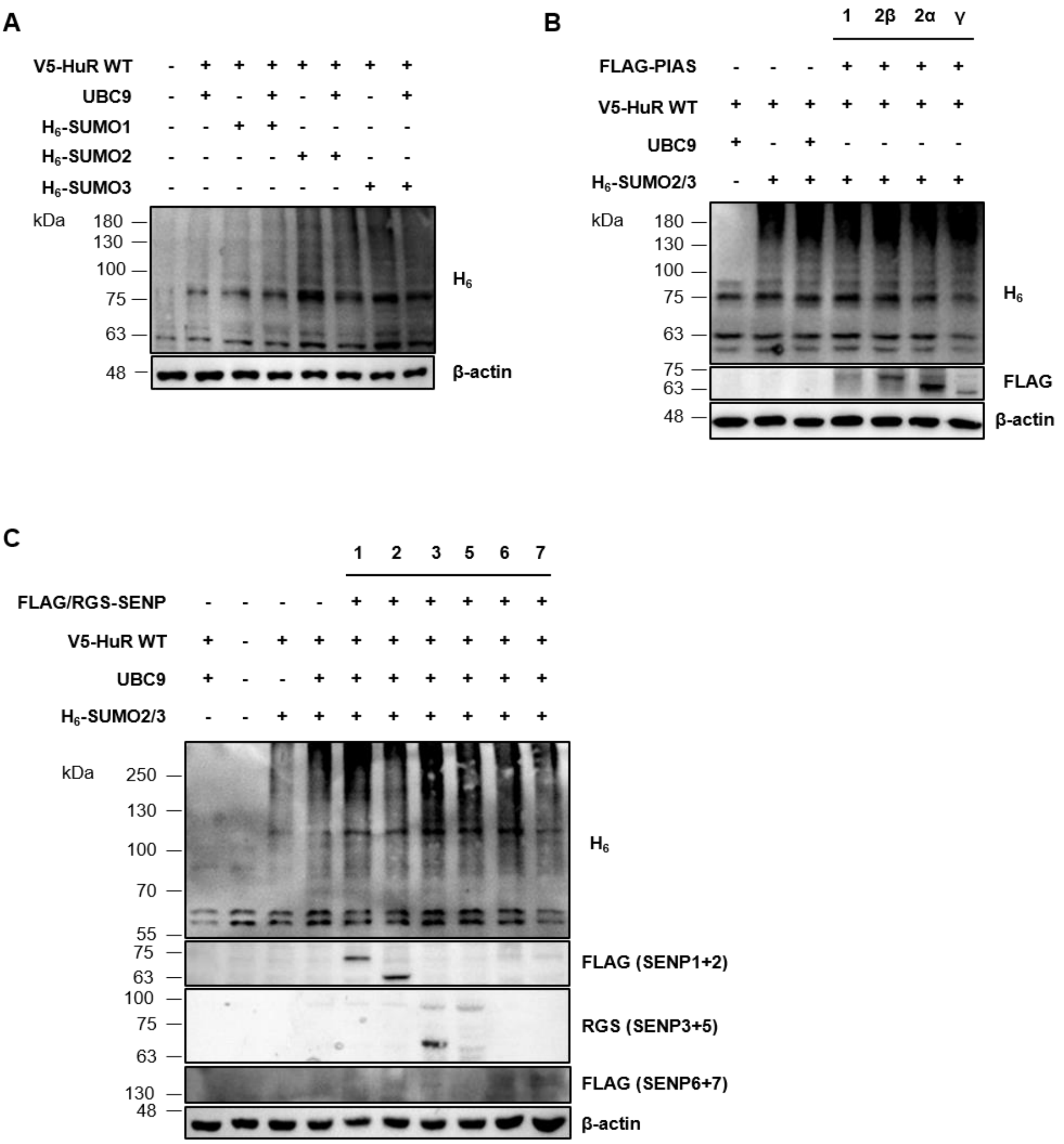

Figure S2. H_6-tagged SUMO and FLAG/RGS-tagged PIAS and SENPs protein expression levels after transient transfection of plasmids expressing **(A)** SUMO, **(B)** PIAS and **(C)** SENP isoforms, in addition to WT HuR and UBC9, in the mouse liver progenitor MLP-29 cell line.

(A-C) Western blots are representative of at least three biological replicates.

Related to **Figure 2**.

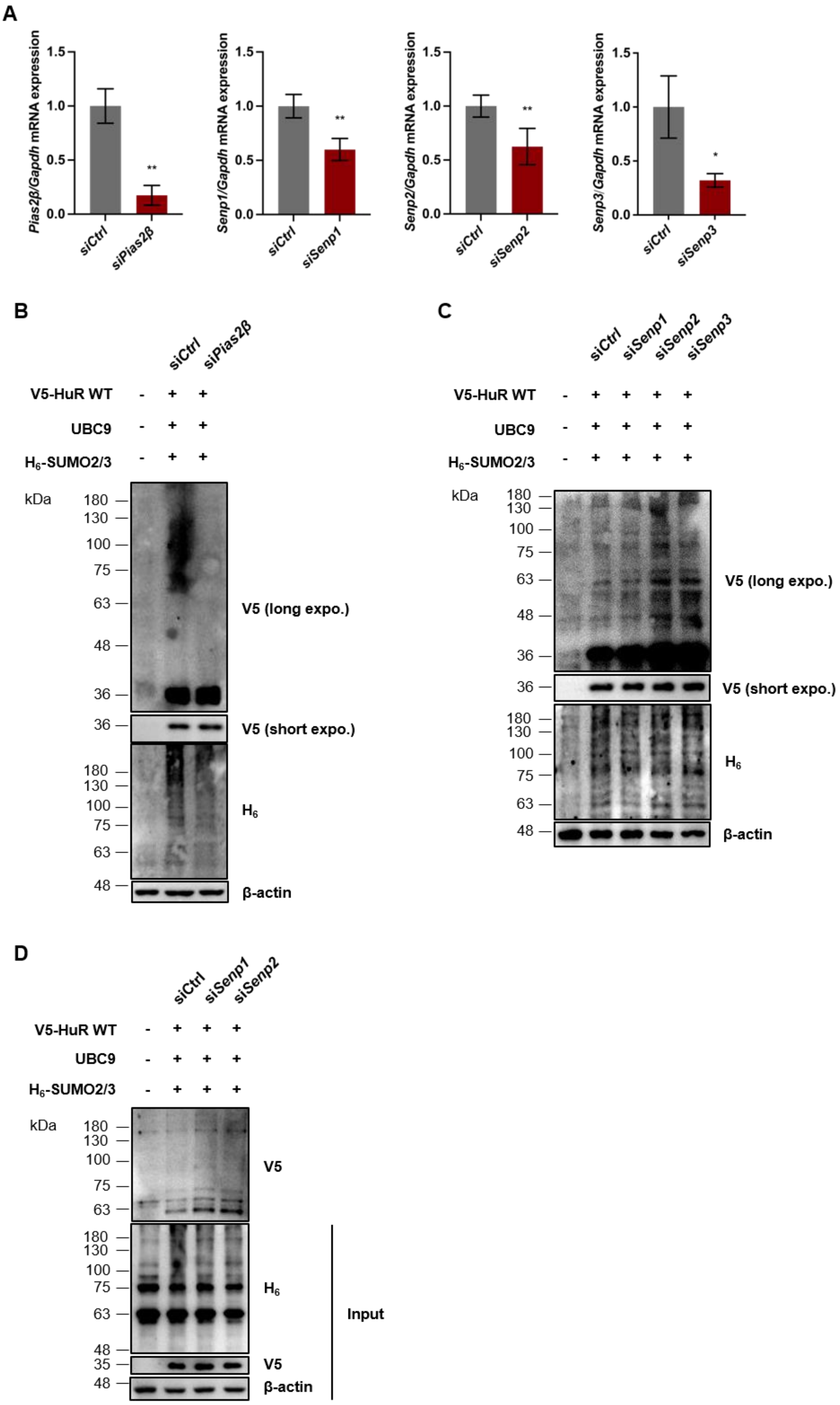
A
Pias2β/Gapdh mRNA expression
siCtrl
siPias2β
Senp1/Gapdh mRNA expression
siSenp1
Senp2/Gapdh mRNA expression
siSenp2
Senp3/Gapdh mRNA expression
siSenp3
1.5
1.0
0.5
0.0
**
*
B
C
D
V5-HuR WT
UBC9
H6-SUMO2/3
kDa
180
130
100
75
63
48
36
35
V5 (long expo.)
V5 (short expo.)
H6
β-actin
V5
Input

Figure S3. **(A)** *Pias2b*, *Senp 1*, *2* and *3* mRNA expression levels after siRNA transfection in the MLP-29 cell line. V5-tagged HuR protein expression levels and its smear after co-transfection of plasmids expressing WT HuR, UBC9 and SUMO2/3 in addition to siRNAs silencing a subset of **(B)** *Pias* and **(C)** *Senp* isoforms in the MLP-29 cell line. **(D)** Modified V5-HuR protein enrichment after transient transfection of plasmids inducing the expression of WT HuR, UBC9 and SUMO2/3 in addition to siRNAs downregulating *Senp1* or *Senp2* expression and subsequent nickel-histidine affinity purification, relative to total V5-HuR protein expression levels in the MLP-29 cell line.

(A) Data are represented as the mean ± SD of at least three biological replicates within one representative experiment. *$p < 0.05$ and **$p < 0.01$, two-tailed t-test.

(B-D) Western blots are representative of at least three biological replicates.

Related to **Figure 2**.

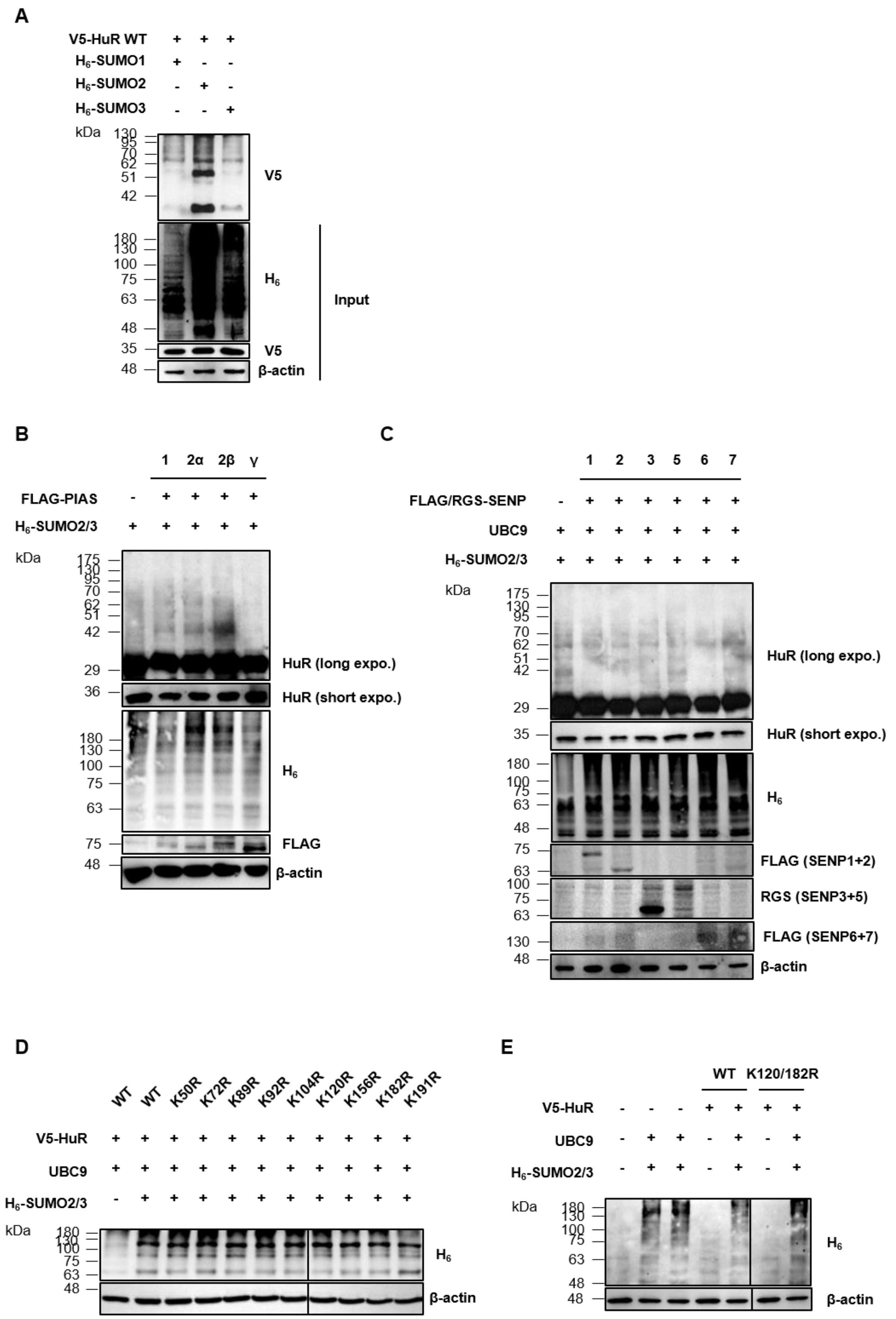

Figure S4. (A) Modified V5-HuR protein enrichment after transient transfection of plasmids expressing the different SUMO paralogs and subsequent nickel-histidine affinity purification, relative to total V5-HuR protein expression levels in the HuH-7

human hepatoma cell line. HuR protein expression levels and its smear after co-transfection of plasmids expressing SUMO2/3 as well as the **(B)** PIAS and **(C)** SENP isoforms in the HuH-7 cell line. H_6-tagged SUMO2/3 and UBC9 protein expression levels after transient transfection of plasmids expressing UBC9, SUMO2/3 and WT HuR or a subset of lysine-to-arginine HuR mutants contained in the RRM1-2 domains in the **(D)** MLP-29 and **(E)** HuH-7 cell lines.
(A-E) Western blots are representative of at least three biological replicates.
(D and E) The entire blot image was digitally processed to eliminate irrelevant lanes.
Related to **Figure 2**.

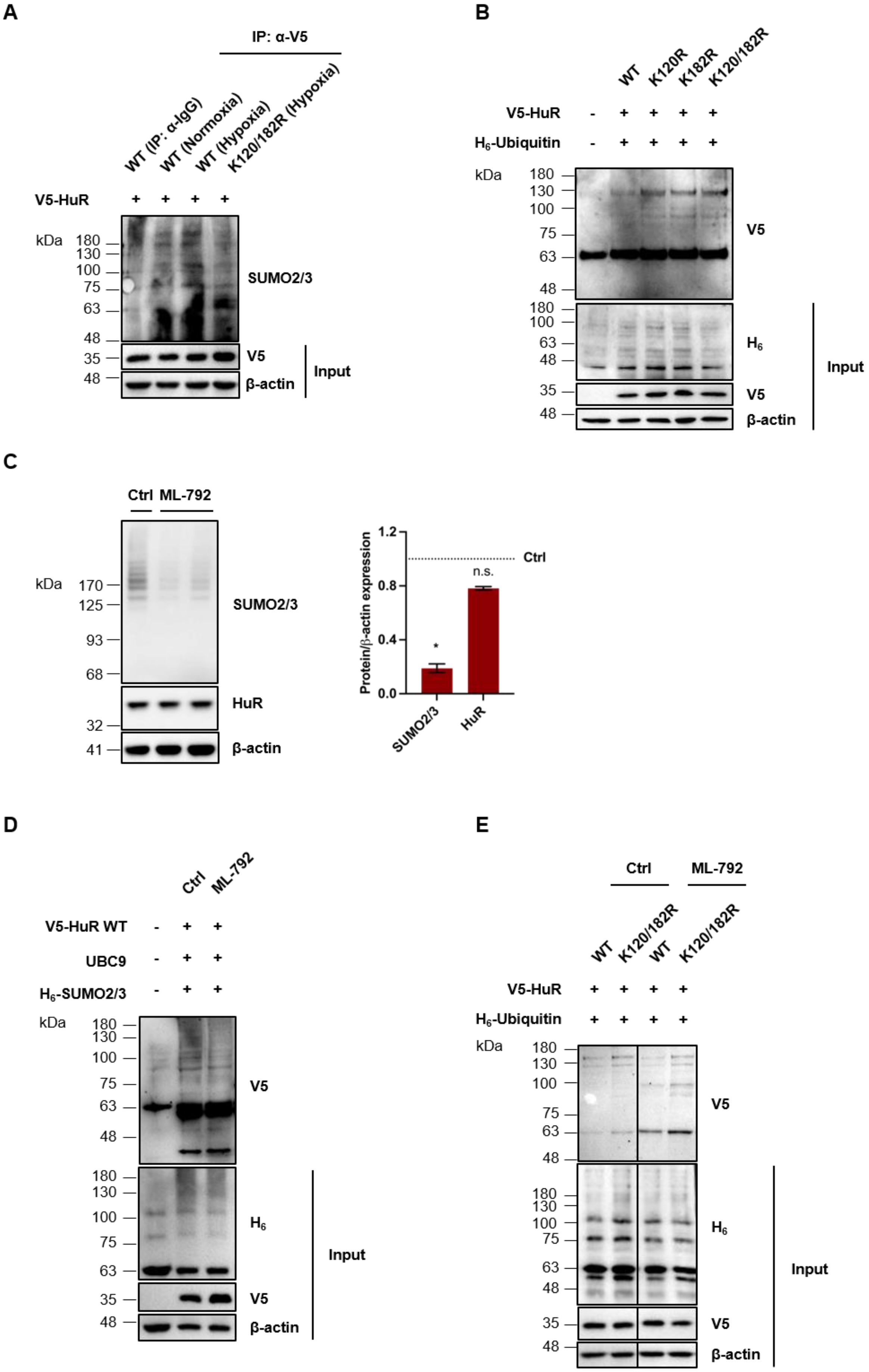
A
IP: α-V5
WT (IP: α-IgG)
WT (Normoxia)
WT (Hypoxia)
K120/182R (Hypoxia)
V5-HuR + + + +
kDa
180
130
100
75
63
48
35
48
SUMO2/3
V5
β-actin
Input
B
WT
K120R
K182R
K120/182R
V5-HuR - + + + +
H6-Ubiquitin - + + + +
kDa
180
130
100
75
63
48
180
100
63
48
35
48
V5
H6
Input
V5
β-actin
C
Ctrl
ML-792
kDa
170
125
93
68
32
41
SUMO2/3
HuR
β-actin
Protein/β-actin expression
1.2
0.8
0.4
0.0
Ctrl
n.s.
*
SUMO2/3
HuR
D
Ctrl
ML-792
V5-HuR WT - + +
UBC9 - + +
H6-SUMO2/3 - + +
kDa
180
130
100
75
63
48
180
130
100
75
63
35
48
V5
H6
Input
V5
β-actin
E
Ctrl
ML-792
WT
K120/182R
WT
K120/182R
V5-HuR + + + +
H6-Ubiquitin + + + +
kDa
180
130
100
75
63
48
180
130
100
75
63
48
35
48
V5
H6
Input
V5
β-actin

Figure S5. (A) SUMO2/3-modified V5-HuR enrichment after transient transfection of plasmids expressing WT and K120/182R HuR under normoxic and hypoxic conditions and subsequent protein immunoprecipitation using anti-V5 antibody, relative to total V5-HuR protein expression levels in the HuH-7 human hepatoma cell line. **(B)** Ubiquitinated V5-HuR protein enrichment after transient transfection of plasmids inducing the expression of WT HuR and the different SUMOylation mutants as well as H_6-tagged ubiquitin and downstream nickel-histidine affinity purification, relative to total V5-HuR protein expression levels in the HuH-7 cell line. **(C)** SUMO2/3 and HuR protein expression levels and quantification after incubating the HuH-7 cell line with 100 nM ML-792 SAE inhibitor for 4 h. **(D)** SUMO2/3-modified and **(E)** ubiquitinated V5-HuR enrichment after transient transfection of plasmids expressing HuR and H_6-tagged SUMO2/3 or ubiquitin, respectively, in addition to treatment with 100 nM ML-792 for 4 h and subsequent nickel-histidine affinity purification, relative to total V5-HuR protein expression levels in the HuH-7 human hepatoma cell line.

(A-E) Western blots are representative of at least three biological replicates. **(E)** The entire blot image was digitally processed to eliminate irrelevant lanes.

(C) Data are represented as the mean ± SD of at least three biological replicates within one representative experiment. *$p < 0.05$, two-tailed t-test.

Related to **Figure 2**.

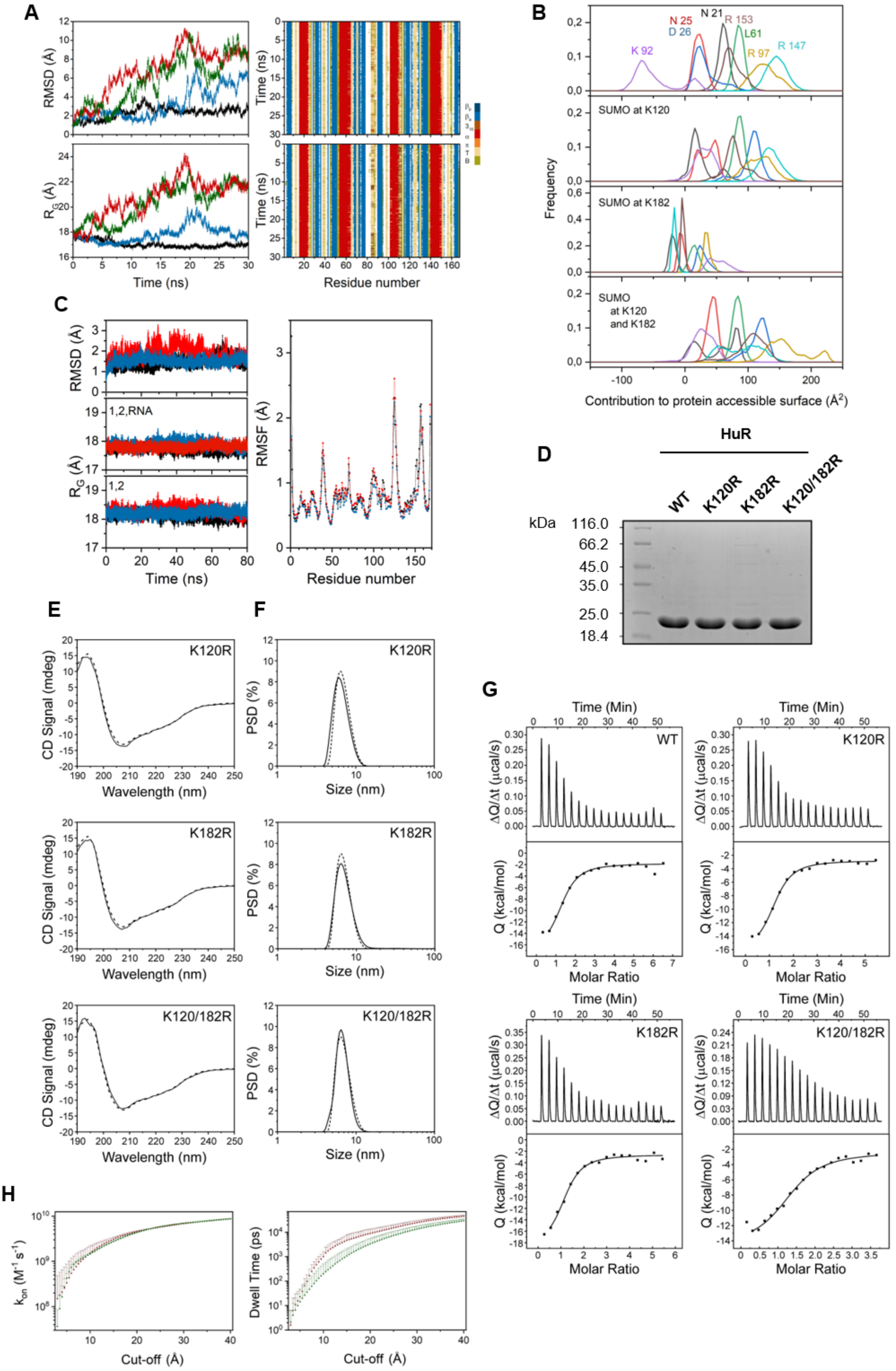
A
RMSD (Å)
R_G (Å)
Time (ns)
Time (ns)
Residue number
B
N 25
D 26
N 21
R 153
L61
K 92
R 97
R 147
SUMO at K120
SUMO at K182
SUMO at K120 and K182
Frequency
Contribution to protein accessible surface (Å2)
C
1,2,RNA
1,2
RMSD (Å)
R_G (Å)
RMSF (Å)
Time (ns)
Residue number
D
HuR
WT
K120R
K182R
K120/182R
kDa
116.0
66.2
45.0
35.0
25.0
18.4
E
K120R
K182R
K120/182R
CD Signal (mdeg)
Wavelength (nm)
F
PSD (%)
Size (nm)
G
Time (Min)
WT
K120R
K182R
K120/182R
ΔQ/Δt (µcal/s)
Q (kcal/mol)
Molar Ratio
H
k_on (M-1 s-1)
Dwell Time (ps)
Cut-off (Å)

Figure S6. (A) Statistics of molecular dynamics (MD) computations. *Upper left panel*, time course for the RMSD values along the trajectories. *Black* stands for the untouched RRM1-2, *blue* for the construct SUMOylated at K182, *red* for SUMOylation at K120 and *green* for the double modification. *Lower left*, evolution of the radius of gyration of HuR RRM1-2 along the trajectories, excluding SUMO molecules. The colour code used is the same as in the *upper panel*. *Right panels* show the secondary structure of the HuR RRM12 construct, excluding SUMO molecules, along the trajectories computed for the unmodified (*upper*) and the doubly SUMOylated protein (*lower*). Colours indicate, according to the right legend, different secondary structures as defined in the DSSP program. **(B)** Contribution of key target residues to the total solvent accessible surface of the RRM1-2 moiety. Frequency distributions of accessible surfaces along the whole MD trajectories. All selected residues establish different kind of interactions with a poly-U RNA oligo, according to the XRD data.[1] **(C)** MD computations on the HuR RRM1-2 tandem with an 11-mer *c-fos* mRNA segment. Starting structure was that reported by Wang et al. (PDB code 4ED5).[1] Data in *black*, *blue* and *red* correspond to WT, K120R and K182R trajectories, respectively. The root-mean-square deviation (RMSD) values below 3 Å indicate that none of the domains undergoes any significant change, and that the two RRM domains keep their relative orientation. The radius of gyration was measured for the coordinates of both, protein and RNA (1,2, RNA), or only the protein moiety (1,2) along the whole trajectories. Radius of gyration (R_G) values are lower when considering both, protein and RNA, since the latter is inserted in a cleft between the two domains and the linker, instead of covering an open surface. The constant R_G values clearly indicate that the insertion of RNA between the two domains makes the whole complex rigid. The lack of differences between the WT construct and the mutant species is consistent with the fact that K120 locates far from the binding site, and that K182 only contributes with a single H-bond at the 5' end. **(D)** Recombinant HuR protein constructs expression and purity assessment by SDS-PAGE analysis. Coomassie Blue-stained 15% gel loaded with 25 µg of protein samples. The lanes show single bands slightly below 25 kDa, which is consistent with the theoretical molecular weight of the constructs (*ca.* 22.8 kDa). **(E)** Far-UV circular dichroism (CD) spectra of HuR RRM1-2 species at 10 µM. The WT protein profile is shown as a dashed line in all spectra, whereas the profile of the mutants is presented as a solid line superimposed on the WT spectrum for

comparison purposes. The CD measurements do not show significant differences between HuR RRM1-2 constructs, suggesting that the secondary structure of all of them is essentially identical. **(F)** Dynamic light scattering (DLS) analysis to study protein homogeneity. Volume-weighted particle size distributions (PSDs) are given for each HuR RRM1-2 construct at 1 mg/ml. The WT protein profile is shown as a dashed line in all spectra, whereas the profile of the mutants is presented as a solid line superimposed on the WT graph for comparison purposes. The graphs show nearly identical PSDs for all constructs, demonstrating that the point mutations introduced into HuR RRM1-2 have no effect on its aggregation state. **(G)** Isothermal titration calorimetry (ITC) measurements of T-rich DNA binding to HuR RRM1-2 species. 150-180 μM of each HuR RRM1-2 construct were titrated to 10-15 μM of T-rich DNA. Thermograms and binding isotherms are shown in the *upper* and *lower* panels, respectively. The curve through the points represents the best fit to a one-site binding model. ITC data analysis provide very similar K_D values for all HuR RRM1-2 constructs, indicating that the SUMOylation mutants assayed preserve their ability to bind nucleic acid virtually intact. **(H)** Derivation of binding and dissociation kinetics from the Brownian dynamics (BD) rigid-body diffusion trajectories. For each set of computations, two curves are represented, corresponding to two independent pair-distances criteria for reaction.[2] *Upper graph*, binding rates for the WT (*green*) and K120/182R (*red*). *Lower graph*, residence times at different distance cut-offs for the two interactions. Error bars indicate standard deviation.

(D) Western blots are representative of at least three biological replicates.

Related to **Figure 2**.

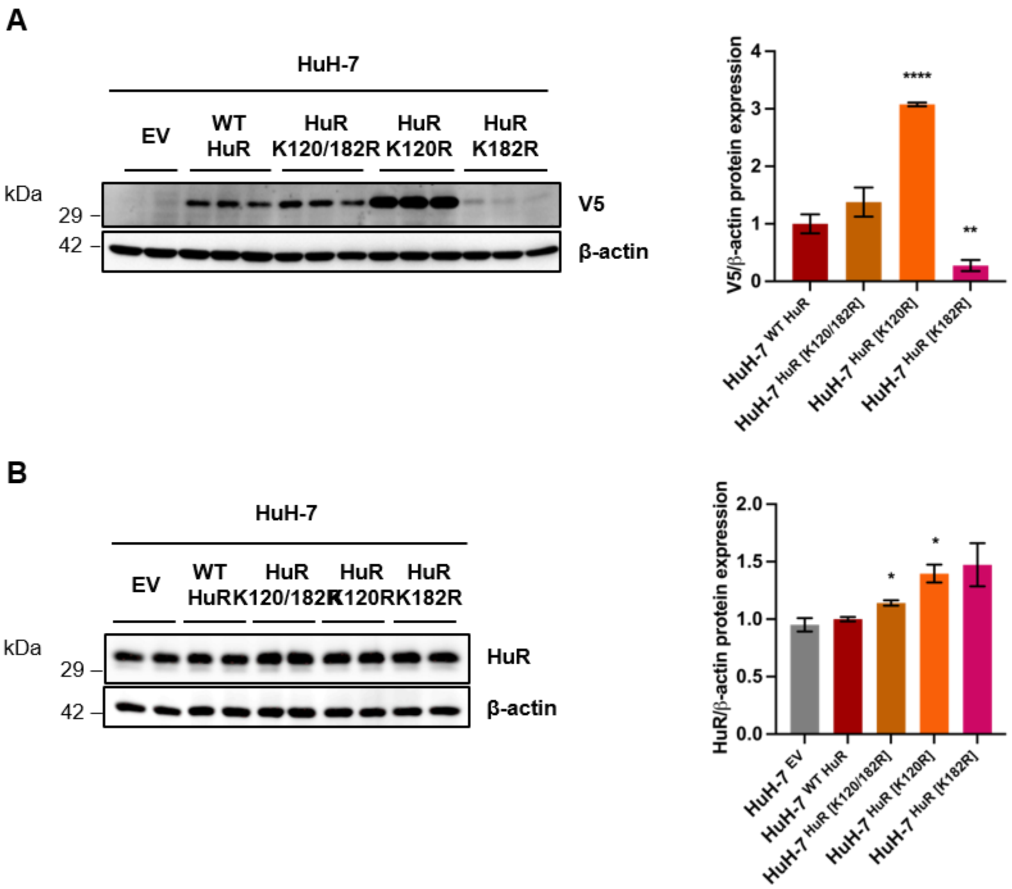

Figure S7. (A) V5-HuR and **(B)** total HuR protein expression levels and quantification after stable transfection of WT HuR as well as the K120/182R, K120R, K182R HuR mutant species in the HuH-7 cell line, relative to β-actin.

(A and B) Data are represented as the mean ± SD of at least three biological replicates within one representative experiment. *$p < 0.05$, **$p < 0.01$, ***$p < 0.001$ and ****$p < 0.0001$, two-tailed t-test vs. HuH-7 $^{\text{WT HuR}}$. If not indicated otherwise, the differences were n.s.

Related to **Figure 3**.

A

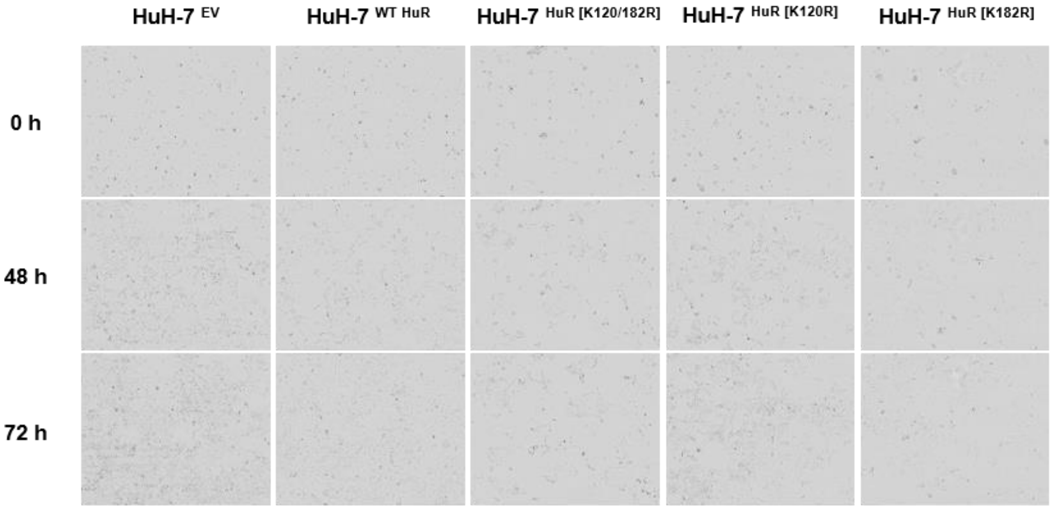

p-value Compared to HuH-7 WT HuR	24h	48h	72h	96h
HuH-7 EV	*n.s.*	*n.s.*	*n.s.*	*n.s.*
HuH-7 HuR [K120/182R]	****	****	****	****
HuH-7 HuR [K120R]	****	****	****	****
HuH-7 HuR [K182R]	****	****	****	****

B

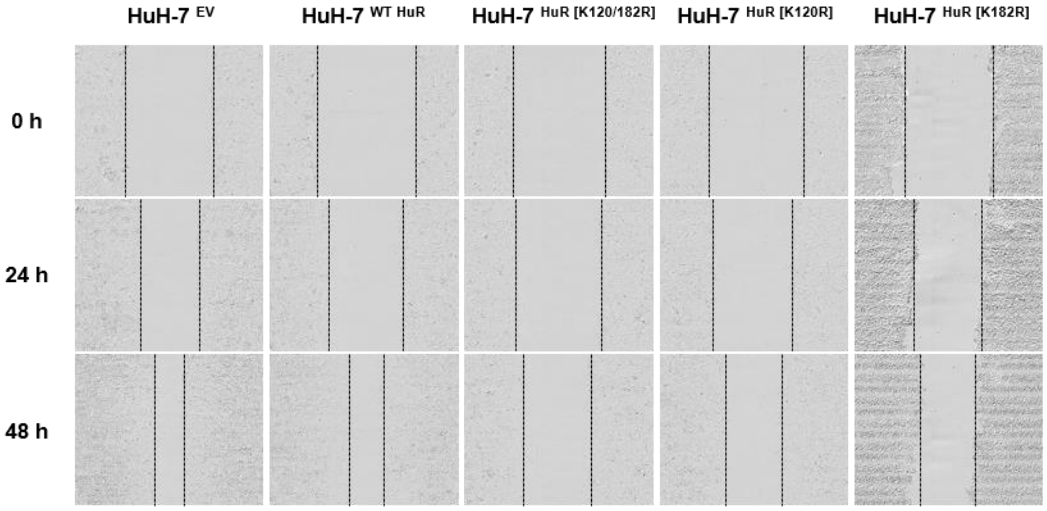

p-value Compared to HuH-7 WT HuR	6h	12h	24h	36h	48h	60h
HuH-7 EV	*n.s.*	*n.s.*	*n.s.*	*n.s.*	**	****
HuH-7 HuR [K120/182R]	***	****	****	****	****	****
HuH-7 HuR [K120R]	****	****	****	****	****	****
HuH-7 HuR [K182R]	*	***	****	****	****	****

Figure S8. Representative pictures and statistical significance of **(A)** cell proliferation and **(B)** scratch-wound process of HuH-7 cell lines stably expressing WT HuR and the different SUMOylation mutants analysed in the IncuCyte system.

(A-B) Images are representative of at least three biological replicates. *$p < 0.05$, **$p < 0.01$, ***$p < 0.001$ and ****$p < 0.0001$, two-tailed t-test vs. HuH-7 $^{\text{WT HuR}}$.

Related to **Figure 3**.

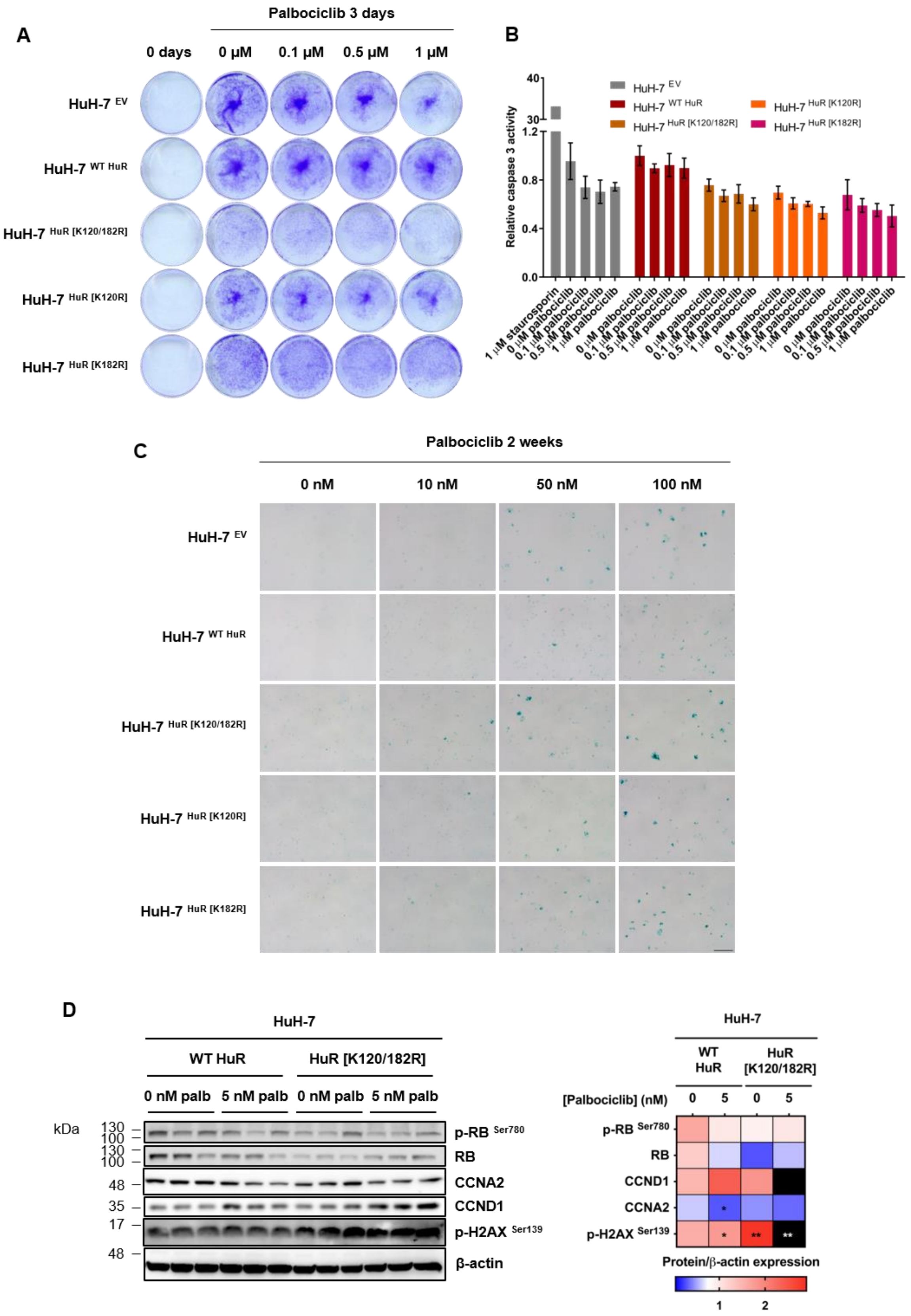

Figure S9. (A) Crystal violet staining of the HuH-7 cell lines stably expressing WT HuR and the K120/182R, K120R, K182R SUMOylation mutants treated with the indicated doses of palbociclib for 3 days. **(B)** Quantification of cell apoptosis in the

HuH-7 cell lines stably expressing WT HuR and the different SUMOylation mutants after an acute 3-day treatment with a range of palbociclib concentrations, analysed by caspase 3 activity assay. **(C)** Representative pictures of senescence associated β-galactosidase staining in the HuH-7 cell lines stably expressing the WT and the K120/182R, K120R, K182R HuR mutant species after a chronic 2-week treatment with a range of palbociclib concentrations. **(D)** p-RB ^{Ser780}, RB, CCND1, CCNA2 and p-H2AX ^{Ser139} protein expression levels and quantification in the HuH-7 cell lines stably expressing WT HuR and the K120/182R SUMOylation mutant after a chronic 2-week treatment with 5 nM palbociclib, relative to β-actin. In the heatmap, *red* indicates high protein expression levels, *blue* indicates low, and values outside the defined range are represented in *black*.

(A-C) Images are representative of at least three biological replicates. **(C)** Scale bar is 200 µm.

(B and D) Data are represented as the mean ± SD of at least three biological replicates within one representative experiment. **p* < 0.05 and ***p* < 0.01, two-tailed t-test vs. 0 nM palbociclib or HuH-7 $^{WT\ HuR}$. If not indicated otherwise, the differences were n.s.

Related to **Figure 3**.

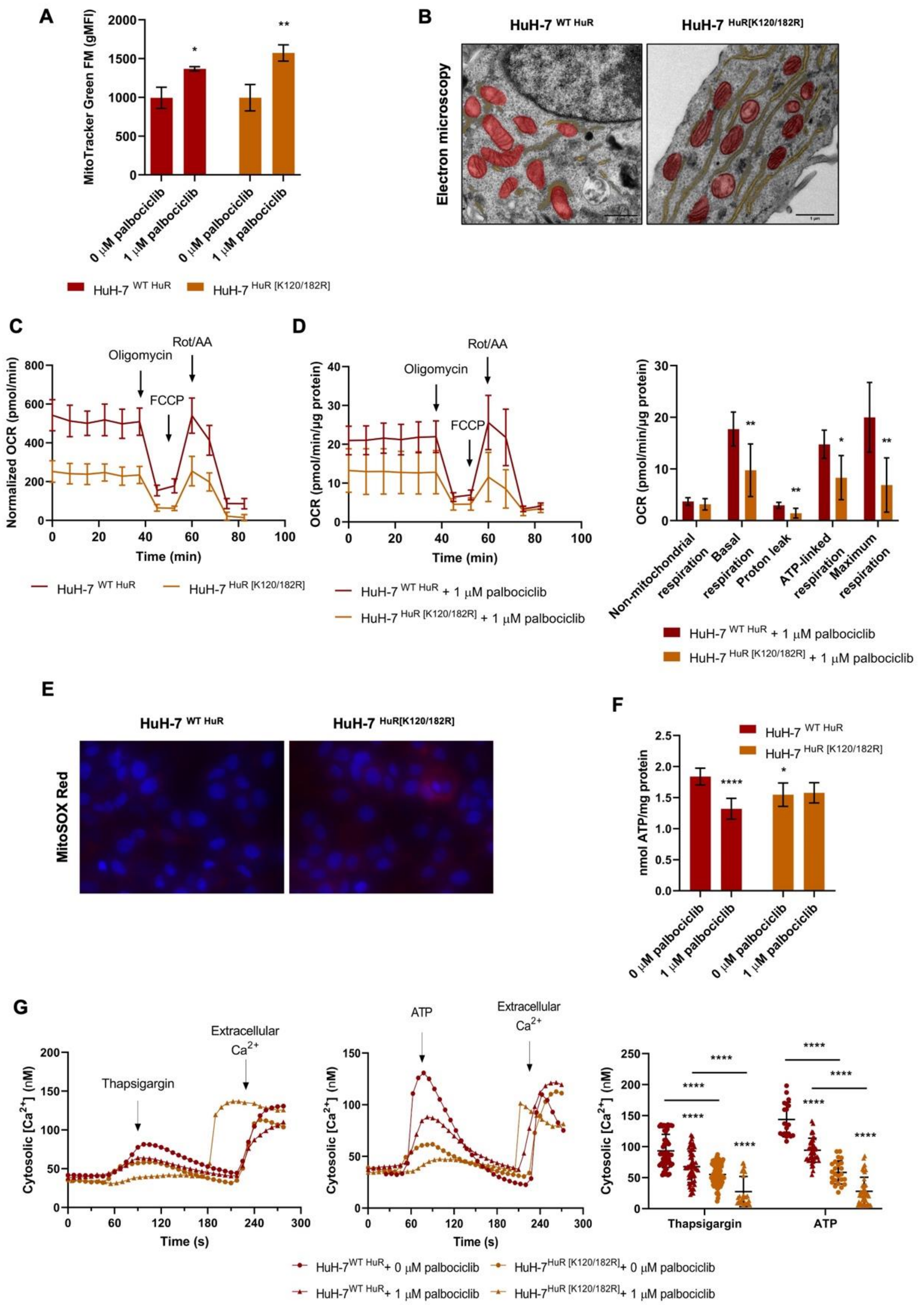

Figure S10. (A) Quantification and representative histograms from flow cytometry analysis of MitoTracker Green FM staining in the WT HuR and K120/182R HuR SUMOylation mutant HuH-7 cells treated with 1 μM palbociclib for 3 days. **(B)** Segmentation masks of mitochondria (*red*) and ER (*yellow*) in electron microscopy images of WT and K120/182R HuR expressing HuH-7 cells. Scale bar is 1 μm. **(C)** Seahorse-based monitoring of the oxygen consumption rate (OCR) after treatment

with oligomycin, carbonyl cyanide 4-trifluoromethoxy-phenylhydrazone (FCCP) and a combination of rotenone and antimycin A in the HuH-7 cell lines stably expressing WT HuR and the K120/182R HuR SUMOylation mutant. **(D)** Seahorse-based monitoring of the OCR after treatment with oligomycin, FCCP and a combination of rotenone and antimycin A, and quantification of mitochondrial respiration parameters in the HuH-7 cell lines stably expressing WT HuR and the K120/182R HuR SUMOylation mutant subjected to a 3-day regimen with 1 µM palbociclib. **(E)** Representative images of MitoSOX Red mitochondrial superoxide indicator staining in the WT and the K120/182R HuR expressing HuH-7 cells. **(F)** Determination of total ATP content in the WT and HuR SUMOylation mutant HuH-7 cells treated with 1 µM palbociclib for 3 days. **(G)** Representative curves and quantification of cytosolic Ca^{2+} levels with Fura-2 AM fluorescent probe labelling after stimulating Ca^{2+} release from the ER with thapsigargin and ATP, and addition of extracellular Ca^{2+} in the WT and SUMOylation mutant HuR variant expressing HuH-7 cells treated with 1 µM palbociclib during 3 days.

(B and E) Images are representative of at least three biological replicates. **(B)** Scale bar is 1 µm.

(A, C, D, F and G) Data are represented as the mean ± SD of at least three biological replicates within one representative experiment. **p* < 0.05, ***p* < 0.01, ****p* < 0.001 and *****p* < 0.0001, two-tailed t-test vs. 0 nM palbociclib or HuH-7 $^{WT\ HuR}$. If not indicated otherwise, the differences were n.s.

Related to **Figure 5**.

Table S1. Summary of clinical data from a cohort of patients with HCC obtained from the Andalusian Biobank (Córdoba Node). Related to STAR Methods.

**Patients [n]**	86
**Age, y [median (IQR)]**	60.6 (64-67)
**Etiology [n (%)]**	
**HCV**	30 (36.1)
**Alcohol**	21 (25.3)
**HBV**	11 (13.3)
**Other**	5 (6)
**HCV + Alcohol**	9 (10.8)
**HBV + Alcohol**	1 (1.2)
**HCV + other**	3 (3.6)
**Alcohol + other**	0 (-)
**Histological differentiation [n (%)]**	
**Well differentiated**	30 (35.3)
**Moderately differentiated**	50 (58.8)
**Poorly differentiated**	5 (5.9)
**Portal Hypertension [n (%)]**	44 (5.2)
**Microvascular invasion [n (%)]**	33 (39.8)
**Treated before surgery [n (%)]**	23 (26.4)
**Recurrence [n (%)]**	39 (47)
**Death [n (%)]**	50 (61)

Table S2. Gene primer sequences used for qPCR and purchased from Sigma-Aldrich. Related to STAR Methods.

Gene	Species	Sequence	
ACTB	*Homo sapiens*	Forward primer	5'-ACTCTTCCAGCCTTCCTTCC-3'
		Reverse primer	5'-CAGTGATCTCCTTCTGCATC-3'
GAPDH	*Homo sapiens*	Forward primer	5'-GCCTCAAGATCATCAGCAATG-3'
		Reverse primer	5'-CTTCCACGATACCAAAGTTGT-3'
HPRT1	*Homo sapiens*	Forward primer	5'-CTTTGCTGACCTGCTGGAT-3'
		Reverse primer	5'-ATGTCCCCTGTTGACTG-3'
RPLP0	*Homo sapiens*	Forward primer	5'-CGACCTGGAAGTCCAACTAC-3'
		Reverse primer	5'-ATCTGCTGCATCTGCTTG-3'
SUMO1	*Homo sapiens*	Forward primer	5'-GGTCTGGACCAAAAGAAGAGGA-3'
		Reverse primer	5'-TCAGTGAAGCCATCTTTGGAGT-3'
SUMO2	*Homo sapiens*	Forward primer	5'-AGATTCCGATTTGACGGGCA-3'
		Reverse primer	5'-CAGTAGACACCTCCCGTCTG-3'
SUMO3	*Homo sapiens*	Forward primer	5'-ACACCATCGACGTGTTCCAG-3'
		Reverse primer	5'-CGGGCCCTCTAGAAACTGTG-3'
SUMO4	*Homo sapiens*	Forward primer	5'-TCAGATTCCGATTTGGTGGG-3'
		Reverse primer	5'-CCTCCCGTAGGCTGTTGAAA-3'
SAE1	*Homo sapiens*	Forward primer	5'-CTCACCATTCCTGCCACCAT-3'
		Reverse primer	5'-AGACAACTGGAGCCAGAAGC-3'
UBA2	*Homo sapiens*	Forward primer	5'-CGCCTGGTATGTCTGTGTAAGA-3'
		Reverse primer	5'-ACTGTGCAGGCATGTGTAAC-3'
UBC9	*Homo sapiens*	Forward primer	5'-CAAGACCCAGCTCAAGCAGA-3'
		Reverse primer	5'-TGTGCTCGGACCCTTTTCTC-3'
PIAS1	*Homo sapiens*	Forward primer	5'-CGGACAGTGCGGAACTAAAG-3'
		Reverse primer	5'-CAAGTTCGTGTTTGCGTCCG-3'
PIAS2	*Homo sapiens*	Forward primer	5'-ACGCCGATATCCACGAACTC-3'
		Reverse primer	5'-GGTGATGAGCCACCATCCAA-3'
PIAS3	*Homo sapiens*	Forward primer	5'-GGAGCTGGGCGAATTAAAGC-3'
		Reverse primer	5'-GTGCTTCCGTCCACTCTTGT-3'
PIAS4	*Homo sapiens*	Forward primer	5'-GCTGGTGGAGGCCAAAAACAT-3'
		Reverse primer	5'-GGGCTACAGTCAAACTGCAC-3'
SENP1	*Homo sapiens*	Forward primer	5'-CGAGCACGAGAAAGATTGCG-3'
		Reverse primer	5'-ACTGAATGTTCCCGCTCCTG-3'
SENP2	*Homo sapiens*	Forward primer	5'-CTTTGCCTGGTCCCTCTAGC-3'
		Reverse primer	5'-GTGAGAGGCCTTCATGCACT-3'
SENP3	*Homo sapiens*	Forward primer	5'-CCGACCCTCTTTTGATGCCT-3'
		Reverse primer	5'-CAGCTGACTCCATCTTGGGG-3'
SENP5	*Homo sapiens*	Forward primer	5'-CCCAGCACTTTCCTCTCCTG-3'
		Reverse primer	5'-TAACGCTGACAGAACCCCAC-3'
SENP6	*Homo sapiens*	Forward primer	5'-TTCTGGAAGCTTTGGCTAGATCA-3'
		Reverse primer	5'-GCAGATTTGTCCCATCTTTATCTGT-3'
SENP7	*Homo sapiens*	Forward primer	5'-GCGGTTGCTACTCCCTTTCT-3'
		Reverse primer	5'-CCCCCTTAGTAGGTGGTGGA-3'
Gapdh	*Mus musculus*	Forward primer	5'-TTGATGGCAACAATCTCCAC-3'
		Reverse primer	5'-CGTCCCGTAGACAAAATGG-3'
Pias2b	*Mus musculus*	Forward primer	5'-TCCCCAGTACTGTCCTCCTA-3'
		Reverse primer	5'-TGACAGACGTACTGCTTGCT-3'
Senp1	*Mus musculus*	Forward primer	5'-CGGCGAGATGCATTCACAAG-3'
		Reverse primer	5'-GTTCCCTCACCCCTTCACAG-3'
Senp2	*Mus musculus*	Forward primer	5'-GGCAGCTGATCAGAGGTTGT-3'

		Reverse primer	5'-ACAGGCCCAGCTTCCATAAC-3'
Senp3	*Mus musculus*	Forward primer	5'-TGGCAGAGGATGGGATGAGA-3'
		Reverse primer	5'-GTCAGGGTCCAGAGGAGACT-3'

Table S3. Thermodynamic interaction parameters for T-rich DNA binding to HuR RRM1-2 species. K_D stands for dissociation constant, ΔH, ΔS and ΔG are the thermodynamic parameters for the association process. Related to **Figure 2**.

**Titrant**	**Ligand**	**n**	**K_D (μM)**	**ΔH (kcal/mol)**	**ΔG (kcal/mol)**	**$T\Delta S$ (kcal/mol)**
**T-rich DNA**	**HuR RRM1-2 WT**	1.26 ± 0.04	1.68 ± 0.32	-15.27 ± 1.00	-7.88 ± 0.11	-7.39 ± 1.01
	**HuR RRM1-2 K120R**	1.17 ± 0.04	1.64 ± 0.34	-13.51 ± 0.87	-7.89 ± 0.12	-5.62 ± 0.88
	**HuR RRM1-2 K182R**	1.13 ± 0.06	1.73 ± 0.57	-15.94 ± 1.72	-7.86 ± 0.19	-8.08 ± 1.73
	**HuR RRM1-2 K120/182R**	1.36 ± 0.04	2.57 ± 0.65	-12.07 ± 0.87	-7.62 ± 0.15	-4.44 ± 0.88

SUPPLEMENTAL REFERENCES

1. Wang, H., Zeng, F., Liu, Q., Liu, H., Liu, Z., Niu, L., Teng, M., and Li, X. (2013). The structure of the ARE-binding domains of Hu antigen R (HuR) undergoes conformational changes during RNA binding. Acta Crystallogr D Biol Crystallogr *69*, 373–380. 10.1107/S0907444912047828.
2. Elcock, A.H., Gabdoulline, R.R., Wade, R.C., and McCammon, J.A. (1999). Computer simulation of protein-protein association kinetics: acetylcholinesterase-fasciculin11Edited by B. Honig. J Mol Biol *291*, 149–162. https://doi.org/10.1006/jmbi.1999.2919.
